# Polyethylene-Like Blends Amenable to Abiotic Hydrolytic Degradation

**DOI:** 10.1021/acssuschemeng.2c07537

**Published:** 2023-03-13

**Authors:** Marcel Eck, Léa Bernabeu, Stefan Mecking

**Affiliations:** Department of Chemistry, University of Konstanz, Universitätsstr. 10, 78457 Konstanz, Germany

**Keywords:** Polyethylene-like, degradable, biobased, long-chain polyester, polymer blend, 3D printing

## Abstract

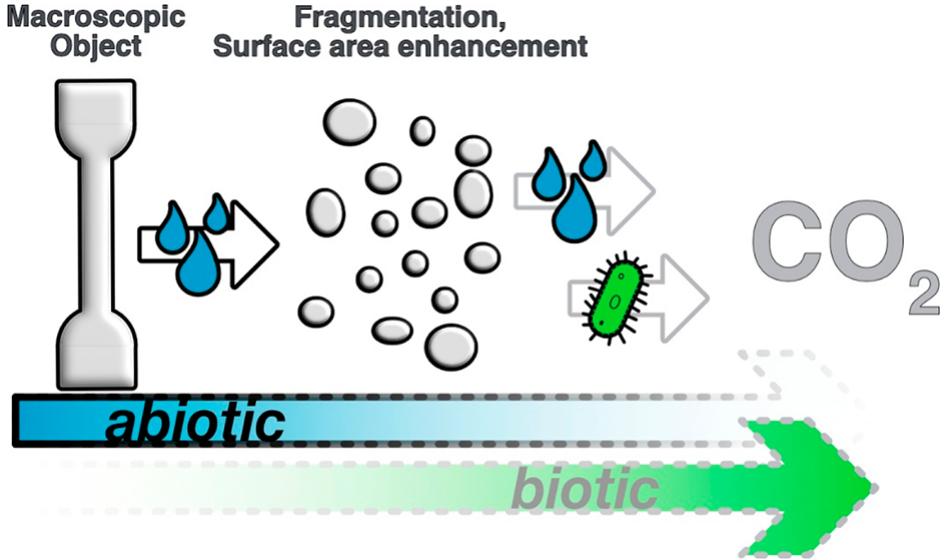

Long-chain aliphatic polyester-18,18 (PE-18,18) exhibits high density polyethylene-like material properties and, as opposed to high density polyethylene (HDPE), can be recycled in a closed loop via depolymerization to monomers under mild conditions. Despite the in-chain ester groups, its high crystallinity and hydrophobicity render PE-18,18 stable toward hydrolysis even under acidic conditions for one year. Hydrolytic degradability, however, can be a desirable material property as it can serve as a universal backstop to plastic accumulation in the environment. We present an approach to render PE-18,18 hydrolytically degradable by melt blending with long-chain aliphatic poly(H-phosphonate)s (PP). The blends can be processed via common injection molding and 3D printing and exhibit HDPE-like tensile properties, namely, high stiffness (*E* = 750–940 MPa) and ductility (ε_tb_ = 330–460%) over a wide range of blend ratios (0.5–20 wt % PP content). Likewise, the orthorhombic solid-state structure and crystallinity (χ ≈ 70%) of the blends are similar to HDPE. Under aqueous conditions in phosphate-buffered media at 25 °C, the blends’ PP component is hydrolyzed completely to the underlying long-chain diol and phosphorous acid within four months, as evidenced by NMR analyses. Concomitant, the PE-18,18 major blend component is partially hydrolyzed, while neat PE-18,18 is inert under identical conditions. The hydrolysis of the blend components proceeded throughout the bulk of the specimens as confirmed by gel permeation chromatography (GPC) measurements. The significant molar mass reduction upon extended immersion in water (*M*_n_(virgin blends) ≈ 50–70 kg mol^–1^; *M*_n_(hydrolyzed blends) ≈ 7–11 kg mol^–1^) resulted in embrittlement and fragmentation of the injection molded specimens. This increases the surface area and is anticipated to promote eventual mineralization by abiotic and biotic pathways of these HDPE-like polyesters in the environment.

## Abstract 2

Renewable HDPE-like polymer blends endowed with hydrolytic degradability can serve as a universal backstop to accumulation in the environment.

## Introduction

Plastics released to the environment can persist for many decades or longer. Even in conjunction with a more responsible, circular plastics econonomy, degradability is desirable as a backstop to plastic accumulation in the environment.^1−8^ The degradation rates of polymer materials depend strongly on their specific environment. Especially rates of biodegradation vary enormously depending on the microbial environment.^9^ Therefore, the abiotic, hydrolytic degradability of materials is of interest. It is expected to be slow compared to biodegradation at optimum conditions but can be a more universal approach to prevent long-term persistency and accumulation.

In the case of high density polyethylene (HDPE), hydrolytic degradation in the environment is hindered by its chemically inert and hydrophobic hydrocarbon nature as well as by its high crystallinity. We have shown that low densities of ester or carbonate groups in polyethylene chains can facilitate closed-loop chemical recycling under mild conditions while retaining HDPE-like materials properties.^10^ These in-chain functional groups in principle also offer themselves for hydrolytic degradation in the environment. However, the long-chain aliphatic polyester-18,18 (PE-18,18) is stable even to aqueous acids and bases for one year or longer under ambient conditions (cf. Supporting Information Figures S101–S104 for analysis of PE-18,18 exposed to hydrolysis media).^10^ A similar behavior was also found for polyester-15 by Koning and Heise et al. for which no hydrolytic degradation was found to occur over a period of two years.^11^

A variety of approaches aiming for enhancement of the hydrolytic degradability of different polymers were developed. The hydrolytic lability of long-chain aliphatic polycondensates can be enhanced by incorporating more labile moieties such as acetal, orthoester, pyrophosphate, and vitamin C groups into the chain.^12−16^ As a downside, all these groups strongly disturb the crystalline structure of polyethylene and impede desirable material properties, like high ductility and stiffness. Alternatively, hydrolytic degradability can be achieved by installing functionalities capable of chain scission in the polymer backbone. This was shown by Wurm et al. for phosphoester groups with 2-hydroxyethoxy side chains incorporated in a long-chain aliphatic polyphosphoester and PLA.^17,18^ Hillmyer and Ellison et al. demonstrated the incorporation of hydrolytically labile and acid-releasing salicylate units into the chains of PLA as an elegant means of enhancing its hydrolytic degradation rate.^19^ As an alternative to such chemical modifications, a degradation enhancing polymeric component can also be introduced physically by melt blending.^20^ Blends of long-chain aliphatic polycondensates as a minor component into a PLA^21−23^ or PBAT^24^ matrix have been studied with the aim of enhancing mechanical properties. For these blends, superior mechanical properties can be achieved by utilizing compatibilization strategies which improve the interfacial adhesion of the otherwise immiscible blend components.^25^ Degradability of the blends, in which the polyethylene-like polyesters are also a minor component, was not a focus.

We now report an approach to render the highly crystalline long-chain aliphatic polyester PE-18,18 hydrolytically degradable which at the same time does not impair the polymer’s desirable HDPE-like materials properties. Melt blending with small amounts of long-chain aliphatic poly(H-phosphonate)s as degradation-enhancing components facilitates hydrolytic breakdown under environmentally relevant conditions.

## Experimental Section

### Materials

All chemicals were used as received without further purification. 1,18-Octadecanedioic acid was purchased from Elevance Renewable Sciences, Inc. C_18_ dimethylester, C_18_ diol, PE-18,18, and C_26_ diol were prepared by reported procedures.^10,26^ Lithium hydride (95%) and diethyl phosphite (98%) were purchased from Sigma-Aldrich. Sodium chloride (≥99.5%), phosphate buffer (pH 7, Rotistar), and disodium hydrogen phosphate dihydrate (≥98%) were purchased from Carl Roth. Sodium hydroxide solution (aqueous, 1 M), sulfuric acid (Titrisol for 1 L, 0.5 M), hydrochloric acid (aqueous, 1 M), sodium bromide (≥99.0%), and sodium dihydrogen phosphate monohydrate (for analysis) were purchased from Merck. Potassium dihydrogen phosphate (for analysis) was purchased from Riedel de-Haën. High density polyethylene Purell GB 7250 from LyondellBasell was used as reference material. Deuterated solvents for NMR spectroscopy were purchased from Eurisotop and dried over molecular sieves from Riedel de-Haën (0.4 nm). All manipulations involving air- and/or moisture-sensitive substances were carried out under inert atmospheres using standard Schlenk and glovebox techniques.

### Polymerization Experiments

Poly(H-phosphonate)s (poly(H-phosphonate)-18, PP-18, and poly(H-phosphonate)-26, PP-26) were obtained according to a reported procedure.^27^ A long-chain diol (1.0 equiv) and a stir bar were added into a Teflon inlet placed in a three-necked Schlenk tube and dried at 60 °C under vacuum. A cooled condensor flask that allows for monitoring of the volatiles released from the polymerization mixture was connected. LiH (1 mol % vs monomer, rather than Na catalyst previously reported) and diethyl phosphite (1.1 equiv) were added, and the temperature was increased to 180 °C (stirring at 500 rpm). Oligomerization commenced, and vacuum was gradually applied (900 to 10 mbar) over the course of 1 h. The polymerization step was conducted at 180 °C for typically 5 h at 2 × 10^–2^ mbar vacuum. After cooling to room temperature, the resulting polymer was retrieved from the Teflon inlet using a tweezer and stored under inert atmosphere without further workup.

### Compounding of PE-18,18/Long-Chain Poly(H-phosphonate) Blends

PE-18,18/PP-18 blends were compounded in a Xplore MC 15 micro compounder at 160 °C and 50 rpm. The neat PE-18,18 was compounded for 5 min. Subsequently, PP-18 was added in small portions, and the mixture was homogenized for a further 20 min. Injection molded test specimens were prepared using a Xplore IM 5.5 micro injection molder. The cylinder and mold temperatures were set to 160 and 60 °C, respectively, and an injection pressure of 16 bar for 10 s and 12 bar for 15 s was applied. Blends with PP-18 contents below 2 wt % were compounded for 15 min in a single step by adding the premixed polymers into the compounder.

PE-18,18/PP-26 blends were prepared analogously but using a Xplore MC 5 micro compounder under vacuum at 160 °C and 50 rpm. The neat PE-18,18 was compounded for 20 min. Subsequently, PP-26 was added in small portions, and the mixture was homogenized for a further 10 min. Injection molded test specimens were prepared using a Thermo Scientific HAAKE MiniJet Pro micro injection molder. The cylinder and mold temperatures were set to 160 and 50 °C, respectively, and injection pressures of 500 bar for 15 s and 350 bar for 5 s were applied.

### Hydrolysis Experiments

Four rectangular specimens (10 mm × 7 mm × 1 mm, approximately 85 mg) of each PE-18,18/PP-26 blend were cut from injection molded bars and placed in 8 mL glass vials. Four different degradation media (5 mL) were added (Milli-Q water; 0.5 M H_2_SO_4_; 67 mM phosphate buffer, pH 7; 1 M NaOH), and the vials were sealed. In addition to the defined rectangular samples, each experiment was also set up with a smaller specimen fitting on a stub for SEM analysis. The sealed vials were attached to a holder and placed on an orbital shaker (200 rpm) in a light-proof Peltier temperature-controlled cabinet (25.0 °C). The hydrolysis experiments were conducted for three different durations of 16, 32, and 48 weeks. After this time, the samples were removed from the medium, stored in deionized water overnight to remove residual inorganic substances, and subsequently dried under vacuum at 50 °C. Prior to further analysis, the specimens were weighed and photographed.

The hydrolysis experiments for the PE-18,18/PP-18 blends (i.e., 0.5, 2, and 10 wt % PP-18) were conducted analogously as outlined for the PE-18,18/PP-26 blends on rectangular specimens in four different degradation media (Milli-Q water; 0.5 M H_2_SO_4_; phosphate buffer (pH 8, 100 mM, salinity = 0.6 M); 1 M NaOH) for a single duration of 16 weeks.

The hydrolysis experiments for neat PE-18,18 were conducted analogously as outlined for the PE-18,18/PP-26 blends on rectangular specimens in four different degradation media (Milli-Q water; 1 M HCl; phosphate buffer (pH 7, from Rotistar); 1 M NaOH) for a single duration of 48 weeks.

### Aqueous Exposure of Test Bar Specimens

Two tensile test specimens (ISO 527-2, type 5A) of each material investigated were immersed in ca. 15 L of deionized water. The sealed containers were stored in a Peltier temperature-controlled cabinet at 25.0 °C for 16 weeks. After this time, the tensile test specimens were left to dry under ambient conditions prior to tensile testing.

### Stability of Test Bar Specimens in Air under Ambient Conditions

Three tensile test specimens (ISO 527-2, type 5A) of each material investigated were stored in a sealed container at 60% humidity and 25.0 °C for 4 weeks. The humidity in the container was adjusted by utilizing the equilibrium vapor pressure of a saturated aqueous solution of NaBr.^28^ The temperature was set by storing the container in a light-proof Peltier temperature-controlled cabinet. When the experiments were finished, the mechanical properties of the tensile test specimens were investigated by tensile testing.

### Characterization and Processing

Nuclear magnetic resonance (NMR) spectra were recorded on a Bruker Avance III HD 400 spectrometer. Chemical shifts were referenced to the resonance of the solvent (residual proton resonances for ^1^H spectra, carbon resonances for ^13^C spectra). Mestrenova software by Mestrelab Research S.L. (version 14.1.2) was used for data evaluation.

Molar masses of polymers and blends were determined by gel permeation chromatography (GPC) in chloroform at 35 °C on a PSS SECcurity^2^ instrument, equipped with PSS SDV linear M columns (2 cm × 30 cm, additional guard column) and a refractive index detector (PSS SECcurity^2^ RI). A standard flow rate of 1 mL min^–1^ was used. Molar masses were determined versus low dispersity polystyrene standards (software: PSS WinGPC, version 8.32).

Differential scanning calorimetry (DSC) was carried out on a Netzsch DSC 204 F1 instrument (software: Netzsch Proteus Thermal Analysis, version 6.1.0) with a heating/cooling rate of 10 K min^–1^. Data reported are from second heating cycles.

Scanning electron microscopy (SEM) images were recorded on a Zeiss Gemini 500 or a Zeiss Auriga microscope by secondary electron (SE2) or in-lense detection with an acceleration voltage of 3 or 5 kV. Polymer samples were sputtered with a 12 nm platinum layer using a Quorum Q150 sputter coater. Filament samples were sputtered with an 8 nm gold layer using an Edwards Scancoat Six Pirani 501 sputter coater.

Light microsocopy images were recorded on a Leica DM4000 M microscope equipped with a Leica DMC2900 camera.

3D printing (fused filament fabrication) of tensile test specimens (ISO 527-2, type 5A) employed a Prusa i3 MK3S+ printer. 3D printed specimens were left to cool on the build plate until a temperature of 30 °C was reached.

Tensile tests were performed on a Zwick Z005/1446 Retroline tC II instrument at a crosshead speed of 5 mm min^–1^ on injection molded or 3D printed tensile test specimens (ISO 527-2, type 5A). The determination of Young’s modulus was conducted at a crosshead speed of 0.5 mm s^–1^. Prior to tensile testing, the samples were preconditioned at room temperature. The Zwick Roell testXpert software version 11.0 was used for data evaluation.

Wide angle X-ray scattering (WAXS) diffractograms were recorded on a D8 Discover instrument (Bruker) with a Vantec or Lynxeye detector on injection molded specimens. Polymer crystallinity (χ_WAXS_) was determined from the WAXS patterns as χ_WAXS_ = [A_c_(110) + A_c_(200)]/[A_c_(110) + A_c_(200) +A_a_], where A_c_ refers to the integrated area of the Bragg reflections from the orthorhombic PE crystal and A_a_ to the integrated area of the amorphous halo. A Voigt fit was used.

Surface free energies were determined on injection molded specimens by the method of Fowkes on a drop shape analyzer DSA25 by KRÜSS.^29^

Thermogravimetric analysis was performed on a Netzsch STA 429 F3 Jupiter. Measurements were performed with 250 mL/min flow rate of N_2_ at a heating rate of 10 K/min from 30 to 750 °C.

## Results and Discussion

### Blends of Polyethylene-Like Polymers

Binary blends of the polyester-18,18 (PE-18,18) main component and poly(H-phosphonate) as a degradation-enhancing minor component were obtained by melt compounding in a twin-screw extruder (cf. Figures S18–S49 for additional characterization data of the blends). Note that long-chain aliphatic poly(H-phosphonate)s have previously been found to be susceptible to rapid hydrolysis even as bulk materials, which also impeded further mechanical characterization.^27^ Two similar poly(H-phosphonate)s differing in the length of the methylene segment were employed (Figure 1). Poly(H-phosphonate)-26 (PP-26, *M*_n_ = 18 kg mol^–1^, *M*_w_ = 43 kg mol^–1^, *T*_m_ = 94 °C, cf. Figures S1–S8 for additional characterization data of PP-26) and poly(H-phosphonate)-18 (PP-18, *M*_n_ = 12 kg mol^–1^, *M*_w_ = 23 kg mol^–1^, *T*_m_ = 73 °C, cf. Figures S9–S16 for additional characterization data of PP-18) were chosen as blend partners based on the similarity of the melting transition and length of the methylene segment, respectively, in comparison to the matrix polymer PE-18,18 (*T*_m_ = 99 °C).

**Figure 1:**
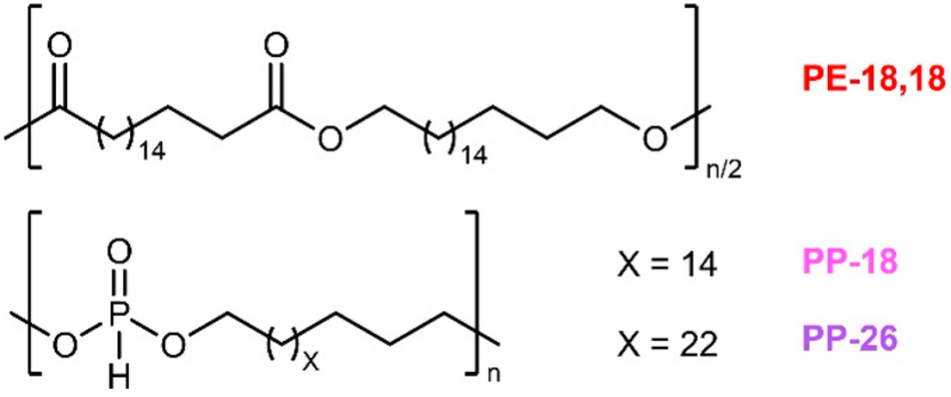
Chemical structures of the major blend component polyester-18,18 (PE-18,18) and the degradation-enhancing poly(H-phosphonate)s-18 and -26 (PP-18, PP-26).

**Figure 2:**
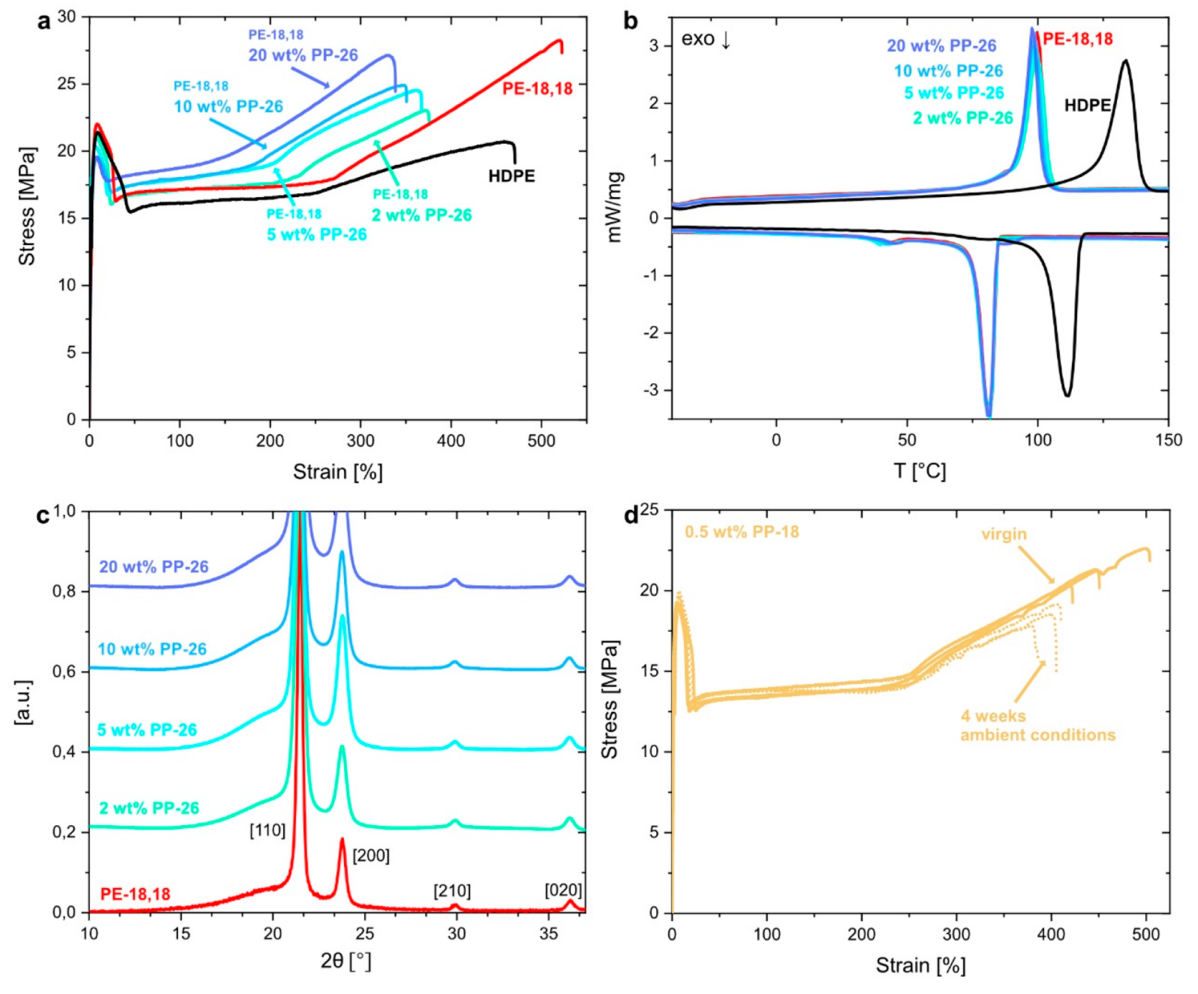
Characterization of polyethylene-like PE-18,18/poly(H-phosphonate) blends. (a) Representative stress–strain curves of injection molded PE-18,18, its 2, 5, 10, and 20 wt % PP-26 blends, and HDPE. (b) DSC traces of PE-18,18, its 2, 5, 10, and 20 wt % PP-26 blends, and HDPE (top, heating trace; bottom, cooling trace). (c) WAXS diffractograms of PE-18,18 and its 2, 5, 10, and 20 wt % PP-26 blends. Diffraction peaks correspond to the orthorhombic unit cell of polyethylene in all cases, and the traces are shifted vertically for clarity. a.u., arbitrary units. (d) Stress–strain curves of injection molded tensile test specimens of PE-18,18 blended with 0.5 wt % PP-18 before and after storage at 25 °C and 60% humidity for 4 weeks.

The HDPE-like material properties of PE-18,18, namely, a high ductility reflected by elongations at break in the range of 330–460% and Young’s moduli in the range of 750–940 MPa, were preserved in the resulting blends for a wide range of ratios (0.5–20 wt % PP-18/26) (Figure 2a). Likewise, the melting points of the blends remained unaltered in comparison to the desirably high melting point of PE-18,18 (*T*_m_ ≈ 100 °C) (Figure 2b, cf. Figures S6 and S15 for the DSC traces of neat PP-26 and PP-18). The preservation of the high crystallinity (χ ≈ 70%) and the orthorhombic solid-state structure are indicative of cocrystallization and miscibility of the long-chain aliphatic polyester and poly(H-phosphonate) components (Figure 2c, cf. Figures S7 and S14 for the WAXS diffractograms of neat PP-26 and PP-18). Note that the stability in air, tested on tensile test specimens of PE-18,18/PP-18 blends stored under controlled conditions, amounts to ≥4 weeks for the 0.5 wt % blend (ε_tb_(virgin) ≈ 460% vs ε_tb_(air) ≈ 400%) (Figure 2d, cf. Figures S110–S115 for additional data on stability of test bar specimens). By comparison, injection molded specimens of pure long-chain aliphatic poly(H-phosphonate)s embrittled within 24 h in air due to hydrolytic degradation.^27^

### 3D Printing

**Figure 3:**
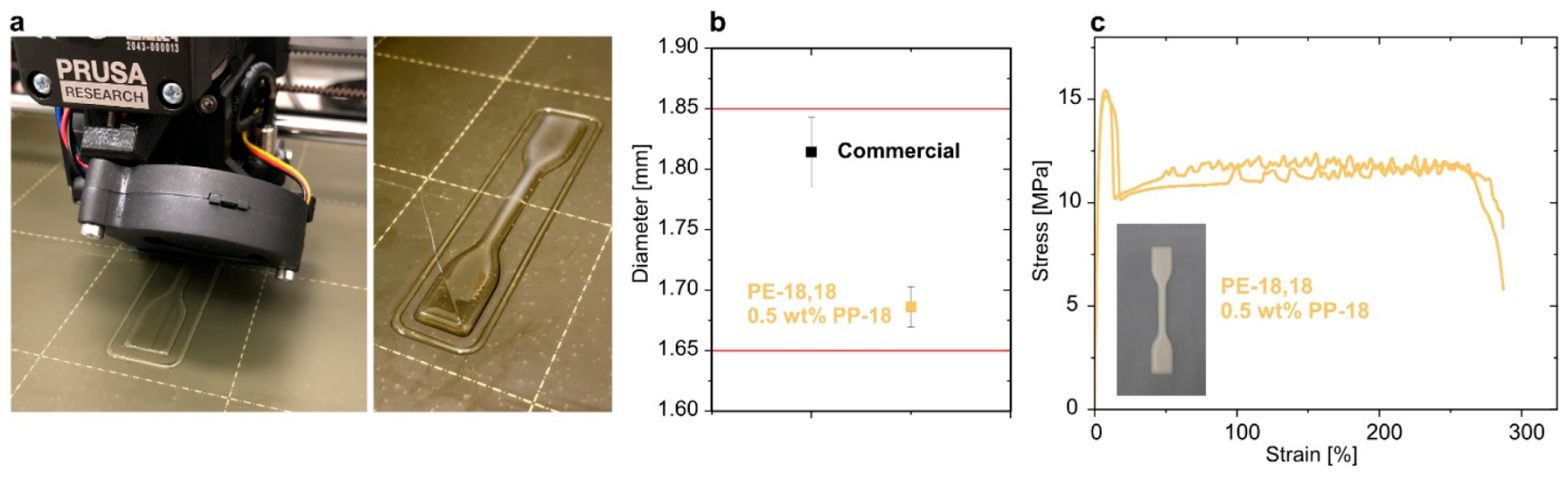
3D printing of PE-18,18 blended with 0.5 wt % PP-18. (a) Fused filament fabrication of a tensile test specimen (ISO 527-2, type 5A, length about 75 mm). (b) Diameter and dimensional accuracy of the extruded blend filament in comparison to commercial polyethylene terephthalate glycol (PETG) filament and specification range for printing on a Prusa i3 MK3S+ printer. Error bars are standard deviations from 40 measurements with a digital caliper. (c) Stress–strain curves of two 3D printed tensile test specimens.

In addition to processing by injection molding, as a complementary advanced and demanding processing technique fused deposition modeling (FDM) was explored. Extrusion of a blend with 0.5 wt % PP-18 after melt compounding through a customized nozzle^10^ yielded high quality filaments with uniform diameters of, e.g., 1.69 ± 0.02 mm, on par with commercial filaments (±0.03 mm) and suited for 3D printing (Figure 3b). Neat PE-18,18 is printable on standard build plate surfaces, unlike HDPE which requires special surfaces.^30^ This desirable feature was also observed for the blend studied (Figure 3a). Despite serrated features during the plastic deformation,^31,32^ a high elongation at break (ε_tb_(3D print) ≈ 290%) is observed in tensile tests on 3D printed specimens (Figure 3c). No evidence for a loss of material performance, e.g., due to premature degradation during the printing process was observed (cf. Figures S54–S56 for additional characterization of 3D printed blend specimens).

### Hydrolytic Degradation

**Figure 4:**
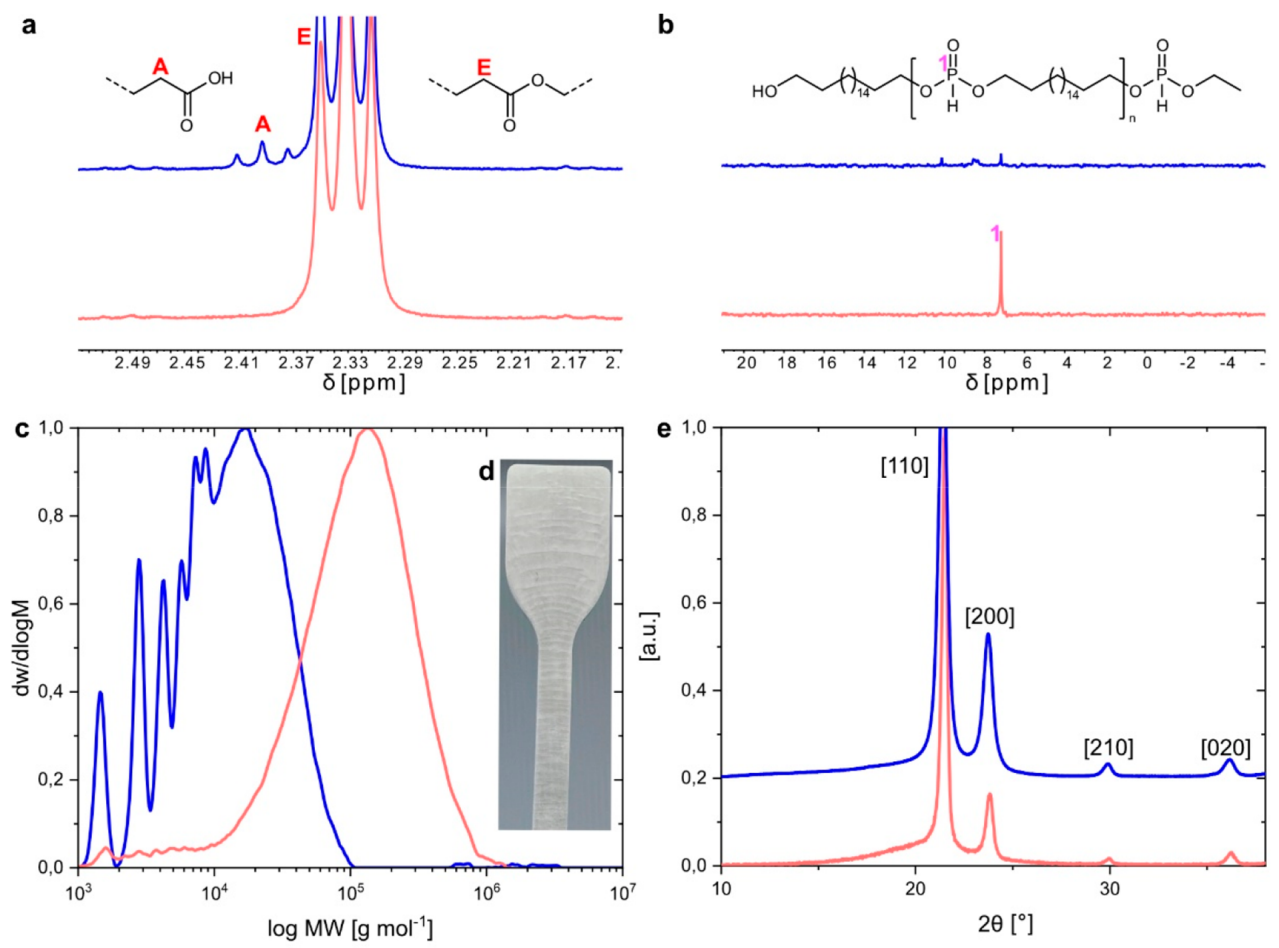
Hydrolytic degradation of polyethylene-like PE-18,18/PP-18 blends. (a) Details of ^1^H NMR spectra of PE-18,18 blended with 10 wt % PP-18 before (red) and after (blue) exposure to a phosphate buffered (pH 8) hydrolysis medium for 4 months. (b) Details of ^31^P NMR spectra of PE-18,18 blended with 10 wt % PP-18 before (red) and after (blue) exposure to a phosphate buffered (pH 8) hydrolysis medium for 4 months. (c) GPC traces of PE-18,18 blended with 10 wt % PP-18 before (red, *M*_n_ = 72 kg mol^–1^, *M*_w_ = 160 kg mol^–1^) and after (blue, *M*_n_ = 8 kg mol^–1^, *M*_w_ = 17 kg mol^–1^) exposure to a phosphate buffered (pH 8) hydrolysis medium for 4 months. (d) Photo of an embrittled tensile test specimen of PE-18,18 blended with 10 wt % PP-18 after 4 months immersion in 15 L of deionized water at 25 °C. (e) WAXS diffractograms of PE-18,18 blended with 10 wt % PP-18 before (red, χ = 65%) and after (blue, χ = 87%) exposure to a phosphate buffered (pH 8) hydrolysis medium for 4 months. Diffraction peaks correspond to the orthorhombic unit cell of polyethylene in all cases. Traces are shifted vertically for clarity. a.u., arbitrary units.

The hydrolytic degradability of the blends was investigated on injection molded specimens. Rectangular specimens (10 mm × 7 mm × 1 mm, approximately 85 mg) were exposed to neat water (which facilitated the observation of released acid from changes of the pH) and phosphate-buffered media (which more closely resembled natural conditions in terms of pH). NMR analysis of the specimens after 4 months of exposure to the phosphate-buffered media revealed that the hydrolytically labile poly(H-phosphonate) blend component hydrolyzed to the underlying long-chain aliphatic diol (i.e., C_18_ diol for PP-18 and C_26_ diol for PP-26) and phosphorous acid (Figure 4b, cf. Figures S72–S84 for additional ^1^H and ^31^P NMR analysis of hydrolyzed blends). Note that for NMR and GPC analysis the entire specimen was dissolved; that is, the results are representative of the entire sample volume as opposed to, e.g., only a surface layer. The formation of phosphorous acid was indicated by an acidification of the neat water hydrolysis media (pH 2.4 for PE-18,18/PP-26 (20 wt %) blend after 4 months). Due to its low solubility in water, the long-chain diol remained within the polymer matrix as evidenced by the corresponding resonances in the ^1^H NMR spectra of the analyzed blend samples. The exposure of the blend samples to the buffered media also lead to the evolution of a new proton resonance (δ = 2.39 ppm) (Figure 4a). This resonance can be assigned to the methylene unit in α position to a carboxylic acid functional group indicating the phosphorous acid catalyzed hydrolysis of the PE-18,18 main blend component. GPC analyses of the blend samples confirmed the reduction of the molar masses of the blends concomitant with the hydrolysis of the ester functional groups (Figure 4c, cf. Figures S87–S93 for additional GPC analysis of hydrolyzed blends). The number-average molar masses of the virgin blends were in the range of 50–70 kg mol^–1^ and decreased to 7–11 kg mol^–1^ upon exposure of the blend samples to the buffered media for 4 months. The absence of residual high molar mass fractions in the GPC chromatograms indicates that the abiotic hydrolysis of the blends proceeded throughout the bulk of the materials. This is opposed to a relatively slow surface hydrolysis mechanism expected for highly crystalline HDPE-like polymers^33^ such as neat PE-18,18 which proved to be stable even under acidic and basic conditions (cf. Figures S101–S104 for analysis of PE-18,18 exposed to hydrolysis media). Note that the final hydrolysis products of the blends, long-chain diols, and diacids, as well phosphorous acid, can be metabolized by microorganisms and are classified as nonhazardous to the environment.^34^

The amount and type of poly(H-phosphonate) blended into the PE-18,18 matrix as an acid-releasing degradation-enhancing component did not impact the degree of hydrolysis notably. A comparable reduction in molar mass was observed for all blends. Solely, the blend with the lowest poly(H-phosphonate) content of 0.5 wt %, the only blend stable under ambient conditions, hydrolyzed to a lesser extent within the 4 months investigated (*M*_n_(virgin) = 65 kg mol^–1^ vs *M*_n_(buffer) = 46 kg mol^–1^, cf. Figure S91). Also in this case, the chromatogram as a whole was shifted to lower molar masses, and no high molar mass fractions remained. In addition to a duration of 4 months, the degrees of hydrolysis of the PE-18,18/PP-26 blends were also investigated after 8 and 12 months of exposure to hydrolysis media. ^1^H and ^31^P NMR analyses revealed that the hydrolysis of the labile poly(H-phosphonate) component and therefore the release of phosphorous acid mainly proceeded within the initial 4 months of exposure to aqueous media (cf. Figures S72–S79 for ^1^H and ^31^P NMR analyses of hydrolyzed blends). The hydrolysis of the PE-18,18 main component slows down when the poly(H-phosphonate) has been hydrolyzed and the phosphorous acid released from the blend (cf. Figure S86 for the decrease of *M*_n_ of the PE-18,18 blend component over time). WAXS performed on the degraded blend samples showed an increase in crystallinity in the range of 10–20 pp. after 4 months of exposure to buffered hydrolysis media (Figure 4e, cf. Figures S94–S100 for additional WAXS analysis of hydrolyzed blends). This finding, indicative of a preferential hydrolysis of the amorphous regions of the polymer and chain cleavage-induced crystallization,^35^ is also assumed to contribute to the aforementioned deceleration of the hydrolysis rate. Note that stabilizers typically added to polyolefins for longer-term stabilization and stability during processing (like hindered phenols and organophosphites) serve as antioxidants but are not expected to significantly impact hydrolysis reactions.

**Figure 5:**
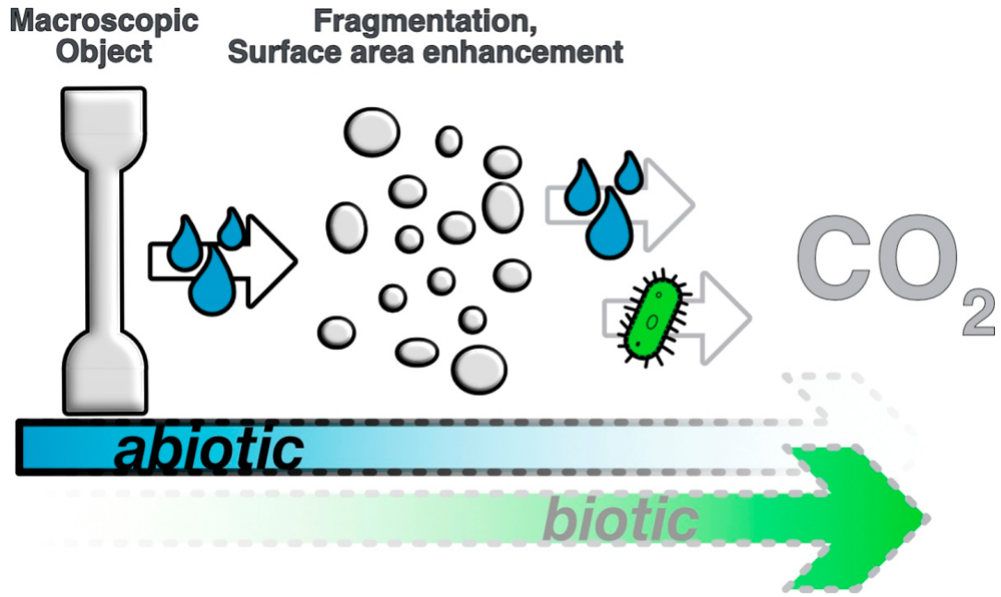
Observed abiotic degradation resulting in disintegration and surface area enhancement (left) and anticipated further breakdown and eventual mineralization (right).

To quantify the embrittlement of the low molar mass PE-18,18 resulting from the hydrolysis of the blends, two tensile test specimens (ISO 527-2, type 5A) of each blend of PE-18,18 with 0.5, 2, and 10 wt % PP-18 were immersed in ca. 15 L of deionized water for 16 weeks at 25 °C. The 10 and 2 wt % PP-18 containing blends completely embrittled impeding tensile testing (Figure 4d, cf. Figure S108 for photograph of embrittled tensile test specimens). The stress–strain curves obtained for the 0.5 wt % PP-18 containing blend revealed a significant deterioration in ductility (ε_tb_(virgin) ≈ 460% vs ε_tb_(water exposure) ≈ 90%, cf. Figure S109). Notably, the ductility of reference specimens of neat PE-18,18 remained unaltered upon exposure to the same conditions (ε_tb_(virgin) ≈ 540% vs ε_tb_(water exposure) ≈ 540%, cf. Figure S106). The results suggest the formation of low molar mass PE-18,18 fragments from the blends under the influence of water (Figure 5).

## Conclusions

Renewable, long-chain aliphatic polyesters with HDPE-like material properties are chemically recyclable in a closed loop, which enables a circular economy. However, when unintentionally released into the environment, additional abiotic degradability is desired as a universal backstop to plastic accumulation. We show that melt blending with hydrolyzable, acid-releasing poly(H-phosphonate)s endows HDPE-like polyesters with hydrolytic degradability. The resulting polymer blends retain HDPE-like materials properties, and at the same time, despite their mainly hydrocarbon and crystalline nature, they are amenable to abiotic degradation in an aqueous environment. Concomitant with hydrolytic molar mass reduction, the materials embrittle and fragment (Figure 5, left). The resulting increase in surface area is anticipated to enable further cleavage of the remaining ester bonds and eventual mineralization via abiotic and biotic pathways (Figure 5, right). The fundamental findings reported here suggest that the concept can allow for adapting hydrolytic degradability for individual applications to enable sufficient stability during product service life but prevent long-term persistency. In addition, future long-term studies are required to elucidate the final fate and the time scale of degradation of these promising materials in the environment.

## Supporting Information Available

Supplementary methods and data, including additional polymer and blend characterization (NMR spectra, GPC chromatograms, DSC traces, WAXS diffractograms, stress–strain curves, surface tension, TGA traces), details for filament fabrication, 3D printing parameters and characterization of 3D printed blend specimens, details of hydrolysis experiments and additional characterization of specimens exposed to hydrolysis media (optical impression, SEM images, weight analysis, NMR spectra with molar mass determination via end group analysis, GPC chromatograms, WAXS diffractograms), additional characterization of PE-18,18 exposed to hydrolysis media (NMR spectra, GPC chromatograms, weight analysis, optical impression, WAXS diffractograms), additional data for aqueous exposure of test bar specimens experiments, additional data for stability of test bar specimens in air under ambient conditions experiments, and additional monomer characterization. Supplementary tables, including tensile properties, molar masses, crystallinity and weights of virgin blends and reference materials in comparison to materials after exposure to different media. (PDF)

## Abbreviations

HDPE: high density polyethylene
PE-18,18: polyester-18,18
PP-18: poly(H-phosphonate)-18
PP-26: poly(H-phosphonate)-26
