# Supplementary material for: Polyethylene-Like Blends Amenable to Abiotic Hydrolytic Degradation
Source: ACS Sustain Chem Eng. 2023 Mar 13;11(12):4523–30. doi: 10.1021/acssuschemeng.2c07537 (PMC10052336; doi:10.1021/acssuschemeng.2c07537)
Supplement: Supplementary file 1 — sc2c07537_si_001.pdf [file sc2c07537_si_001.pdf]

Supplementary Information

Polyethylene-Like Blends Amenable to Abiotic Hydrolytic Degradation

Marcel Eck, Léa Bernabeu, and Stefan Mecking*

Chair of Chemical Materials Science, University of Konstanz,
Department of Chemistry, Universitätsstraße 10, 78457 Konstanz, Germany.

* stefan.mecking@uni-konstanz.de, Fax: +49 7531 885152
Tel.: +49 7531 882593

Number of pages: 81

Number of figures: 122

Number of tables: 21

Table of Contents

S1. Supplementary Methods & Data 4

Additional characterization data for Poly(H-phosphonate)-26........ 4

Additional characterization data for Poly(H-phosphonate)-18........ 8

Investigation of the miscibility of PE-18.18 and PP-26 *via* DSC 12

Additional characterization data for PE-18.18 with 2 wt% PP-26........13

Additional characterization data for PE-18.18 with 5 wt% PP-26........15

Additional characterization data for PE-18.18 with 10 wt% PP-26........17

Additional characterization data for PE-18.18 with 20 wt% PP-26........19

Tensile properties of PE-18.18/PP-26 blends........21

Surface free energies of PE-18.18/PP-26 blends........21

Additional characterization data for PE-18.18 with 0.5 wt% PP-18........22

Additional characterization data for PE-18.18 with 2 wt% PP-18........24

Additional characterization data for PE-18.18 with 10 wt% PP-18........26

Tensile properties of PE-18.18/PP-18 blends........28

Filament extrusion process 29

Additional characterization of PE-18.18/PP-18 (0.5 wt%) blend filament........29

3D printing parameters 31

Additional characterization of PE-18.18/PP-18 (0.5 wt%) blend after 3D printing........36

Experimental setup of the hydrolysis experiments 37

Optical impression of the PE-18.18/PP-26 blend specimens exposed to hydrolysis media 38

Optical impression of the PE-18.18/PP-18 blend specimens exposed to hydrolysis media 39

SEM images of the PE-18.18/PP-26 blend specimens exposed to hydrolysis media........40

Weight change of the PE-18.18/PP-26 blend specimens exposed to hydrolysis media........41

Assignment of the ^{1}H NMR resonances of the blends exposed to hydrolysis media 43

NMR analysis of the PE-18.18/PP-26 blends exposed to hydrolysis media 45

NMR analysis of the PE-18.18/PP-18 blends exposed to hydrolysis media 49

Estimation of the molecular weights of the PE-18.18 component of PE-18.18/PP-18/26 blends exposed to hydrolysis media by ^{1}H NMR........52

Determination of the molecular weights of PE-18.18/PP-26 blends exposed to hydrolysis media by GPC 54

Determination of the molecular weights of PE-18.18/PP-18 blends exposed to hydrolysis media by GPC 56

WAXS analysis of the PE-18.18/PP-26 blend specimens exposed to hydrolysis media........58

WAXS analysis of the PE-18.18/PP-18 blend specimens exposed to hydrolysis media........60

Analysis of injection molded PE-18.18 specimens exposed to hydrolysis media........62

Additional data for aqueous exposure of test bar specimens 64
Additional data for stability of test bar specimens in air under ambient conditions....66
Additional characterization data for 1,18-octadecanediol 70
Additional characterization data for 1,26-hexacosanediol 71
Additional characterization data for PE-18.18 72
S2. Supplementary Tables 75
Tensile properties of blends and of reference materials 75
Molecular weights of PE-18.18/PP-26 blends 77
Molecular weights of PE-18.18/PP-18 blends 78
Crystallinity of blends....78
Weight changes of the blend samples exposed to aqueous media 79
References 81

S1. Supplementary Methods & Data

Additional characterization data for Poly(H-phosphonate)-26

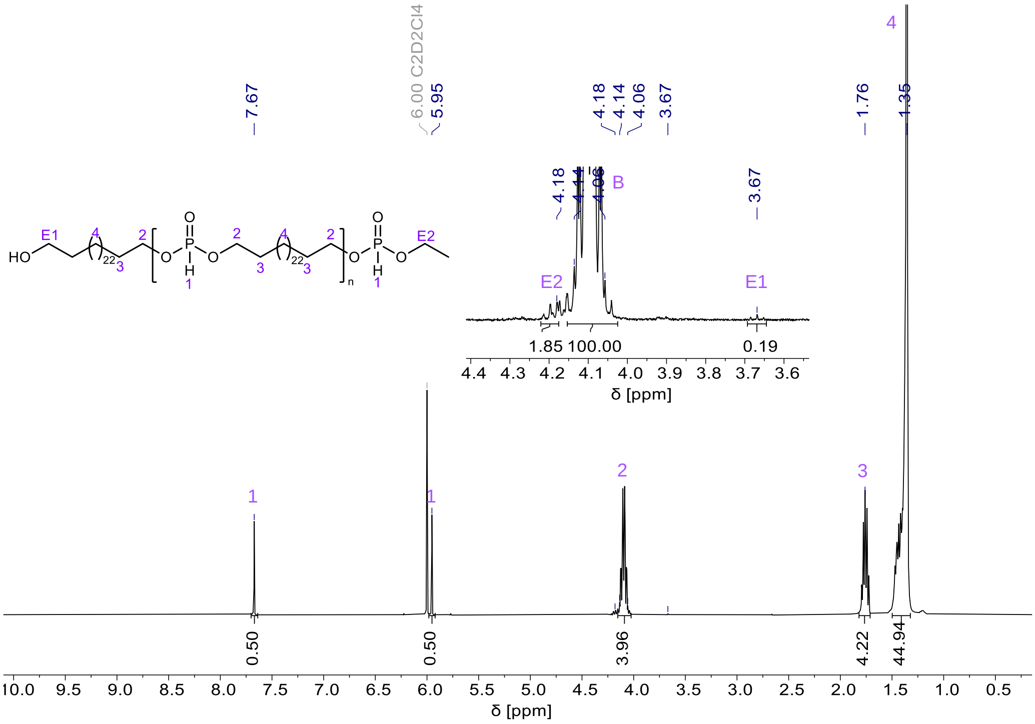

Figure S 1. ^{1}H NMR spectrum (400 MHz, $C_2D_2Cl_4$, 383 K) of PP-26. The enlarged excerpt shows the integrals of the backbone "B" and the hydroxy "E1" and ethyl ester "E2" end group resonances used for the determination of the number average molecular weight M_n by **Equations S 1** and **S 2**. The number average molecular M_n weight obtained amounts to 12 kg/mol.

Equation S 1.

$$DP_n = \frac{\int B}{\int E1 + \int E2} + 1$$

Equation S 2.

$$M_n = DP_n * 445\, g/mol$$

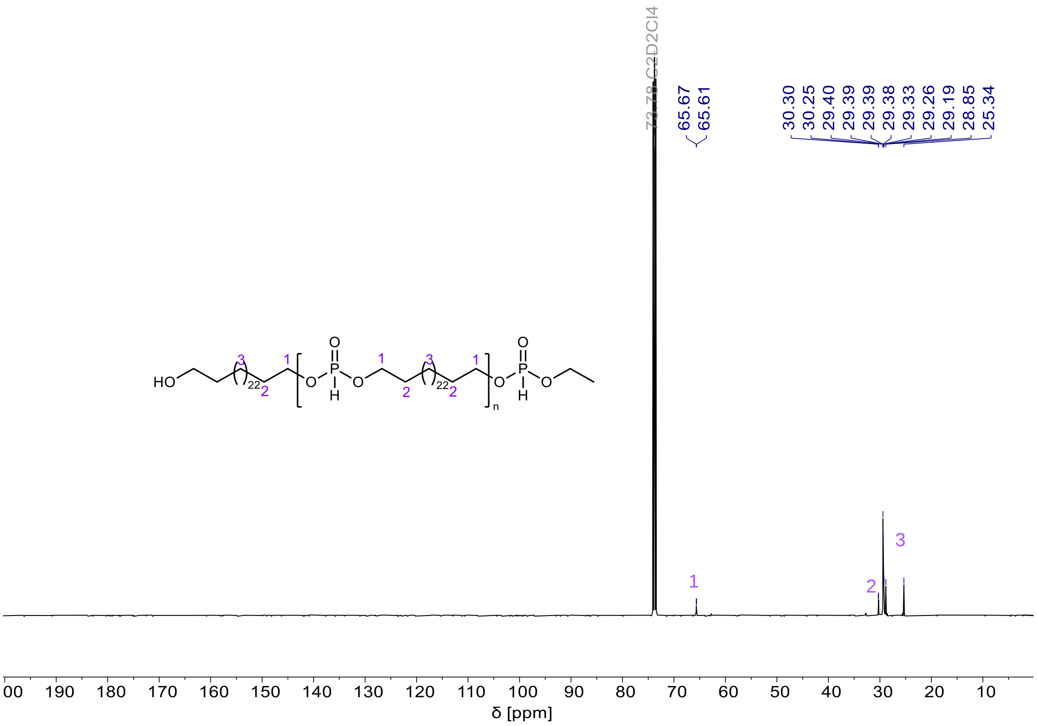

Figure S 2. $^{13}C\{^{1}H\}$ NMR spectrum (101 MHz, $C_2D_2Cl_4$, 383 K) of PP-26.

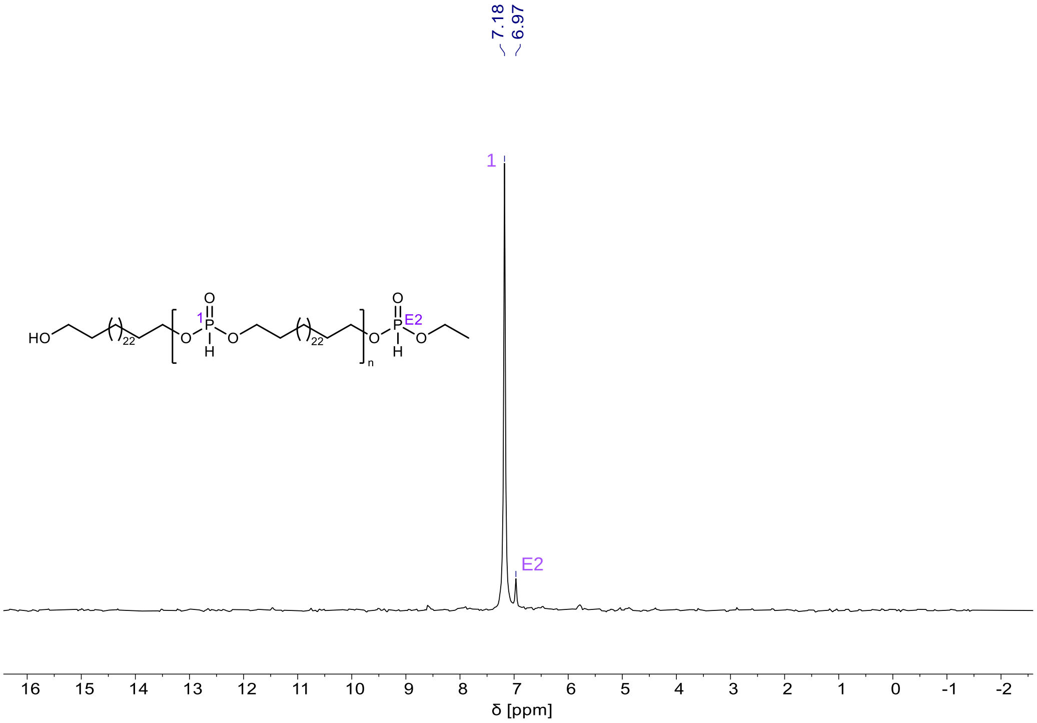

Figure S 3. $^{31}P\{^{1}H\}$ NMR spectrum (162 MHz, $C_2D_2Cl_4$, 383 K) of PP-26.

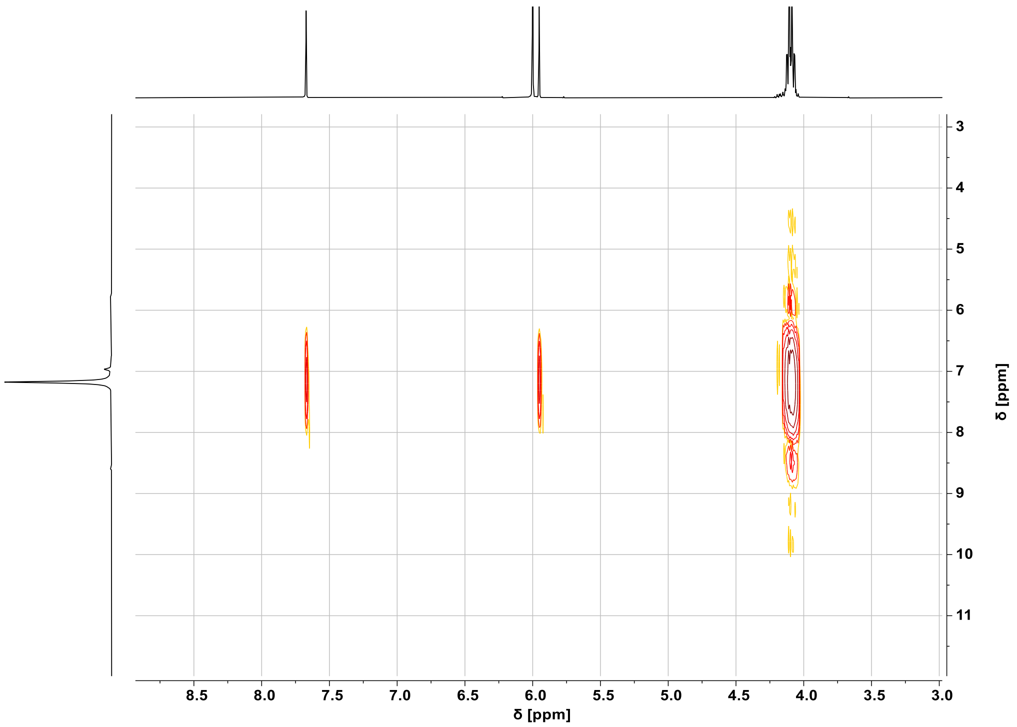

Figure S 4. ^{1}H-^{31}P HMBC NMR spectrum (400 MHz/162 MHz, $C_2D_2Cl_4$, 383 K) of PP-26.

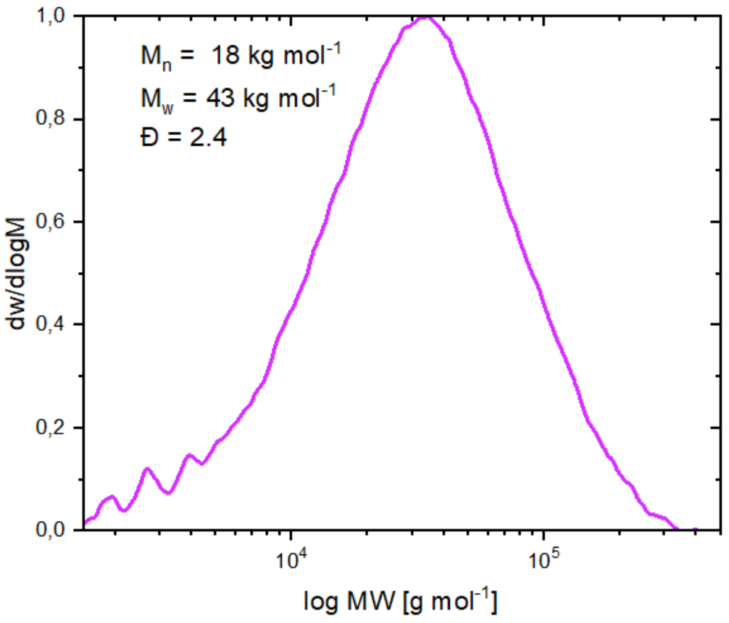

Figure S 5. GPC trace of PP-26.

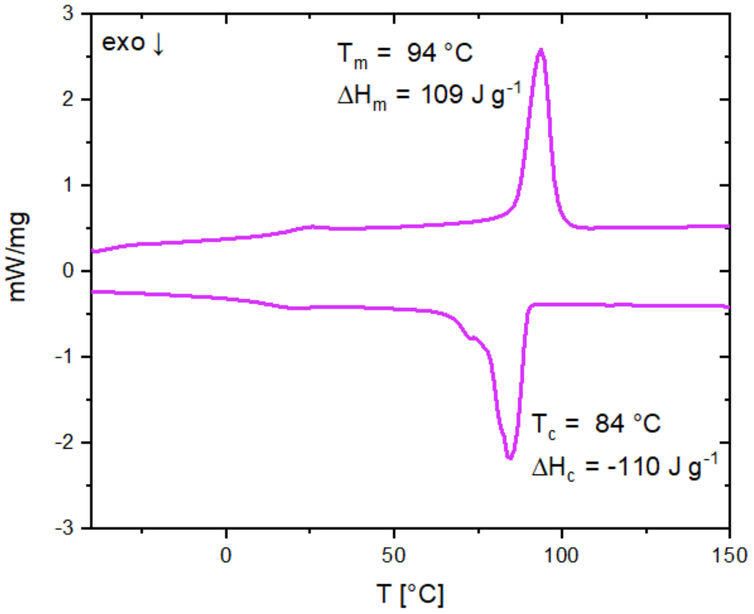

Figure S 6. DSC trace of PP-26.

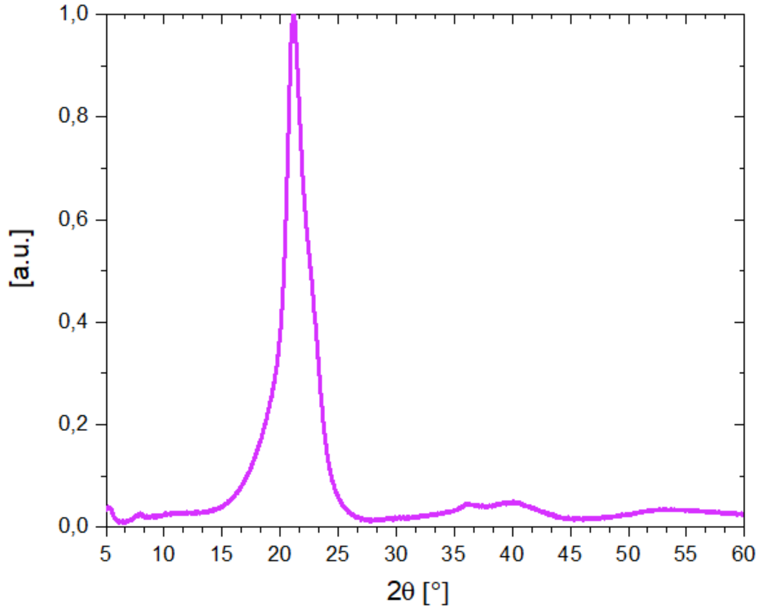

Figure S 7. WAXS diffractogram of PP-26.

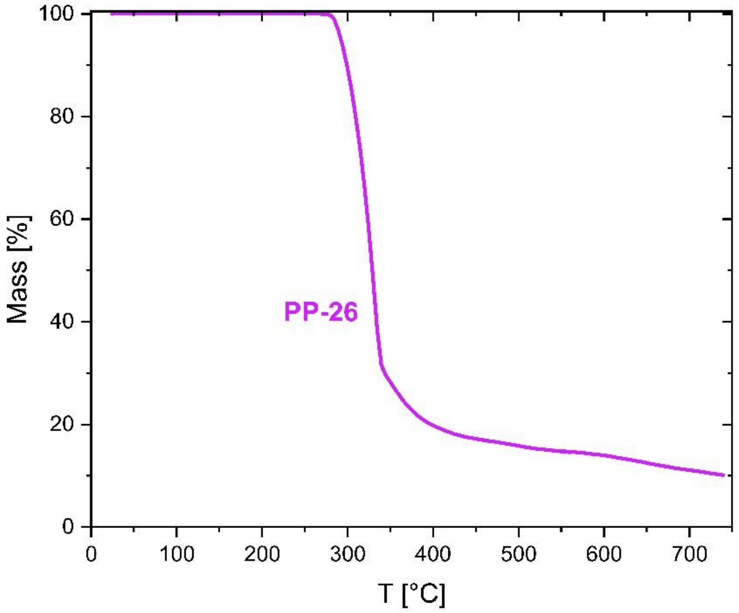

Figure S 8. TGA trace of PP-26. 5 % weight loss was observed at T ≈ 294 °C.

Additional characterization data for Poly(H-phosphonate)-18

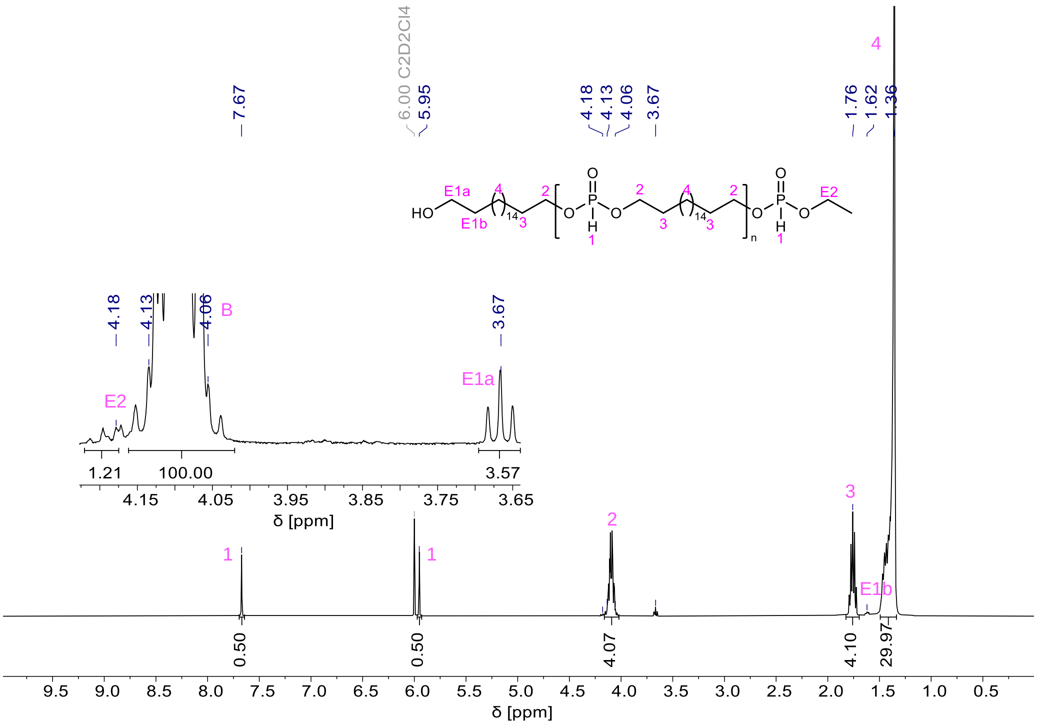

Figure S 9. ^1H NMR spectrum (400 MHz, $C_2D_2Cl_4$, 383 K) of PP-18. The enlarged excerpt shows the integrals of the backbone "B" and the hydroxy "E1" and ethylester "E2" end group resonances used for the determination of the number average molecular weight M_n by **Equations S 3** and **S 4.** The number average molecular M_n weight obtained amounts to 6 kg/mol.

Equation S 3.

$$DP_n = \frac{\int B}{\int E1 + \int E2} + 1$$

Equation S 4.

$$M_n = DP_n * 332\, g/mol$$

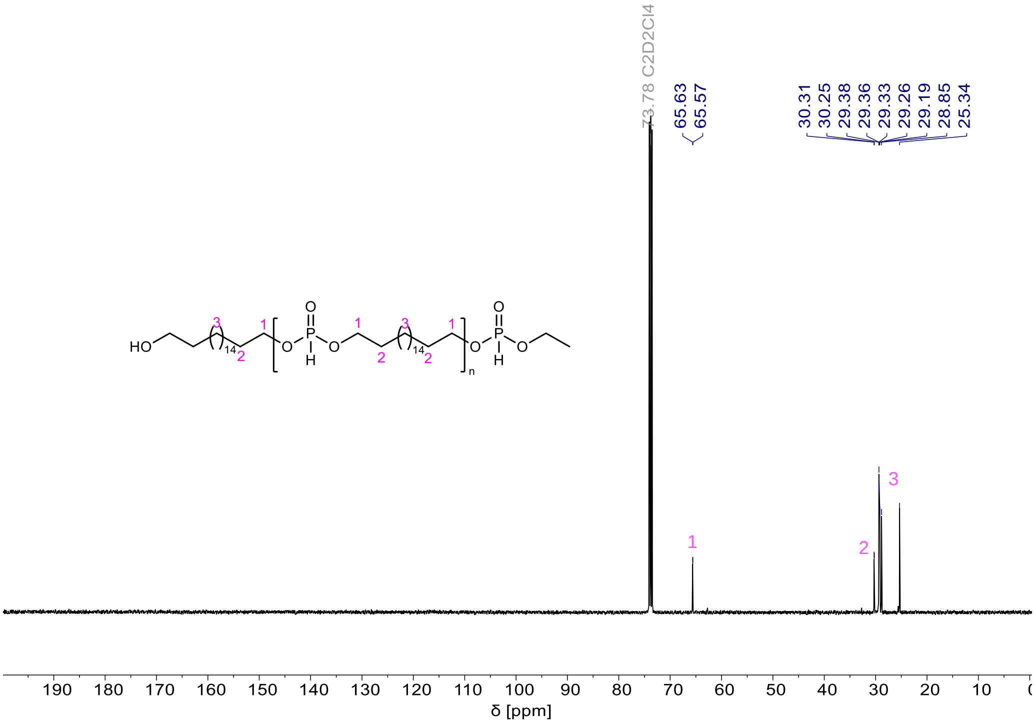

Figure S 10. $^{13}C\{^{1}H\}$ NMR spectrum (101 MHz, $C_2D_2Cl_4$, 383 K) of PP-18.

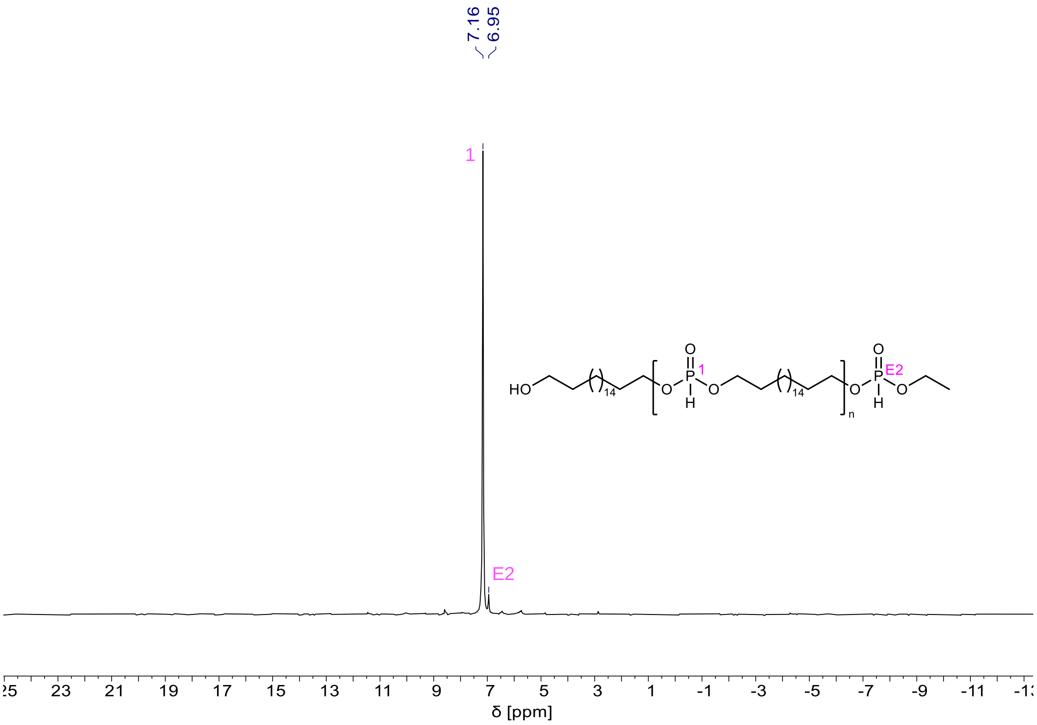

Figure S 11. $^{31}P\{^{1}H\}$ NMR spectrum (162 MHz, $C_2D_2Cl_4$, 383 K) of PP-18.

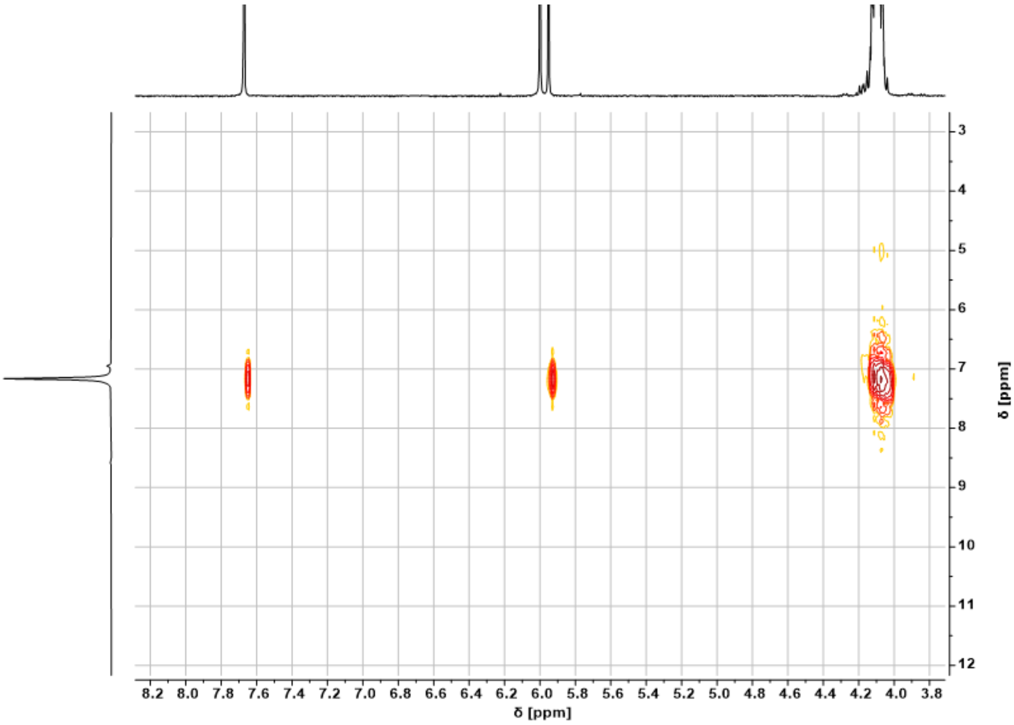

Figure S 12. ^{1}H-^{31}P HMBC NMR spectrum (400 MHz/162 MHz, $C_2D_2Cl_4$, 383 K) of PP-18.

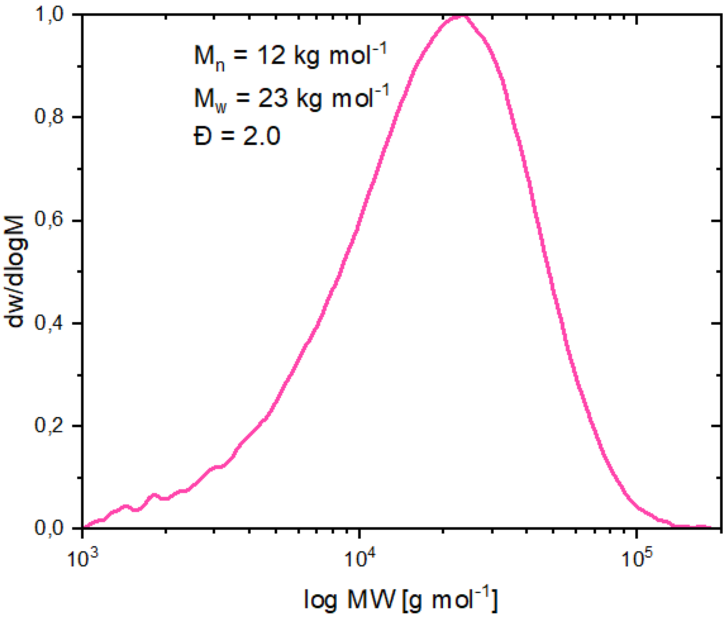

Figure S 13. GPC trace of PP-18.

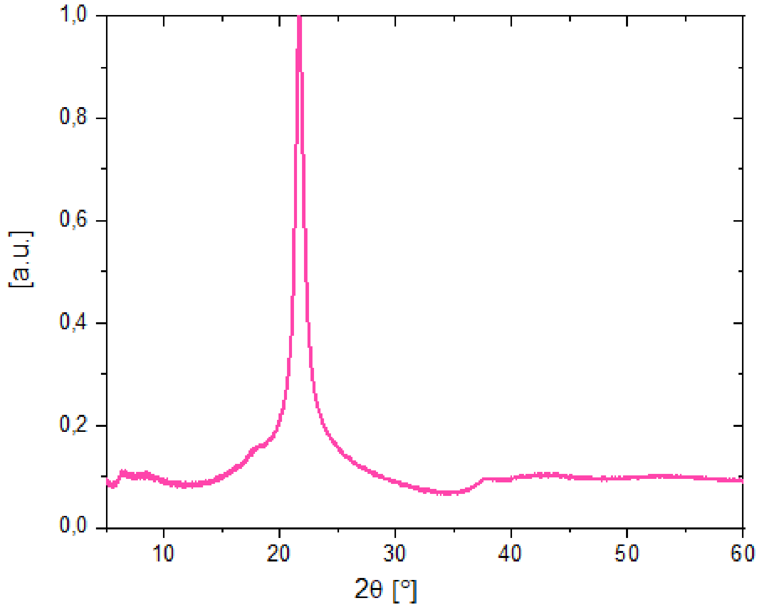

Figure S 14. WAXS diffractogram of PP-18.

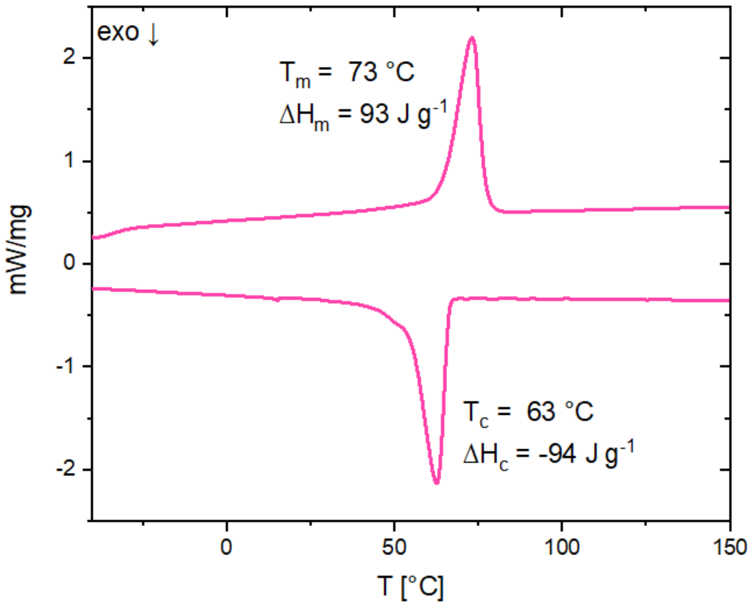

Figure S 15. DSC trace of PP-18.

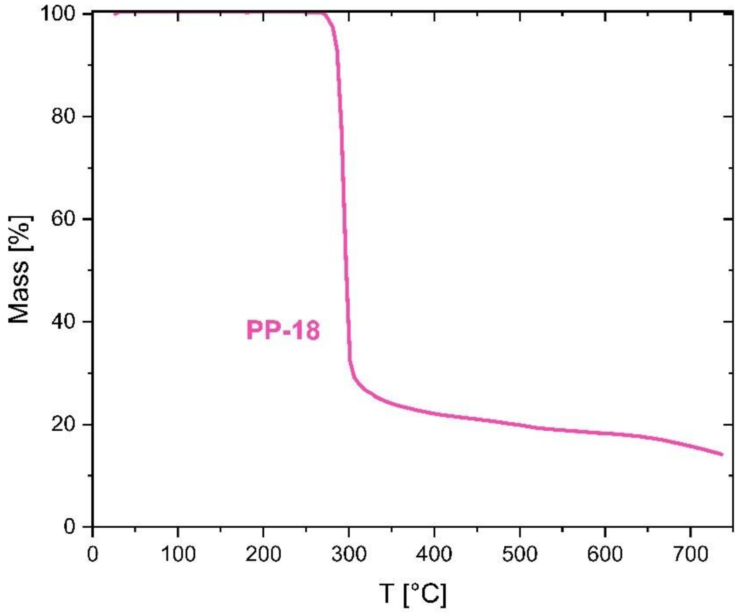

Figure S 16. TGA trace of PP-18. 5 % weight loss was observed at T ≈ 286 °C.

Investigation of the miscibility of PE-18.18 and PP-26 *via* DSC

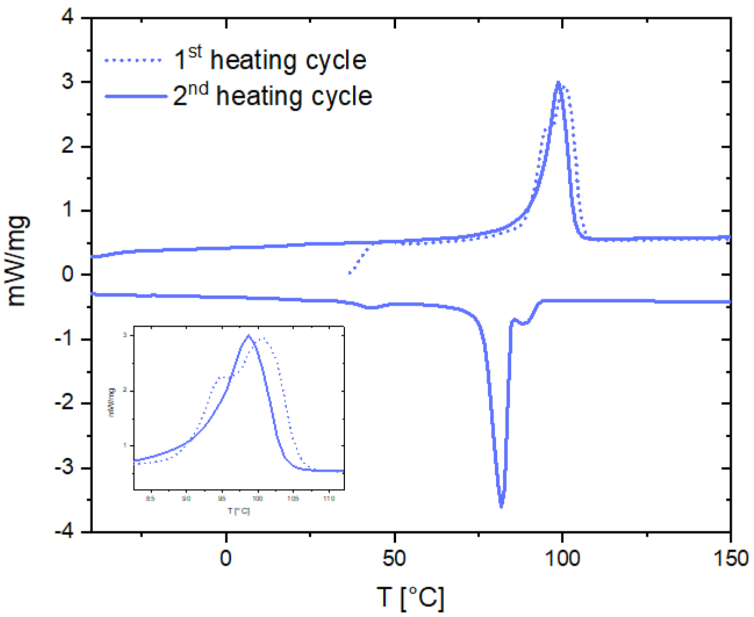

Figure S 17. PE-18.18 (80 wt%) and PP-26 (20 wt%) were premixed in a DSC pan. The DSC trace of the first heating cycle shows two melting points (T_{m1} = 95 °C and T_{m2} = 100 °C, shown in the enlarged excerpt). The DSC trace of second heating cycle exhibits a single melting point (T_m = 99 °C).

Single melting transitions observed in the DSC measurements of the compounded PE-18.18/PP-26 and PE-18.18/PP-18 blends indicate cocrystallization of the two long-chain aliphatic polymers. WAXS measurements conducted on injection moulded blend samples which yield an orthorhombic solid-state structure and a HDPE-like crystallinity of approx. 70 % support this assumption.

Additional characterization data for PE-18.18 with 2 wt% PP-26

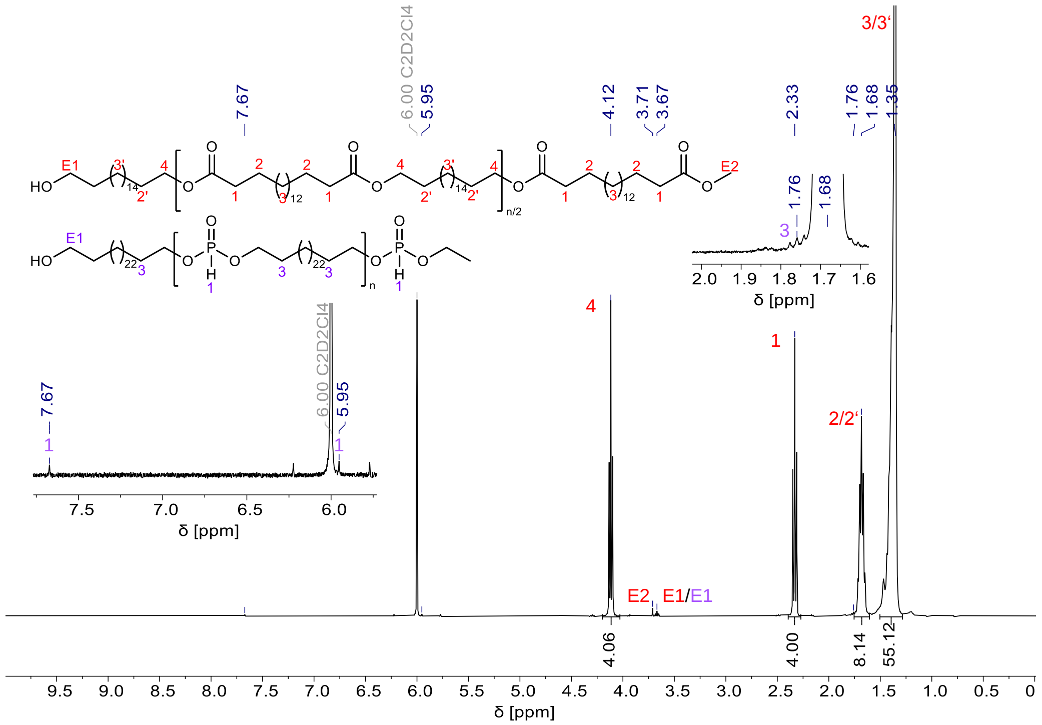

Figure S 18. ^{1}H NMR spectrum (400 MHz, $C_2D_2Cl_4$, 383 K) of PE-18.18 blended with 2 wt% PP-26. The enlarged details show the resonances stemming from the PP-26 blend component.

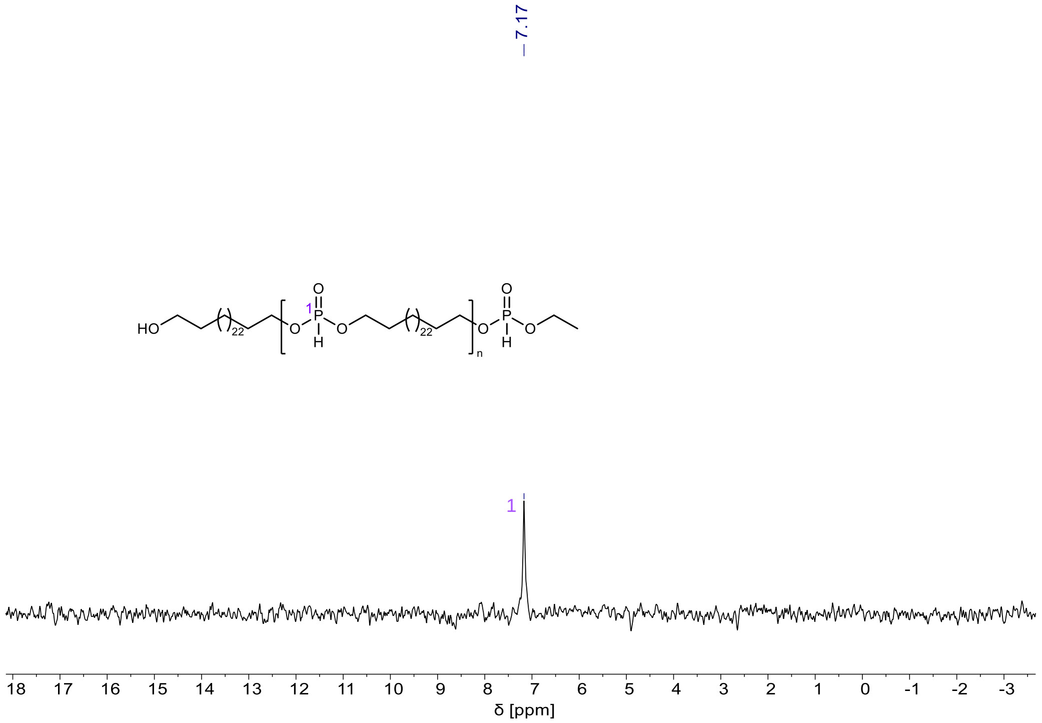

Figure S 19. $^{31}P\{^{1}H\}$ NMR spectrum (162 MHz, $C_2D_2Cl_4$, 383 K) of PE-18.18 blended with 2 wt% PP-26.

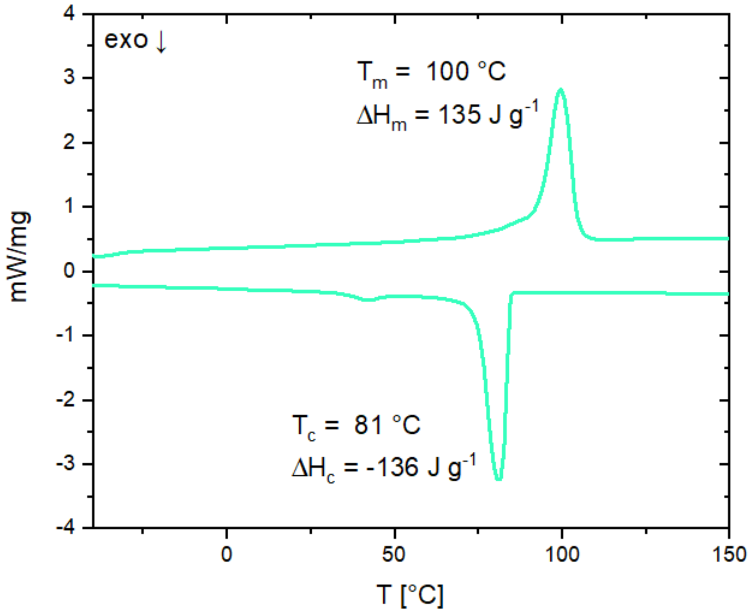

Figure S 20. DSC trace of PE-18.18 blended with 2 wt% PP-26.

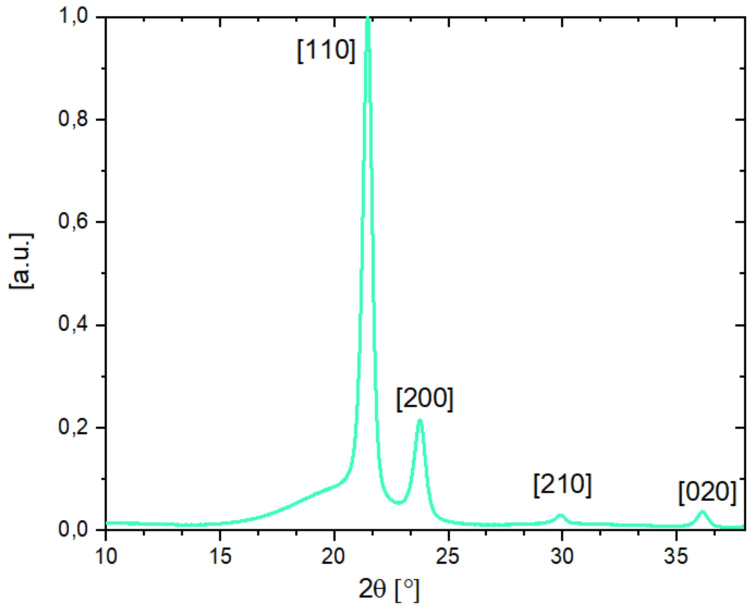

Figure S 21. WAXS diffractogram of PE-18.18 blended with 2 wt% PP-26. The crystallinity determined by deconvolution of the crystalline reflexes and the amorphous halo amounts to 69 %.

Additional characterization data for PE-18.18 with 5 wt% PP-26

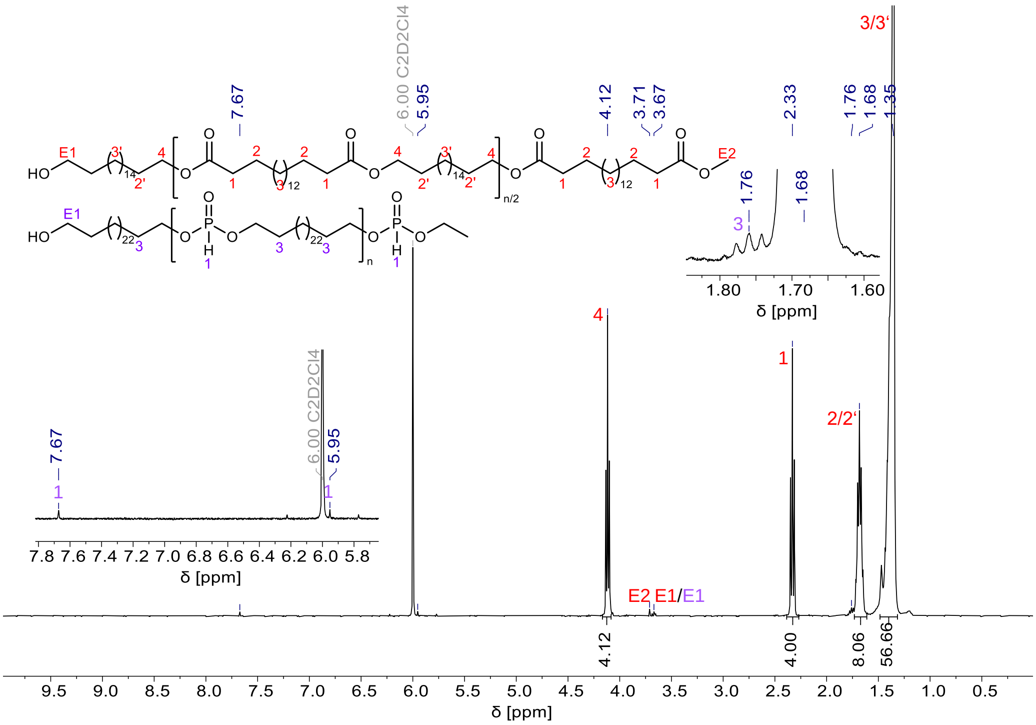

Figure S 22. ^{1}H NMR spectrum (400 MHz, $C_2D_2Cl_4$, 383 K) of PE-18.18 blended with 5 wt% PP-26. The enlarged excerpts show the resonances stemming from the PP-26 blend component.

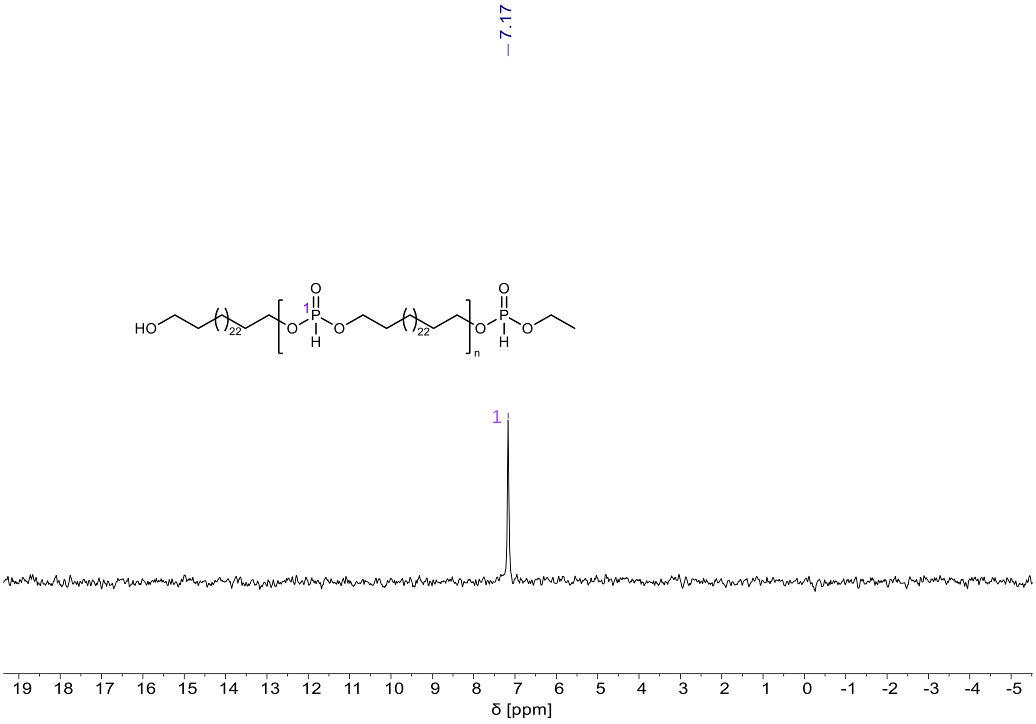

Figure S 23. $^{31}P\{^{1}H\}$ NMR spectrum (162 MHz, $C_2D_2Cl_4$, 383 K) of PE-18.18 blended with 5 wt% PP-26.

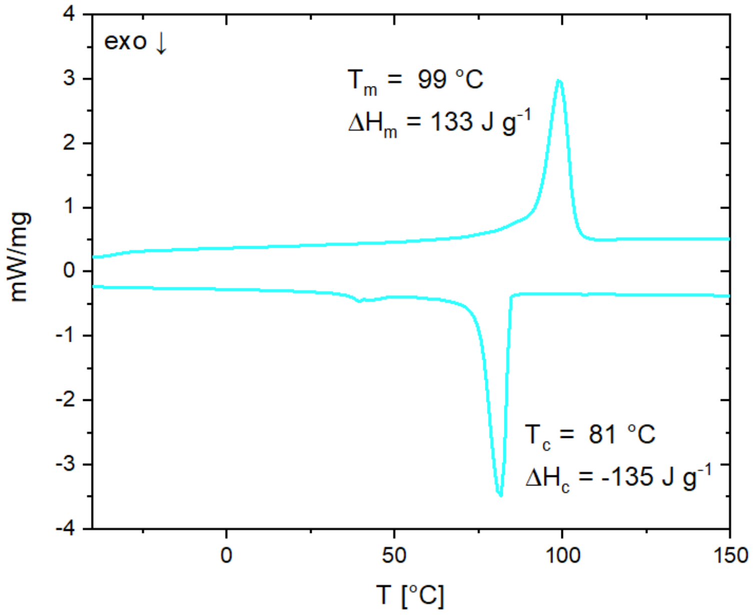

Figure S 24. DSC trace of PE-18.18 blended with 5 wt% PP-26.

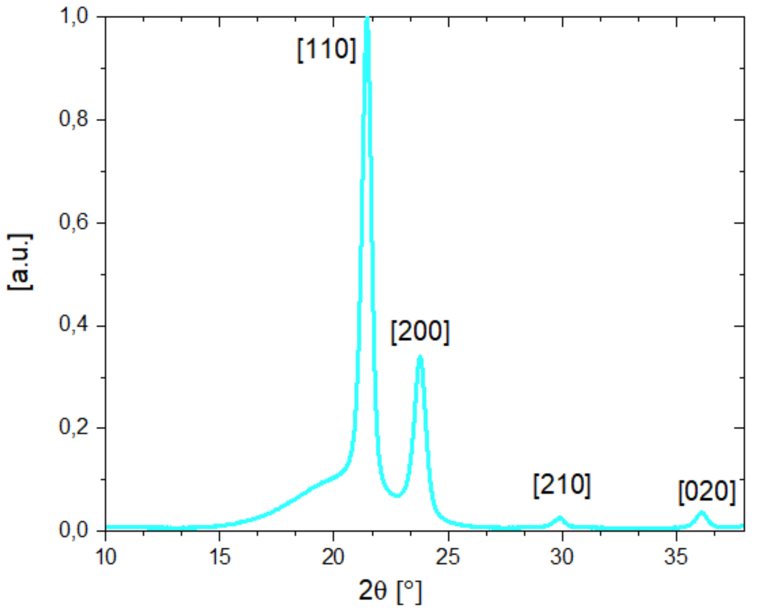

Figure S 25. WAXS diffractogram of PE-18.18 blended with 5 wt% PP-26. The crystallinity determined by deconvolution of the crystalline reflexes and the amorphous halo amounts to 69 %.

Additional characterization data for PE-18.18 with 10 wt% PP-26

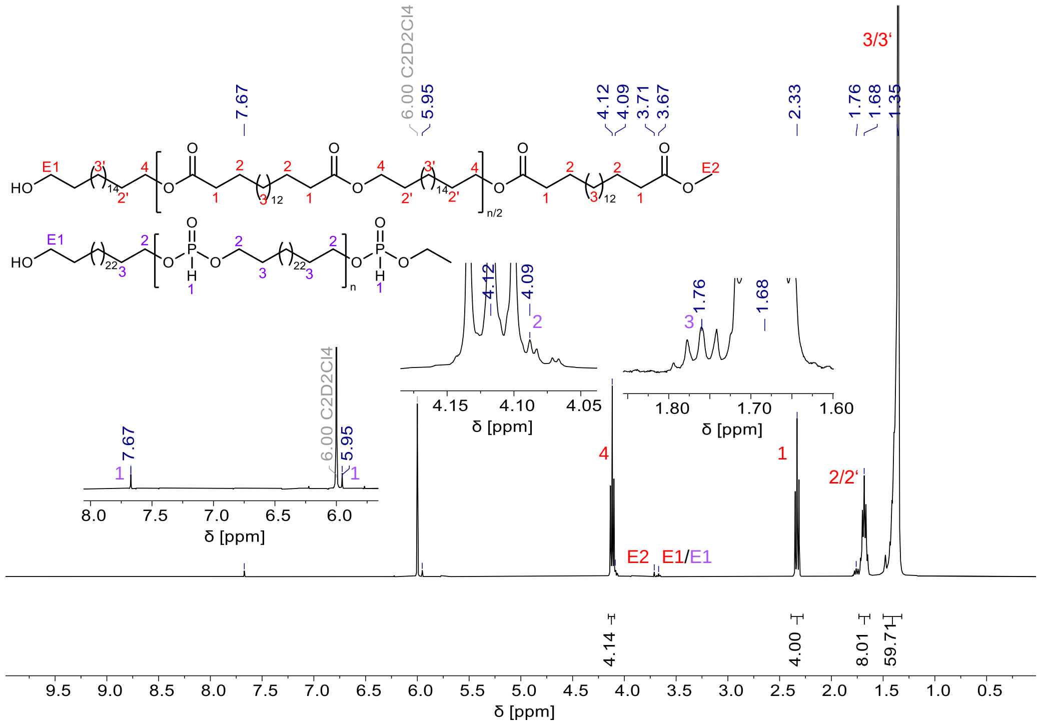

Figure S 26. ^{1}H NMR spectrum (400 MHz, $C_2D_2Cl_4$, 383 K) of PE-18.18 blended with 10 wt% PP-26. The enlarged excerpts show the resonances stemming from the PP-26 blend component.

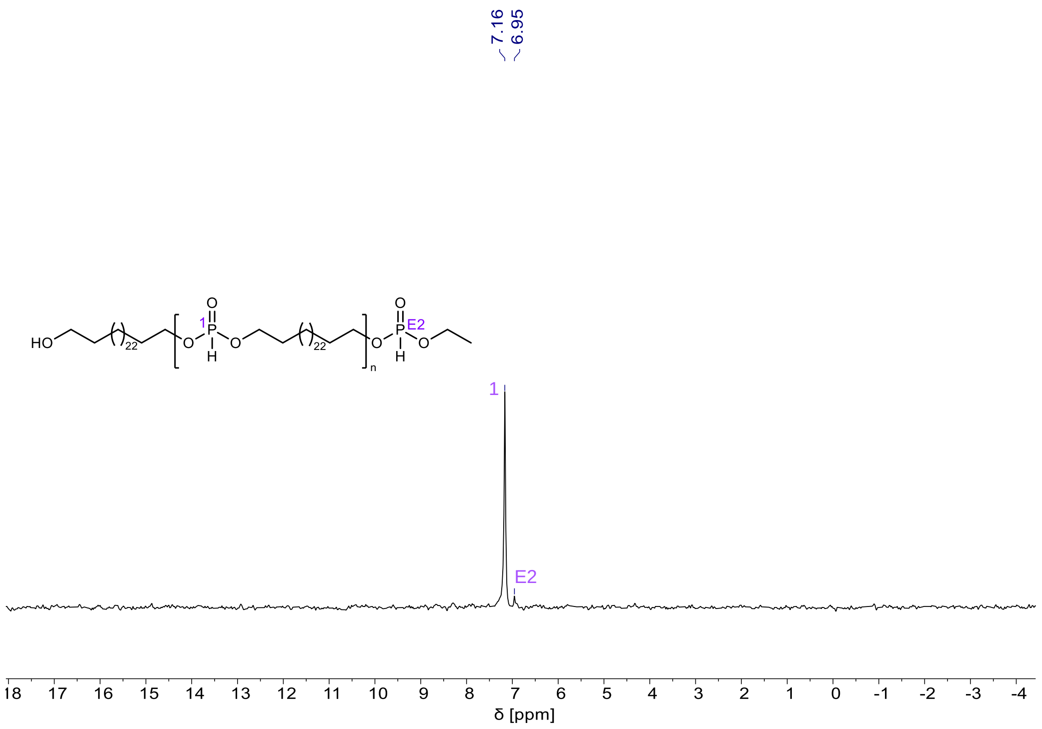

Figure S 27. $^{31}P\{^{1}H\}$ NMR spectrum (162 MHz, $C_2D_2Cl_4$, 383 K) of PE-18.18 blended with 10 wt% PP-26.

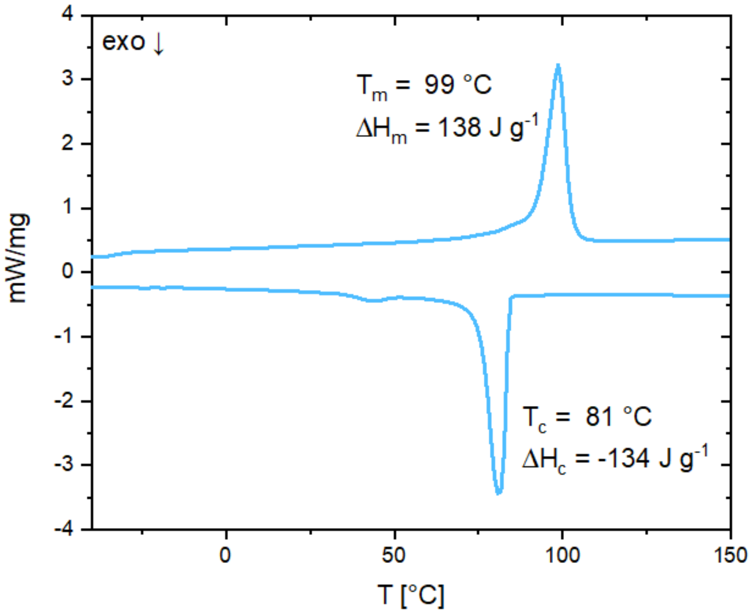

Figure S 28. DSC trace of PE-18.18 blended with 10 wt% PP-26.

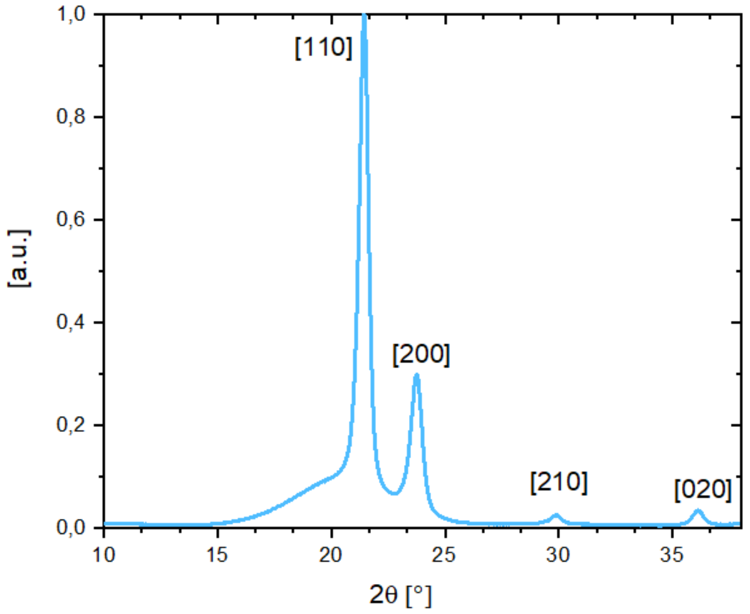

Figure S 29. WAXS diffractogram of PE-18.18 blended with 10 wt% PP-26. The crystallinity determined by deconvolution of the crystalline reflexes and the amorphous halo amounts to 72 %.

Additional characterization data for PE-18.18 with 20 wt% PP-26

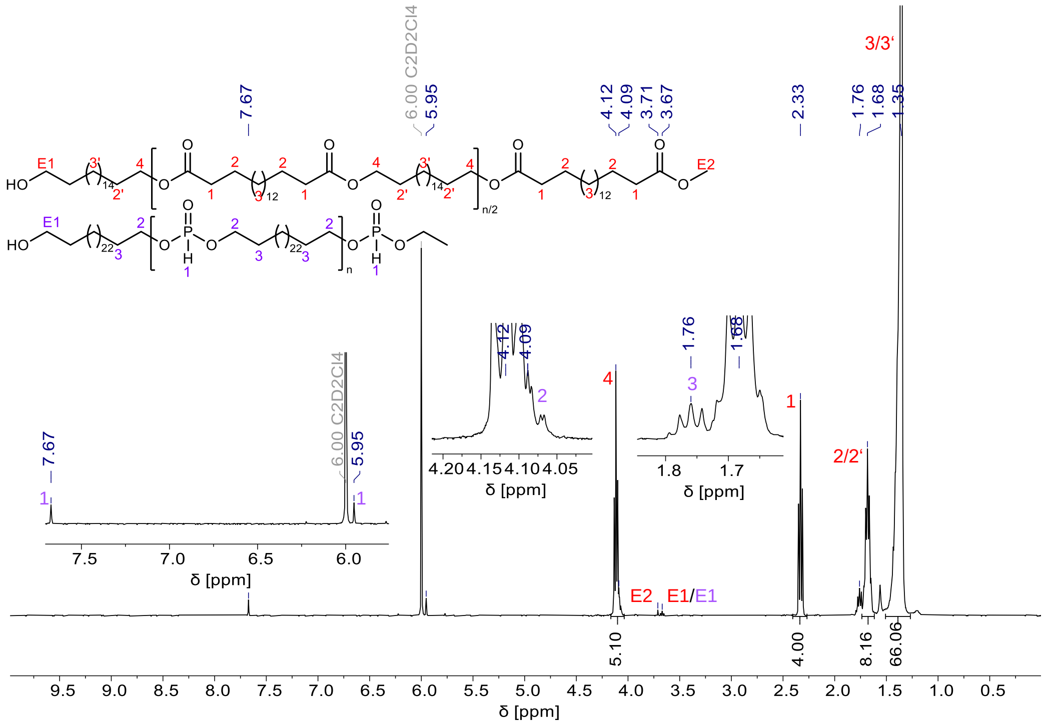

Figure S 30. ^{1}H NMR spectrum (400 MHz, $C_2D_2Cl_4$, 383 K) of PE-18.18 blended with 20 wt% PP-26. The enlarged excerpts show the resonances stemming from the PP-26 blend component.

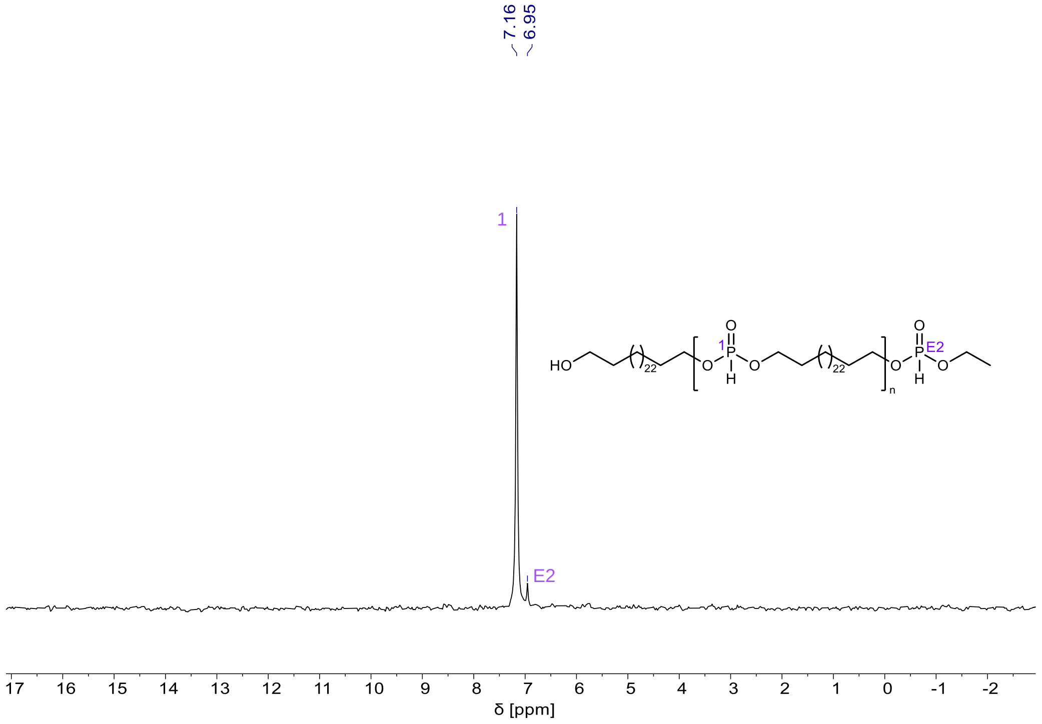

Figure S 31. $^{31}P\{^{1}H\}$ NMR spectrum (162 MHz, $C_2D_2Cl_4$, 383 K) of PE-18.18 blended with 20 wt% PP-26.

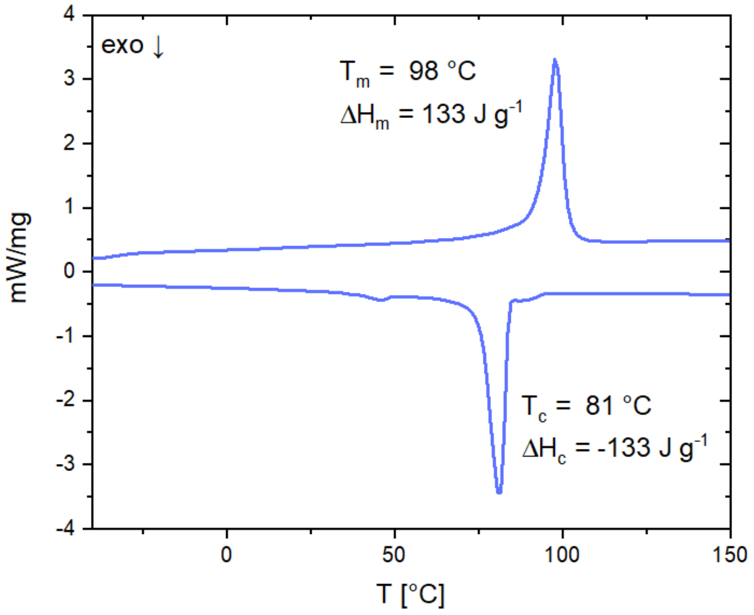

Figure S 32. DSC trace of PE-18.18 blended with 20 wt% PP-26.

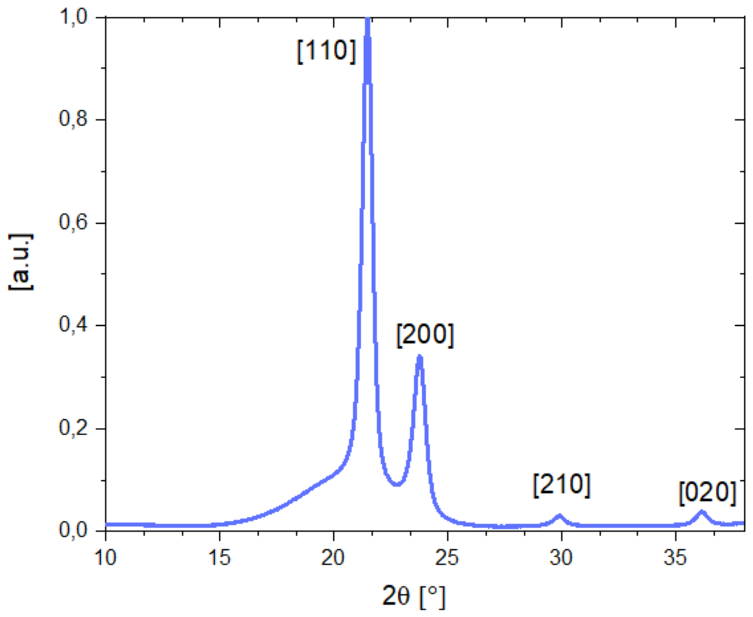

Figure S 33. WAXS diffractogram of PE-18.18 blended with 20 wt% PP-26. The crystallinity determined by deconvolution of the crystalline reflexes and the amorphous halo amounts to 70 %.

Tensile properties of PE-18.18/PP-26 blends

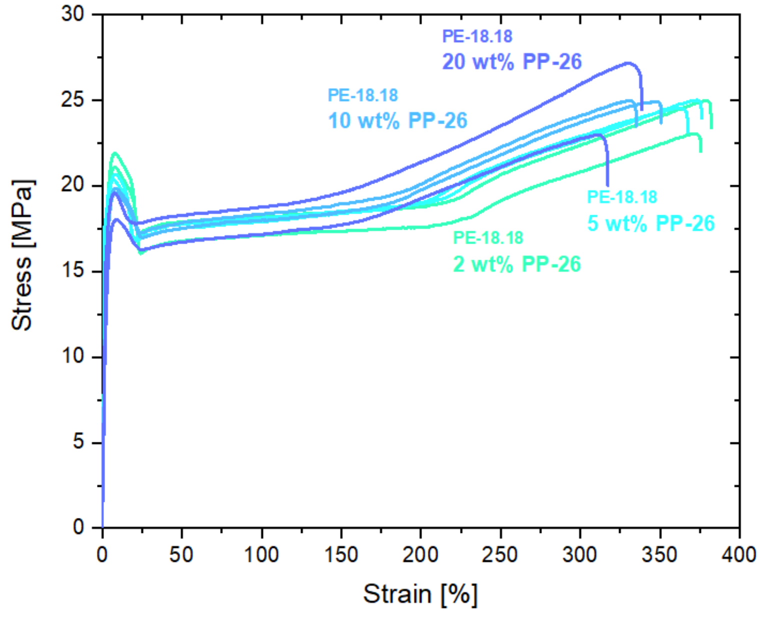

Figure S 34. Stress-strain curves of PE-18.18/PP-26 blends.

Surface free energies of PE-18.18/PP-26 blends

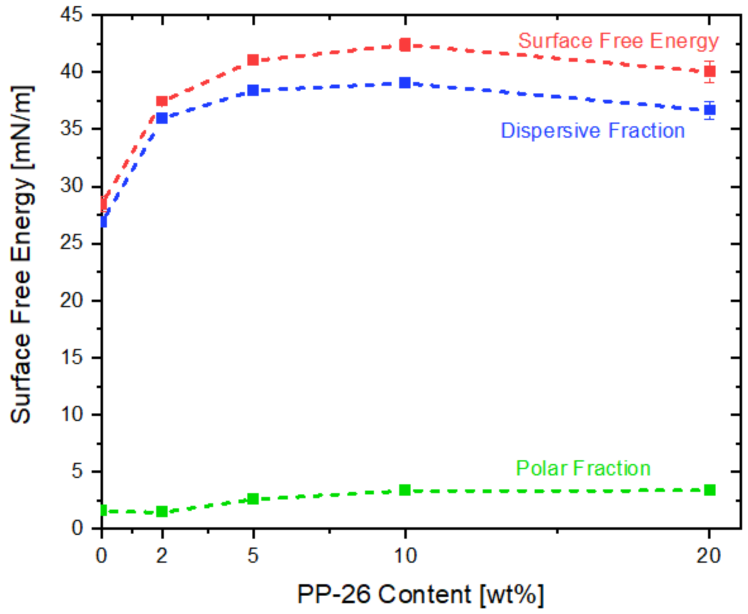

Figure S 35. Surface free energies of PE-18.18/PP-26 blends. The increasing surface free energies indicate an enhanced wettability of the injection molded specimens with an increasing PP-26 blend content. Connecting lines are a guide to the eye.

Additional characterization data for PE-18.18 with 0.5 wt% PP-18

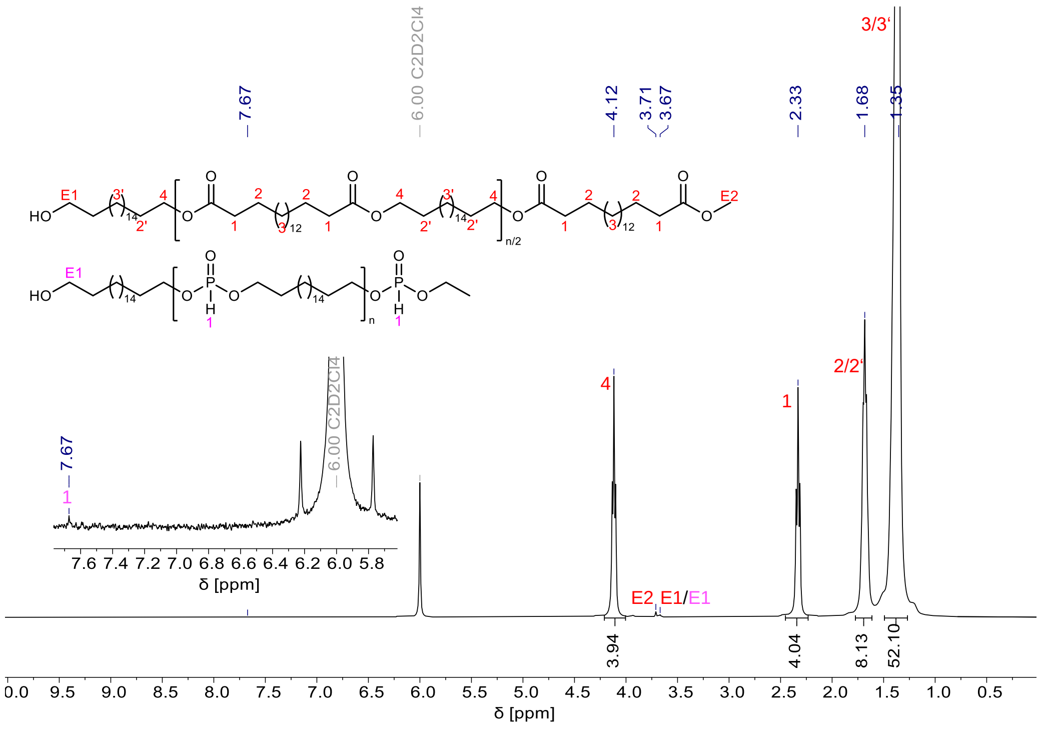

Figure S 36. ^{1}H NMR spectrum (400 MHz, $C_2D_2Cl_4$, 383 K) of PE-18.18 blended with 0.5 wt% PP-18. The enlarged excerpt shows the resonances stemming from the PP-18 blend component.

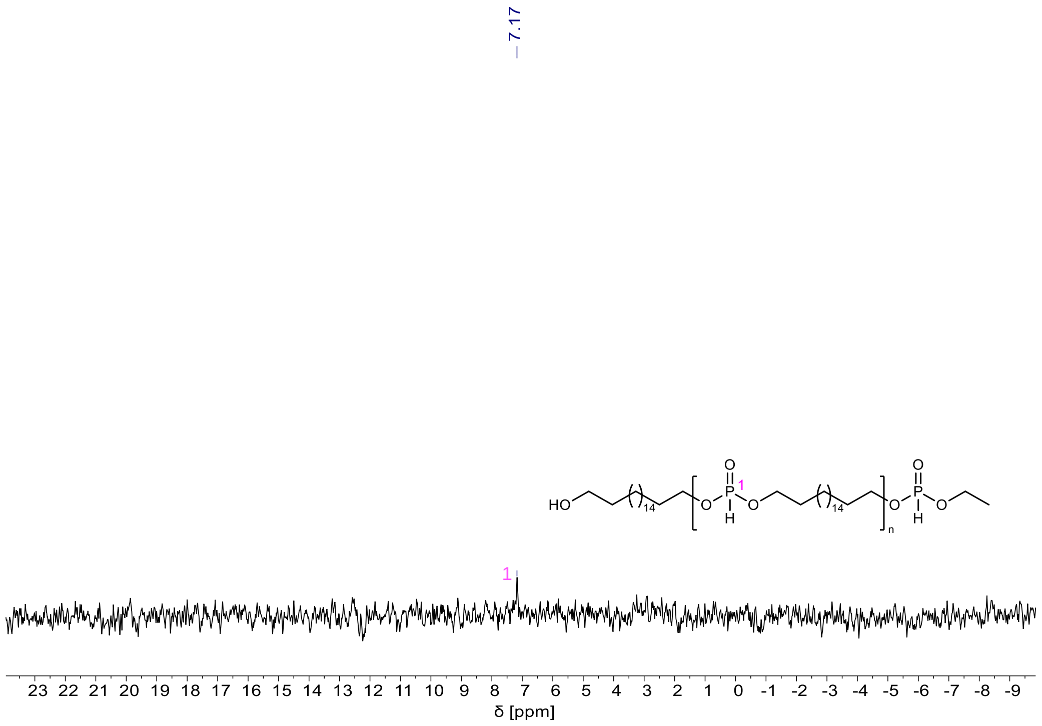

Figure S 37. $^{31}P\{^{1}H\}$ NMR spectrum (162 MHz, $C_2D_2Cl_4$, 383 K) of PE-18.18 blended with 0.5 wt% PP-18.

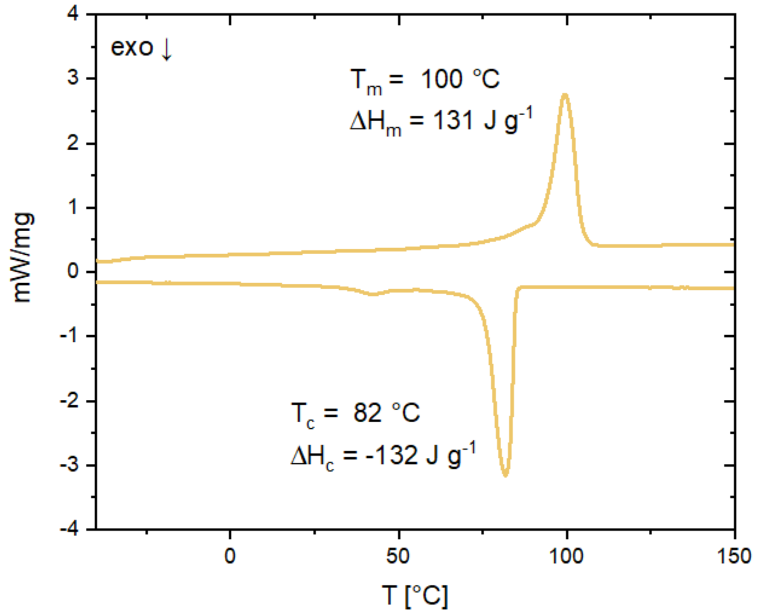

Figure S 38. DSC trace of PE-18.18 blended with 0.5 wt% PP-18.

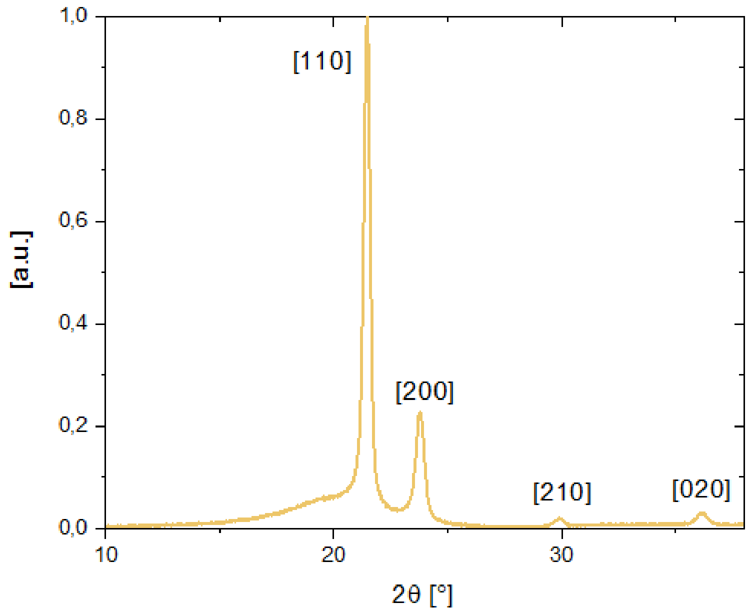

Figure S 39. WAXS diffractogram of PE-18.18 blended with 0.5 wt% PP-18. The crystallinity determined by deconvolution of the crystalline reflexes and the amorphous halo amounts to 69 %.

Additional characterization data for PE-18.18 with 2 wt% PP-18

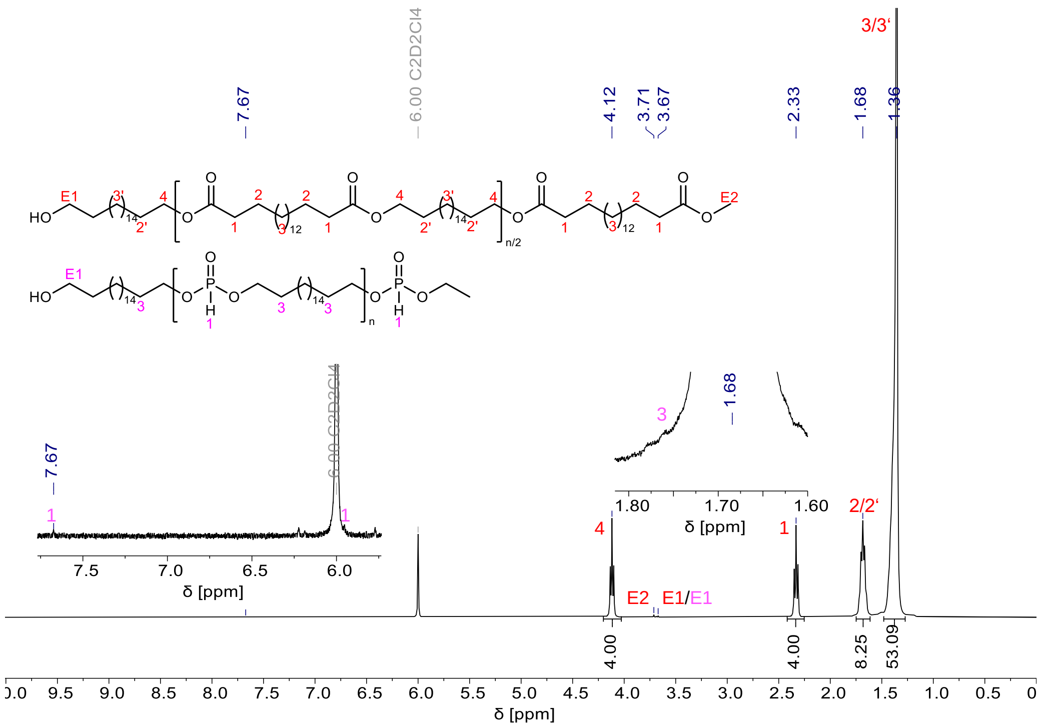

Figure S 40. ^{1}H NMR spectrum (400 MHz, $C_2D_2Cl_4$, 383 K) of PE-18.18 blended with 2 wt% PP-18. The enlarged excerpts show the resonances stemming from the PP-18 blend component.

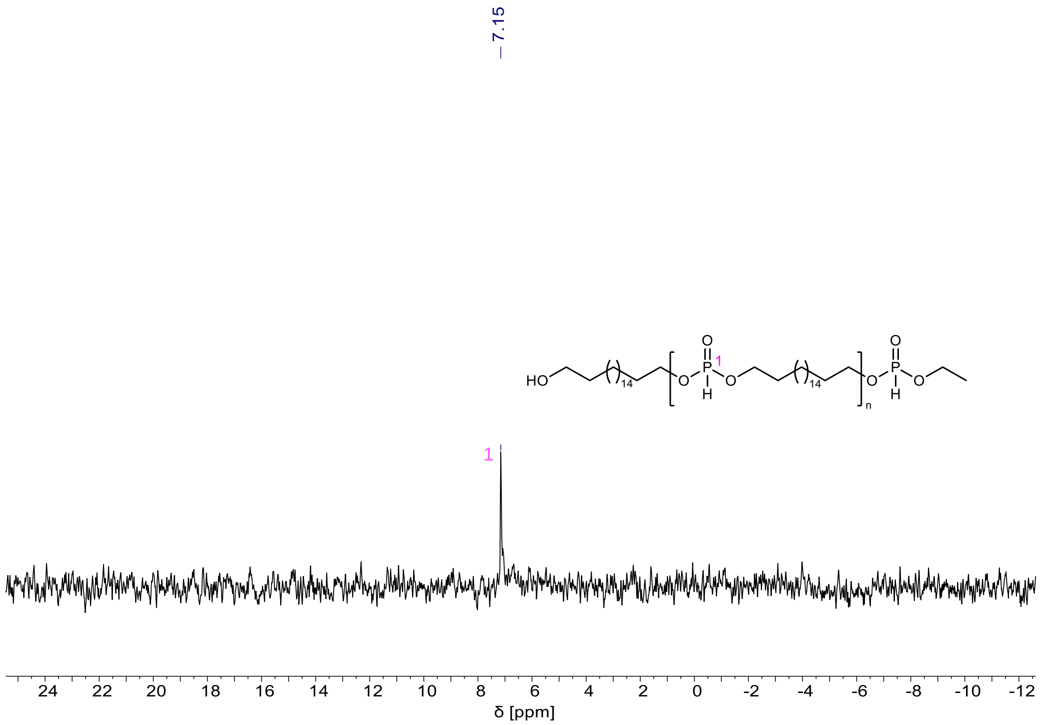

Figure S 41. $^{31}P\{^{1}H\}$ NMR spectrum (162 MHz, $C_2D_2Cl_4$, 383 K) of PE-18.18 blended with 2 wt% PP-18.

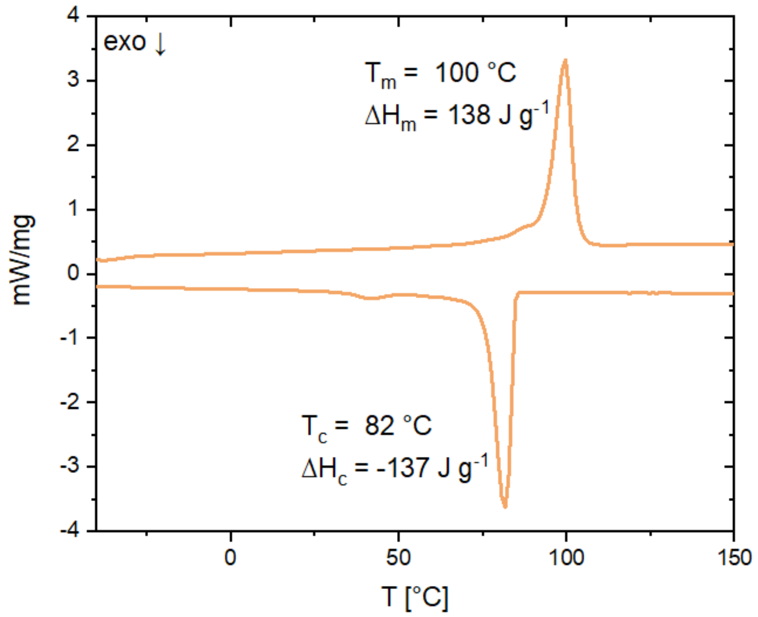

Figure S 42. DSC trace of PE-18.18 blended with 2 wt% PP-18.

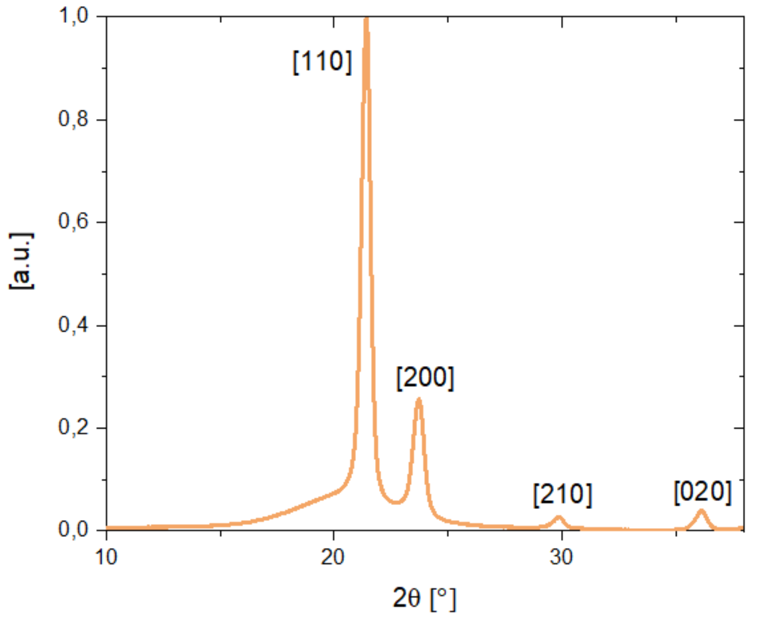

Figure S 43. WAXS diffractogram of PE-18.18 blended with 2 wt% PP-18. The crystallinity determined by deconvolution of the crystalline reflexes and the amorphous halo amounts to 73 %.

Additional characterization data for PE-18.18 with 10 wt% PP-18

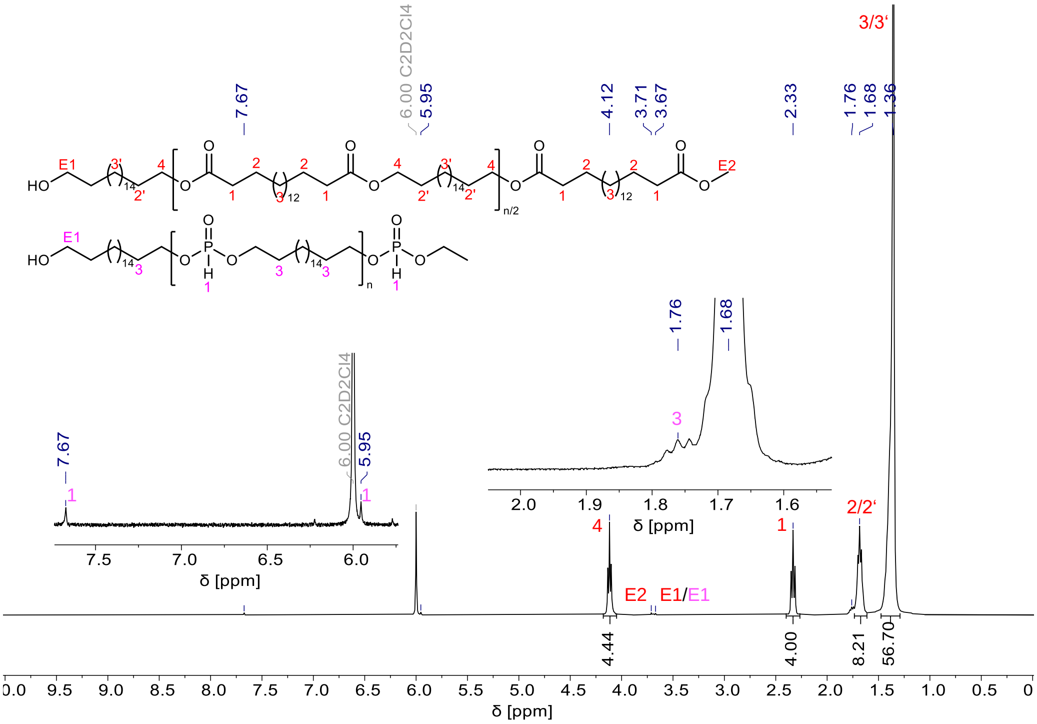

Figure S 44. ^{1}H NMR spectrum (400 MHz, $C_2D_2Cl_4$, 383 K) of PE-18.18 blended with 10 wt% PP-18. The enlarged excerpts show the resonances stemming from the PP-18 blend component.

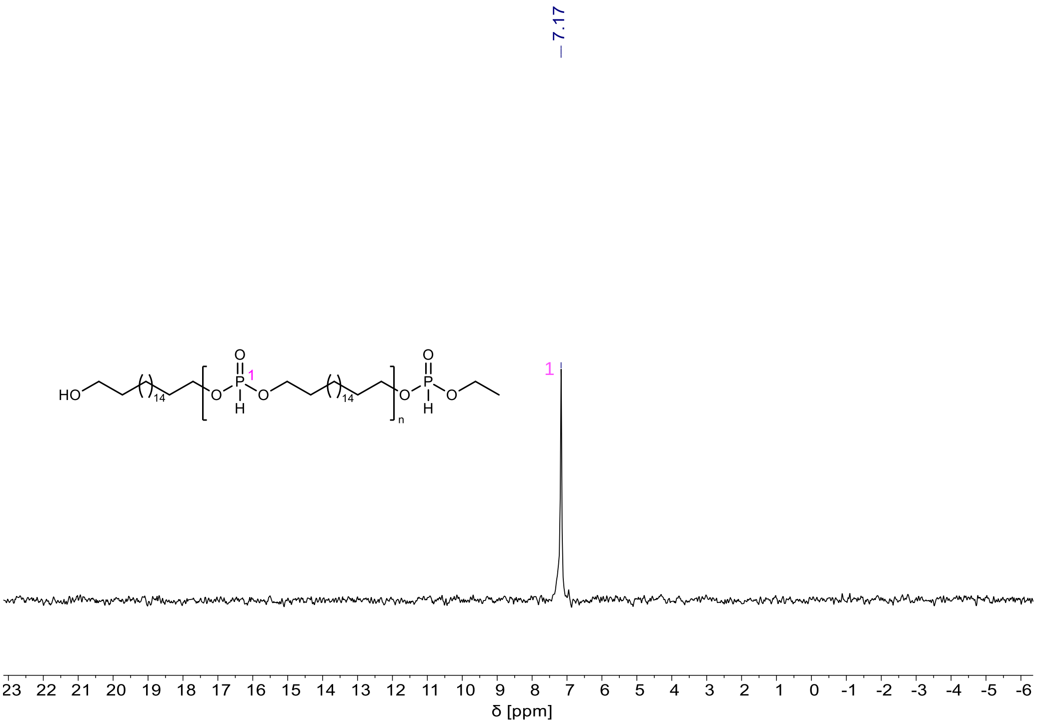

Figure S 45. $^{31}P\{^{1}H\}$ NMR spectrum (162 MHz, $C_2D_2Cl_4$, 383 K) of PE-18.18 blended with 10 wt% PP-18.

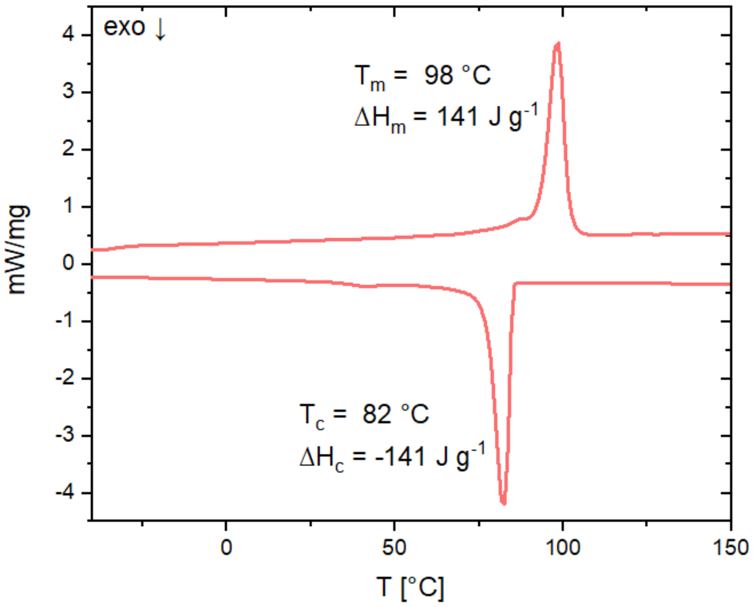

Figure S 46. DSC trace of PE-18.18 blended with 10 wt% PP-18.

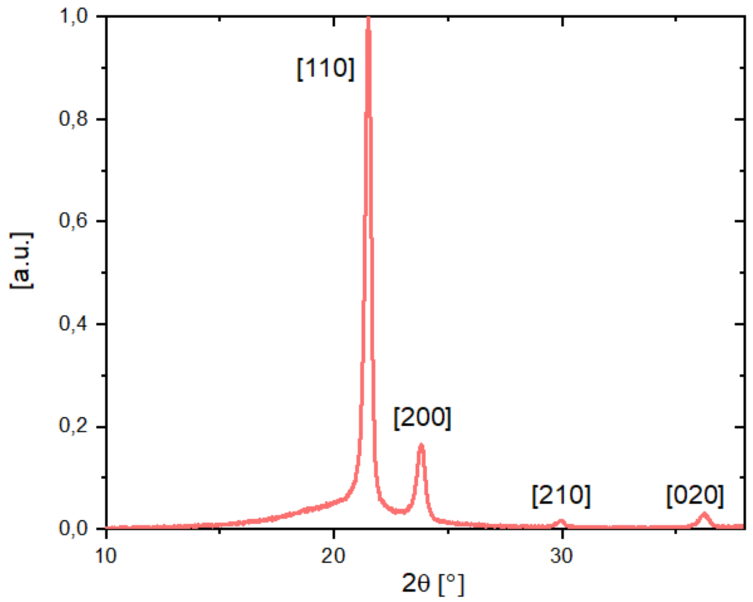

Figure S 47. WAXS diffractogram of PE-18.18 blended with 10 wt% PP-18. The crystallinity determined by deconvolution of the crystalline reflexes and the amorphous halo amounts to 65 %.

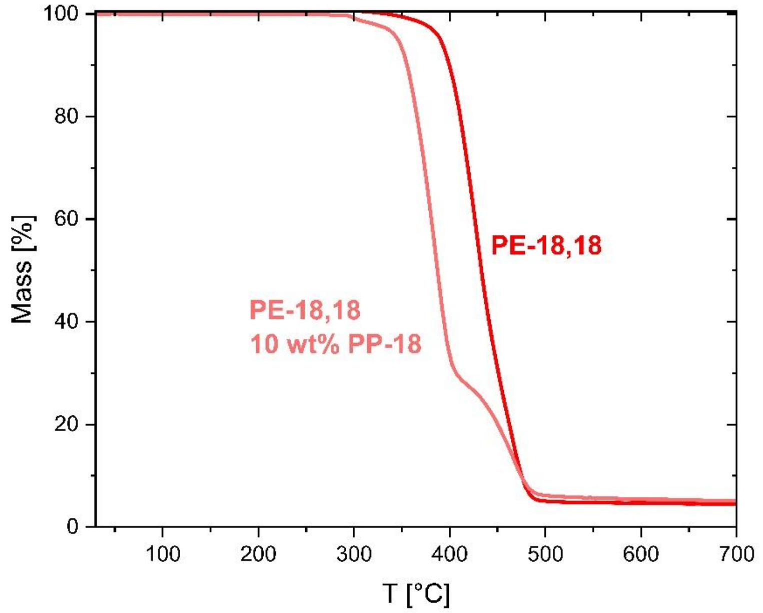

Figure S 48. TGA traces of PE-18.18 and its blend with 10 wt% PP-18. 5 % weight loss was observed at T ≈ 389 °C for PE-18.18 and at T ≈ 347 °C for PE-18.18 blended with 10 wt% PP-18.

Tensile properties of PE-18.18/PP-18 blends

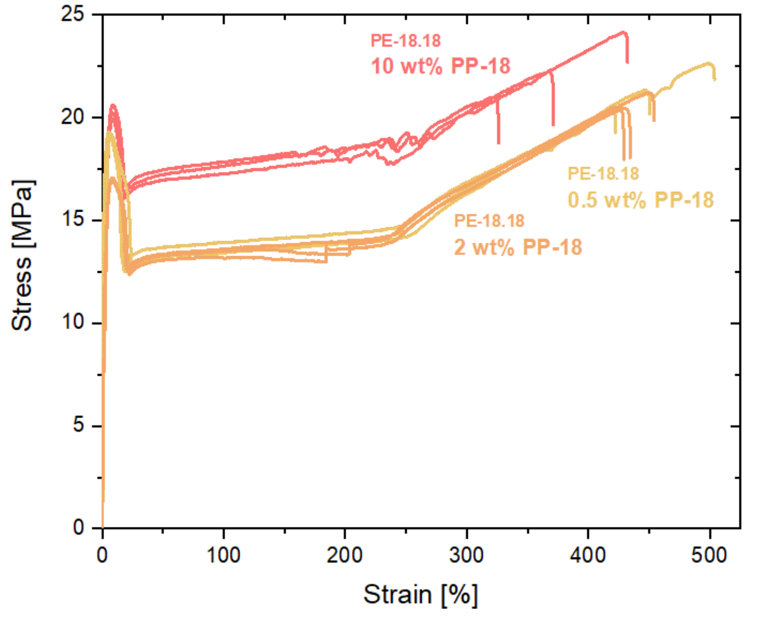

Figure S 49. Stress-strain curves of PE-18.18/PP-18 blends.

Filament extrusion process

The premixed polymers PE-18.18 (99.5 wt%, 9.95 g) and PP-18 (0.5 wt%, 0.05 g) were compounded in a Xplore MC 15 micro compounder at 160 °C and 15 rpm for 15 min. The filament was then extruded through a nozzle as reported previously[1], fitted additionally with a water-cooled metal ring. The extrusion conditions and the properties of the obtained filament are shown in **Table S 1**.

Table S 1. Extrusion conditions and properties of the obtained filament.

	$T_{extrusion}$ [°C]	Screw speed [rpm]	Measured torque [Nm]	Filament diameter [mm]
**PE-18.18/ PP-18 (0.5 wt%)**	170	4	1.64	1.69 ± 0.02

Additional characterization of PE-18.18/PP-18 (0.5 wt%) blend filament

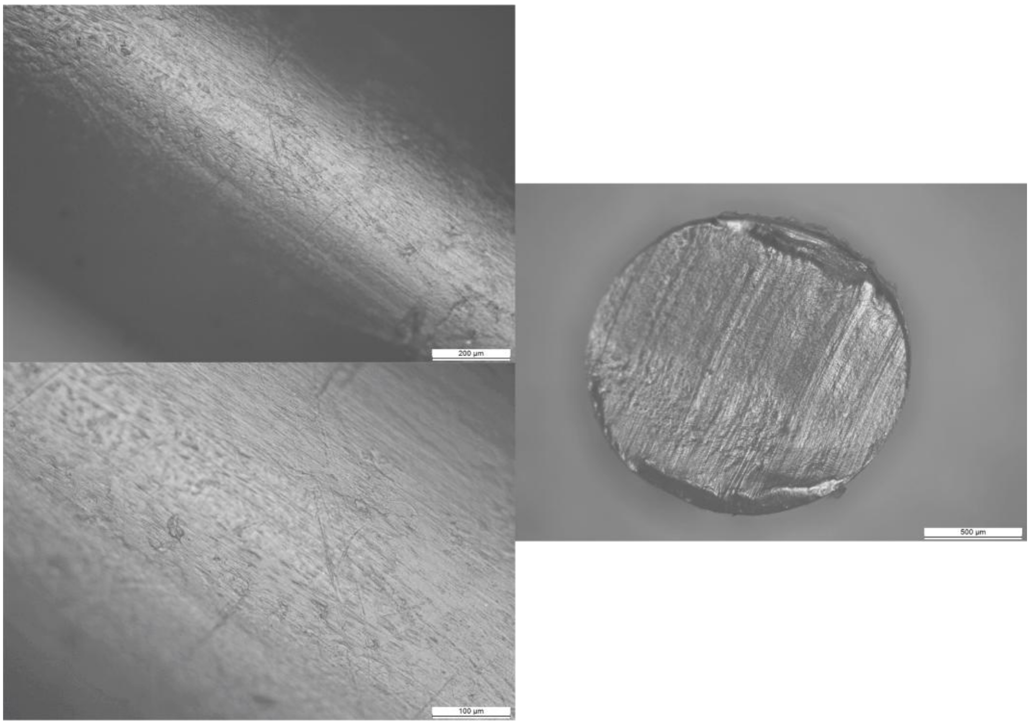

Figure S 50. Light microscopy images of the surface (left) and the cross-section (right) of PE-18.18/PP-18 (0.5 wt%) filament.

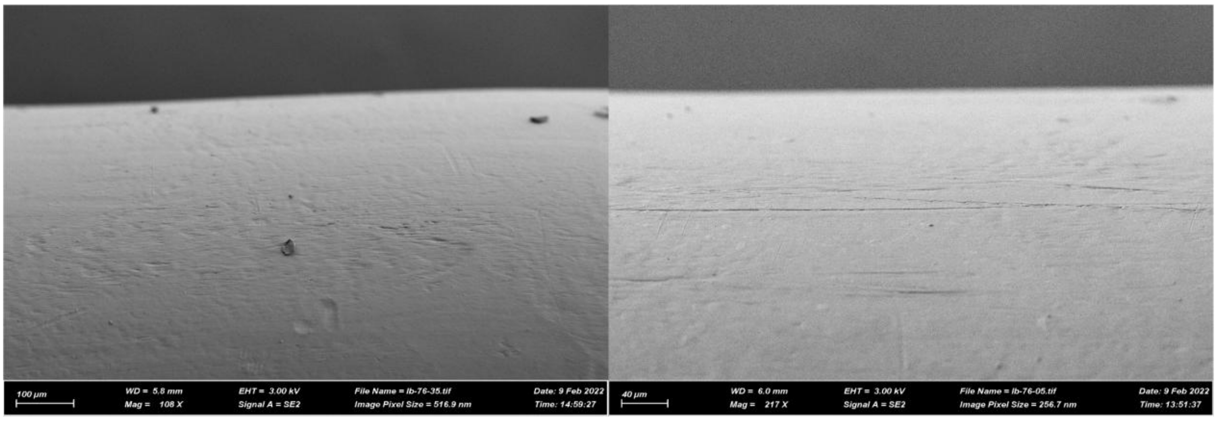

Figure S 51. SEM images of the surface of PE-18.18/PP-18 (0.5 wt%) filament at different magnifications.

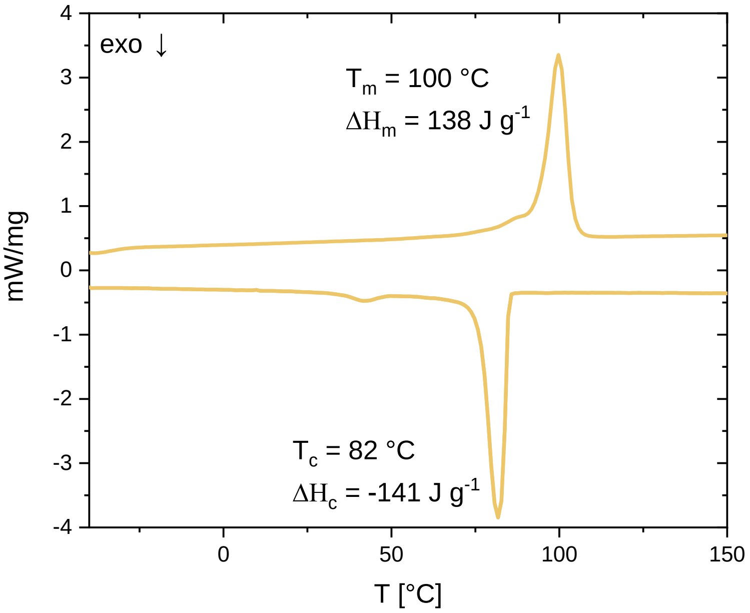

Figure S 52. DSC trace of PE-18.18/PP-18 (0.5 wt%) blend filament.

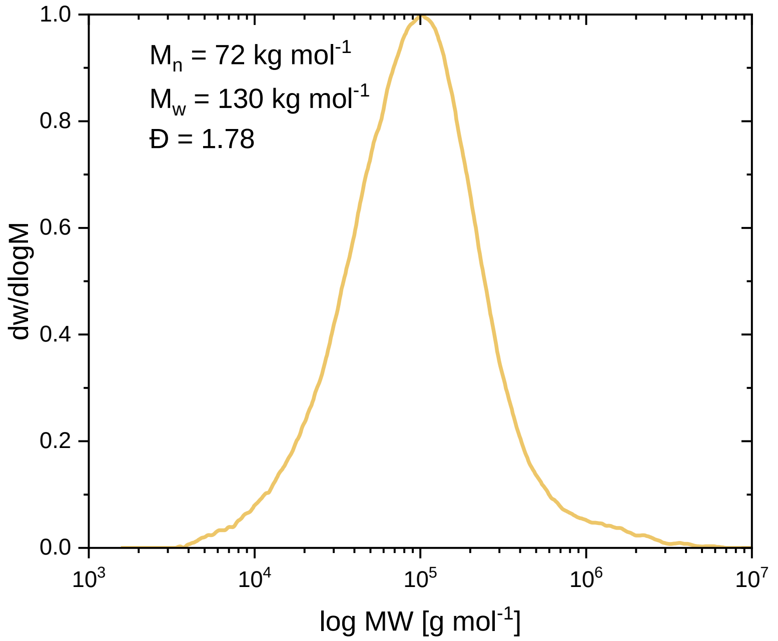

Figure S 53. GPC trace of PE-18.18/PP-18 (0.5 wt%) blend filament.

3D printing parameters

3D printed object: tensile test specimens according to ISO 527-2, type 5A.

Major optimized parameters:

Nozzle diameter	Nozzle temperature	Bed temperature	Layer thickness	Line width	Fill angle	Printing speed	Fan speed
0.80 mm	210 °C	100 °C	0.40 mm	0.80 mm	90 °/0 °	5 mm/s	0%

Significant parameters of the GCODE:

```
; external perimeters extrusion width = 0.80mm
; perimeters extrusion width = 0.80mm
; infill extrusion width = 0.80mm
; solid infill extrusion width = 0.80mm
; top infill extrusion width = 0.80mm
; first layer extrusion width = 0.80mm
M73 P0 R18
M73 Q0 S18
M201 X1000 Y1000 Z200 E5000 ; sets maximum accelerations, mm/sec^2
M203 X200 Y200 Z12 E120 ; sets maximum feedrates, mm/sec
M204 P1250 R1250 T1250 ; sets acceleration (P, T) and retract acceleration (R), mm/sec^2
M205 X8.00 Y8.00 Z0.40 E4.50 ; sets the jerk limits, mm/sec
M205 S0 T0 ; sets the minimum extruding and travel feed rate, mm/sec
M107
;TYPE:Custom
M862.3 P "MK3S" ; printer model check
M862.1 P0.8 ; nozzle diameter check
M115 U3.10.0 ; tell printer latest fw version
```

```
G90 ; use absolute coordinates
M83 ; extruder relative mode
M104 S210 ; set extruder temp
M140 S100 ; set bed temp
M190 S100 ; wait for bed temp
M109 S210 ; wait for extruder temp
; estimated printing time (normal mode) = 17m 58s
; avoid_crossing_perimeters = 1
; avoid_crossing_perimeters_max_detour = 0
; bed_custom_model =
; bed_custom_texture =
; bed_shape = 0x0,250x0,250x210,0x210
; bed_temperature = 100
; bottom_fill_pattern = rectilinear
; bottom_solid_layers = 4
; bottom_solid_min_thickness = 0.8
; bridge_acceleration = 1000
; bridge_angle = 0
; bridge_fan_speed = 0
; bridge_flow_ratio = 0.95
; bridge_speed = 5
; brim_width = 0
; clip_multipart_objects = 1
; complete_objects = 0
; cooling = 0
; cooling_tube_length = 5
; cooling_tube_retraction = 91.5
; default_acceleration = 1000
; deretract_speed = 0
; disable_fan_first_layers = 1
; dont_support_bridges = 1
; draft_shield = 0
; duplicate_distance = 6
; elefant_foot_compensation = 0
; ensure_vertical_shell_thickness = 0
; external_perimeter_extrusion_width = 0.8
; external_perimeter_speed = 5
; external_perimeters_first = 0
; extra_loading_move = -2
; extra_perimeters = 0
; extruder_clearance_height = 20
; extruder_clearance_radius = 20
; extruder_offset = 0x0
; extrusion_axis = E
; extrusion_multiplier = 0.9
; extrusion_width = 0.8
; fan_always_on = 0
; filament_diameter = 1.7
; filament_load_time = 0
; filament_loading_speed = 28
; filament_loading_speed_start = 3
; filament_max_volumetric_speed = 0
; filament_minimal_purge_on_wipe_tower = 15
; filament_notes = ""
; filament_ramming_parameters = "120 100 6.6 6.8 7.2 7.6 7.9 8.2 8.7 9.4 9.9 10.0| 0.05 6.6 0.45 6.8 0.95 7.8 1.45 8.3 1.95 9.7 2.45 10 2.95 7.6 3.45 7.6 3.95 7.6 4.45 7.6 4.95 7.6"
```

; filament_settings_id = 0.8nozzle_220C-100C_0.4mm_1,70_-ISO_527_Type_5A
; filament_soluble = 0
; filament_spool_weight = 0
; filament_toolchange_delay = 0
; filament_type = PLA
; filament_unload_time = 0
; filament_unloading_speed = 90
; filament_unloading_speed_start = 100
; filament_vendor = (Unknown)
; fill_angle = 0
; fill_density = 100%
; fill_pattern = rectilinear
; first_layer_acceleration = 1000
; first_layer_bed_temperature = 100
; first_layer_extrusion_width = 0.8
; first_layer_height = 0.4
; first_layer_speed = 5
; first_layer_temperature = 210
; full_fan_speed_layer = 0
; gap_fill_speed = 5
; gcode_comments = 0
; gcode_flavor = marlin
; gcode_label_objects = 1
; high_current_on_filament_swap = 0
; host_type = octoprint
; infill_acceleration = 1250
; infill_anchor = 2.5
; infill_anchor_max = 20
; infill_every_layers = 1
; infill_extruder = 1
; infill_extrusion_width = 0.8
; infill_first = 0
; infill_only_where_needed = 0
; infill_overlap = 50%
; infill_speed = 5
; interface_shells = 0
; ironing = 0
; layer_height = 0.4
; machine_max_acceleration_e = 5000,5000
; machine_max_acceleration_extruding = 1250,1250
; machine_max_acceleration_retracting = 1250,1250
; machine_max_acceleration_x = 1000,960
; machine_max_acceleration_y = 1000,960
; machine_max_acceleration_z = 200,200
; machine_max_feedrate_e = 120,120
; machine_max_feedrate_x = 200,100
; machine_max_feedrate_y = 200,100
; machine_max_feedrate_z = 12,12
; machine_max_jerk_e = 4.5,4.5
; machine_max_jerk_x = 8,8
; machine_max_jerk_y = 8,8
; machine_max_jerk_z = 0.4,0.4
; machine_min_extruding_rate = 0,0
; machine_min_travel_rate = 0,0
; max_fan_speed = 100
; max_layer_height = 0.45

; max_print_height = 210
; max_print_speed = 200
; max_volumetric_speed = 0
; min_fan_speed = 35
; min_layer_height = 0.07
; min_print_speed = 15
; min_skirt_length = 4
; nozzle_diameter = 0.8
; only_retract_when_crossing_perimeters = 0
; ooze_prevention = 0
; overhangs = 0
; parking_pos_retraction = 92
; perimeter_acceleration = 800
; perimeter_extruder = 1
; perimeter_extrusion_width = 0.8
; perimeter_speed = 5
; perimeters = 1
; physical_printer_settings_id =
; post_process =
; printer_model = MK3S
; printer_technology = FFF
; printer_variant = 0.4
; printer_vendor =
; raft_layers = 0
; remaining_times = 1
; resolution = 0
; retract_before_travel = 1
; retract_before_wipe = 0%
; retract_layer_change = 1
; retract_length = 0
; retract_length_toolchange = 4
; retract_lift = 0.4
; retract_lift_above = 0
; retract_lift_below = 209
; retract_restart_extra = 0
; retract_restart_extra_toolchange = 0
; retract_speed = 35
; seam_position = nearest
; silent_mode = 1
; single_extruder_multi_material = 0
; single_extruder_multi_material_priming = 0
; skirt_distance = 3
; skirt_height = 2
; skirts = 1
; slice_closing_radius = 0.049
; slowdown_below_layer_time = 20
; small_perimeter_speed = 5
; solid_infill_below_area = 0
; solid_infill_every_layers = 0
; solid_infill_extruder = 1
; solid_infill_extrusion_width = 0.8
; solid_infill_speed = 5
; spiral_vase = 0
; standby_temperature_delta = -5
; start_filament_gcode = "; Filament gcode\n"
; support_material = 0

; temperature = 210
; template_custom_gcode =
; thin_walls = 0
; threads = 4
; thumbnails =
; toolchange_gcode =
; top_fill_pattern = rectilinear
; top_infill_extrusion_width = 0.8
; top_solid_infill_speed = 5
; top_solid_layers = 4
; top_solid_min_thickness = 1.2
; travel_speed = 180
; use_firmware_retraction = 0
; use_relative_e_distances = 1
; use_volumetric_e = 0
; variable_layer_height = 1
; wipe = 1
; wipe_into_infill = 0
; wipe_into_objects = 0
; wipe_tower = 1
; wipe_tower_bridging = 10
; wipe_tower_no_sparse_layers = 0
; wipe_tower_rotation_angle = 0
; wipe_tower_width = 60
; wipe_tower_x = 170
; wipe_tower_y = 140
; wiping_volumes_extruders = 70,70
; wiping_volumes_matrix = 0
; xy_size_compensation = 0
; z_offset = 0

Additional characterization of PE-18.18/PP-18 (0.5 wt%) blend after 3D printing

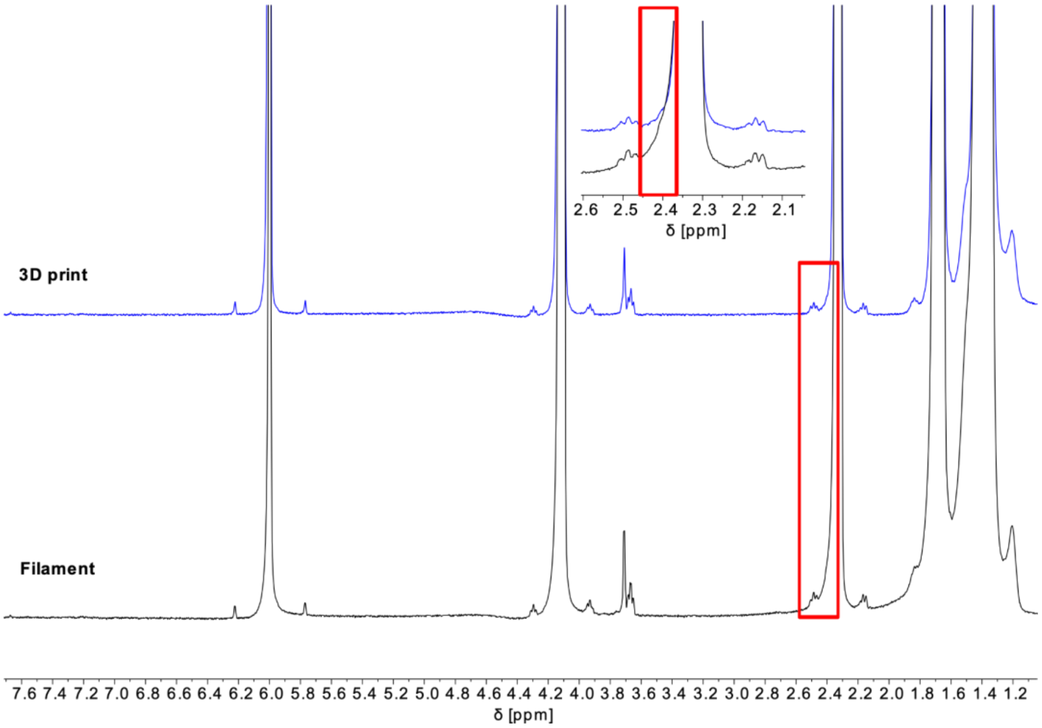

Figure S 54. Stacked ^{1}H NMR spectra (400 MHz, $C_2D_2Cl_4$, 383 K) of the PE-18.18/PP-18 (0.5 wt%) blend filament before (bottom) and after (top) 3D printing. The absence of the carboxylic acid end group resonance (δ = 2.39 ppm) in the enlarged excerpt shows that no degradation of the blend has occurred during the 3D printing process.

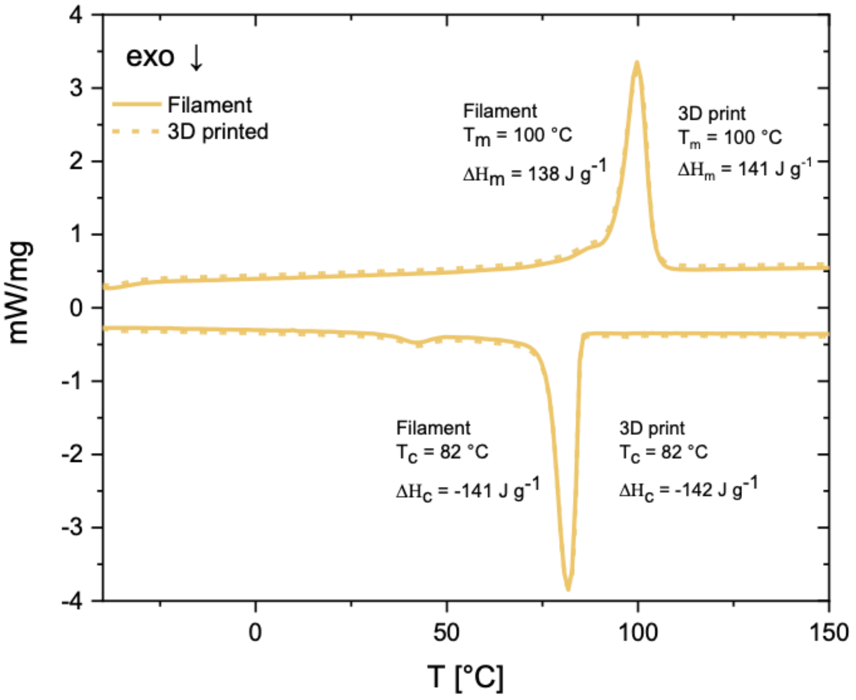

Figure S 55. DSC traces of the PE-18.18/PP-18 (0.5 wt%) blend filament before (bold) and after (dashed) 3D printing.

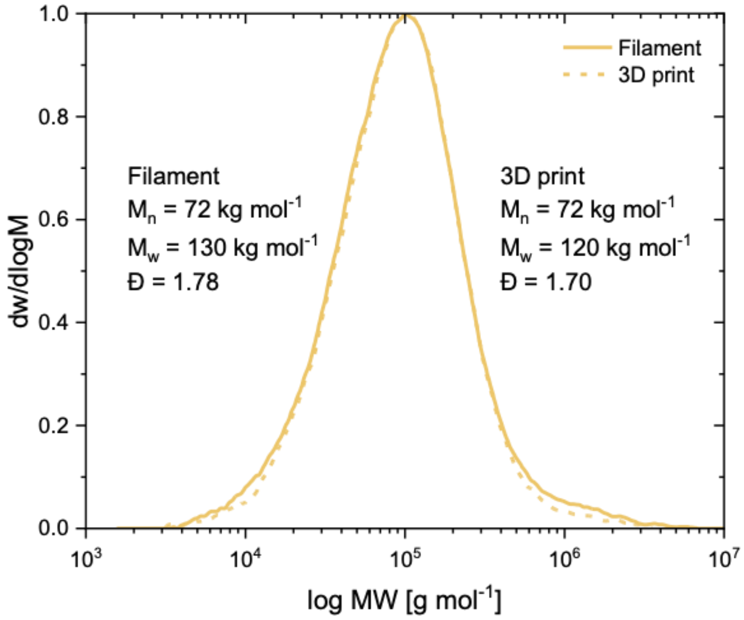

Figure S 56. GPC traces of PE-18.18/PP-18 (0.5 wt%) blend filament before (bold) and after (dashed) 3D printing.

Experimental setup of the hydrolysis experiments

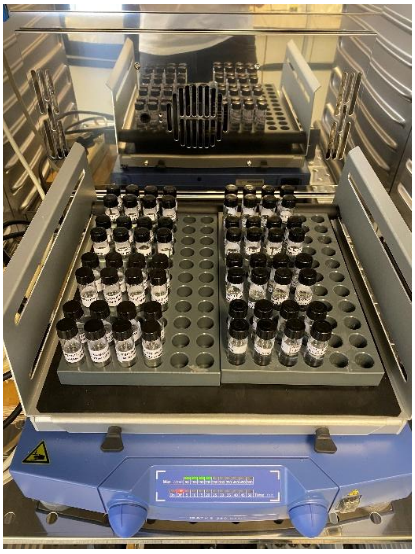

Figure S 57. Experimental setup of the hydrolysis experiments for PE-18.18 and its PP-18 and PP-26 blends. The orbital shaker with the holder containing the sealed glass vials is placed in a light-proof Peltier temperature-controlled cabinet.

Optical impression of the PE-18.18/PP-26 blend specimens exposed to hydrolysis media

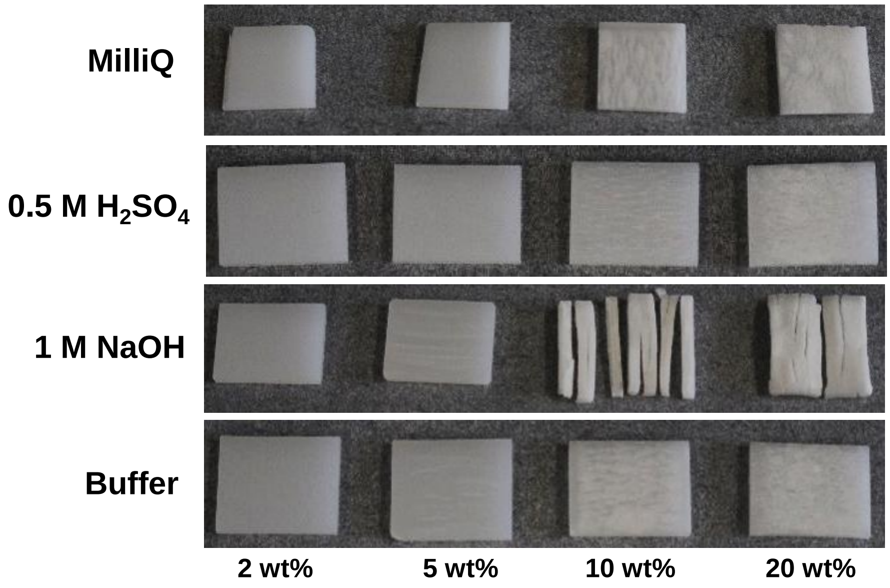

Figure S 58. Injection molded PE-18.18/PP-26 blend specimens after 16 weeks in MilliQ water, 0.5 M H_2SO_4, 1 M NaOH and phosphate buffer (pH = 7). The labels below the specimens refer to the contents of PP-26 in the blends.

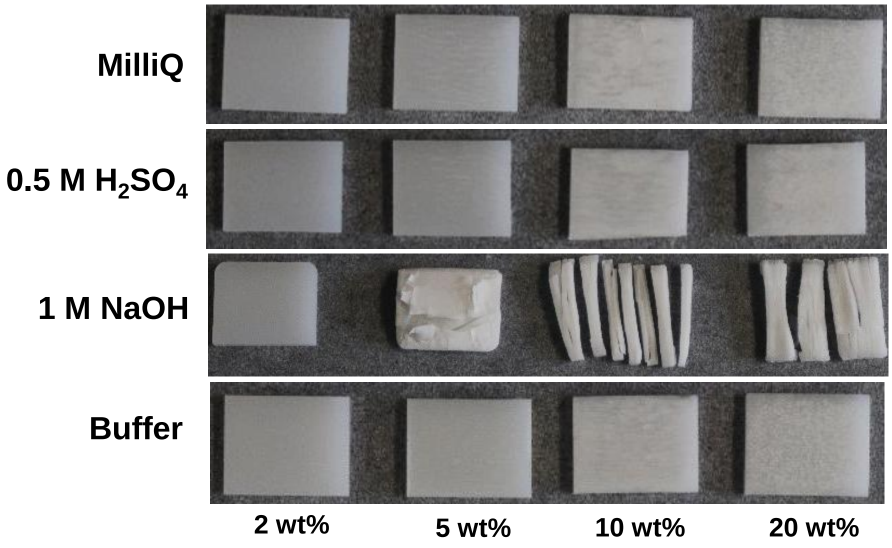

Figure S 59. Injection molded PE-18.18/PP-26 blend specimens after 32 weeks in MilliQ water, 0.5 M H_2SO_4, 1 M NaOH and phosphate buffer (pH = 7). The labels below the specimens refer to the contents of PP-26 in the blends.

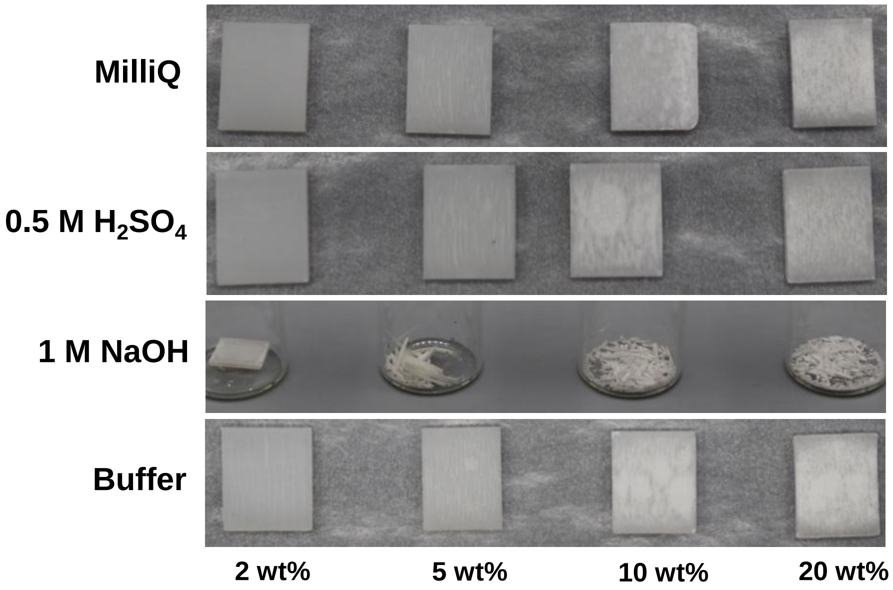

Figure S 60. Injection molded PE-18.18/PP-26 blend specimens after 48 weeks in MilliQ water, 0.5 M H_2SO_4, 1 M NaOH and phosphate buffer (pH = 7). The labels below the specimens refer to the contents of PP-26 in the blends. The 5, 10 and 20 wt% PP-26 blends fragmented in 1 M NaOH.

Optical impression of the PE-18.18/PP-18 blend specimens exposed to hydrolysis media

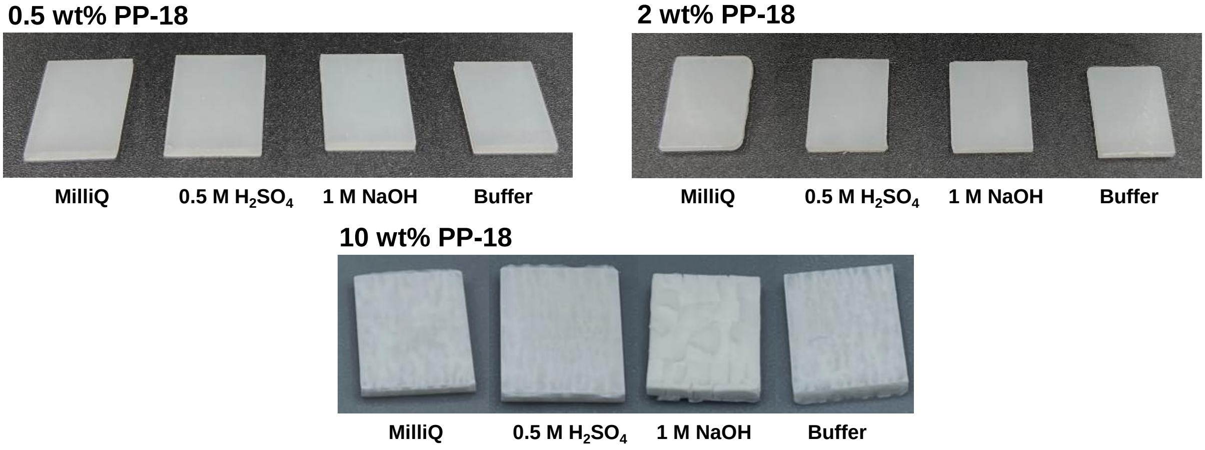

Figure S 61. Injection molded specimens of PE-18.18/PP-18 blends after 16 weeks in MilliQ water, 0.5 M H_2SO_4, 1 M NaOH and phosphate buffer (pH = 8). The labels below the specimens refer to the hydrolysis media.

SEM images of the PE-18.18/PP-26 blend specimens exposed to hydrolysis media

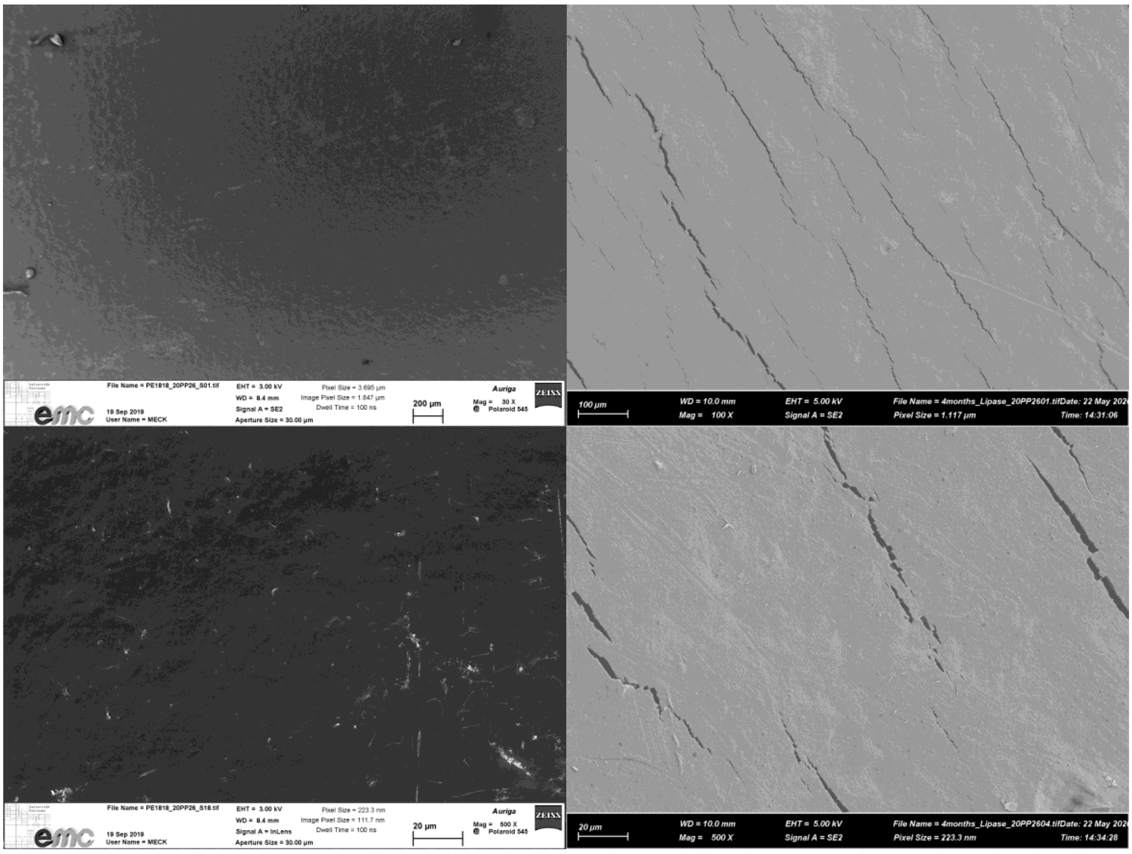

Figure S 62. SEM images of injection molded specimens of PE-18.18 blended with 20 wt% PP-26 before (left) and after 16 weeks in phosphate buffer (pH = 7) (right) at different magnifications.

Weight change of the PE-18.18/PP-26 blend specimens exposed to hydrolysis media

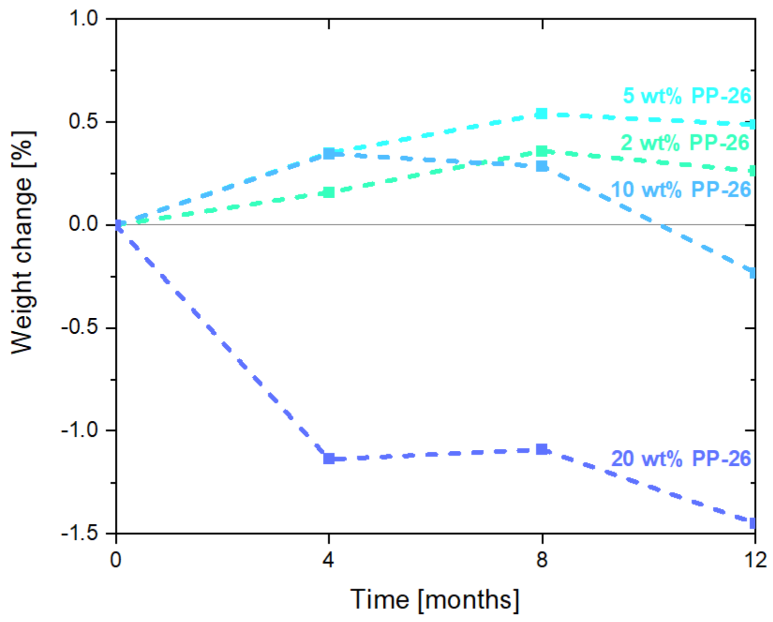

Figure S 63. Weight change of the injection molded PE-18.18/PP-26 specimens after 4, 8 and 12 months in MilliQ water at 25 °C. Connecting lines are a guide to the eye.

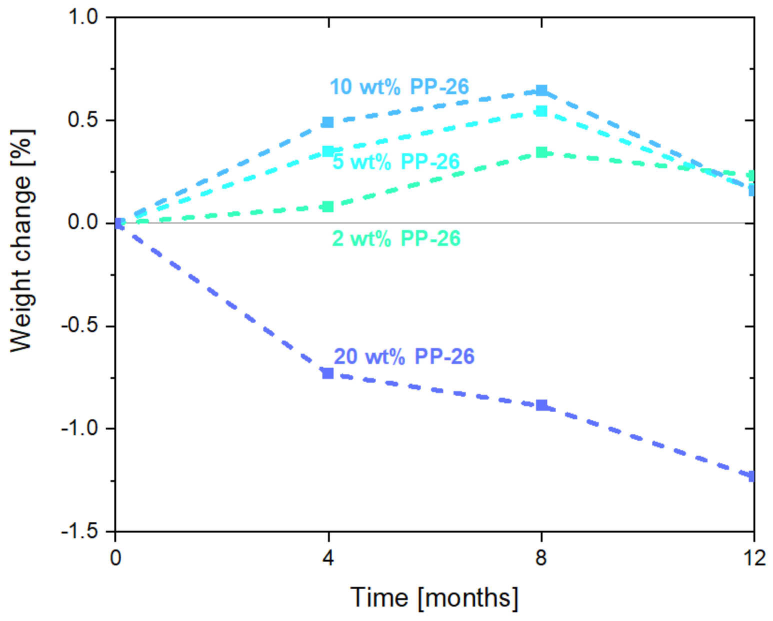

Figure S 64. Weight change of the injection molded PE-18.18/PP-26 specimens after 4, 8 and 12 months in 0.5 M H_2SO_4 at 25 °C. Connecting lines are a guide to the eye.

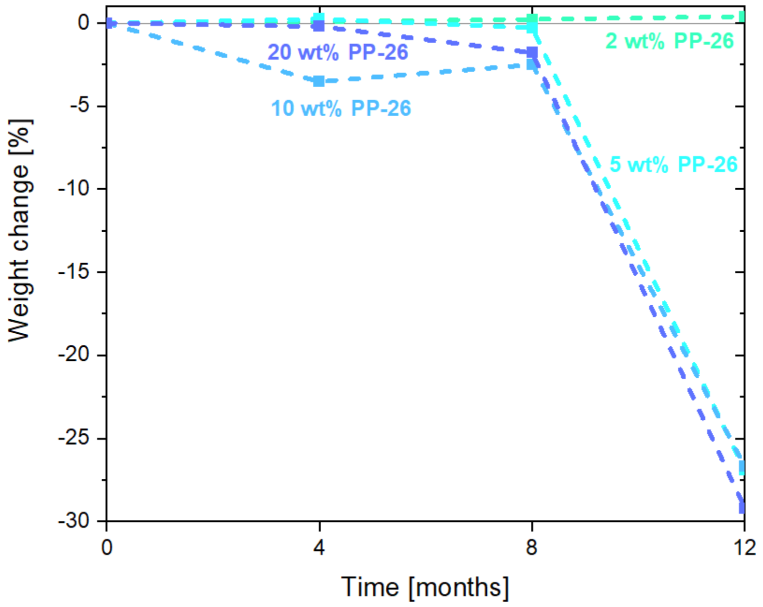

Figure S 65. Weight change of the injection molded PE-18.18/PP-26 specimens after 4, 8 and 12 months in 1 M NaOH at 25 °C. Fragmentation of the blend specimens containing 5, 10 and 20 wt% PP-26 impeded determination of the weight changes. Connecting lines are a guide to the eye.

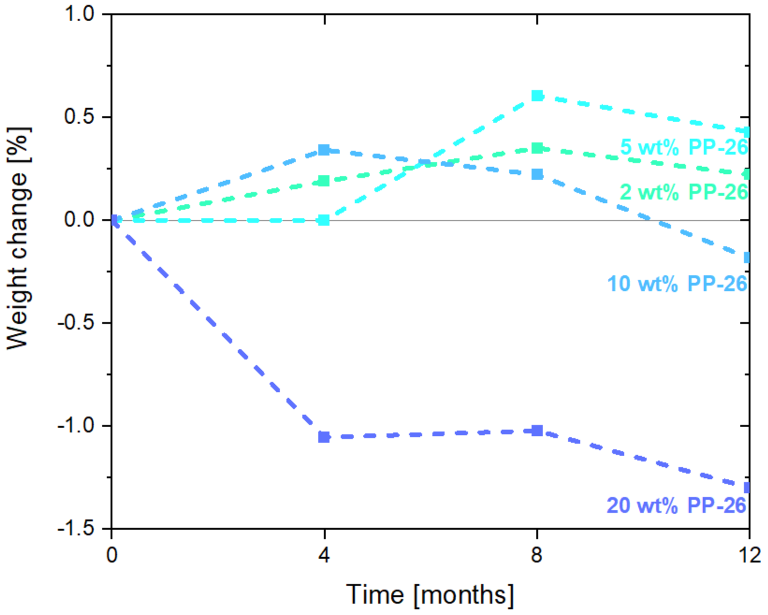

Figure S 66. Weight change of the injection molded PE-18.18/PP-26 specimens after 4, 8 and 12 months in buffer (pH = 7) at 25 °C. Connecting lines are a guide to the eye.

The increase in weight of the 0.5 – 10 wt% poly(H-phosphonate) containing blend samples upon exposure to hydrolysis media is assumed to be a result of residual water not completely removed after drying.

Assignment of the ^{1}H NMR resonances of the blends exposed to hydrolysis media

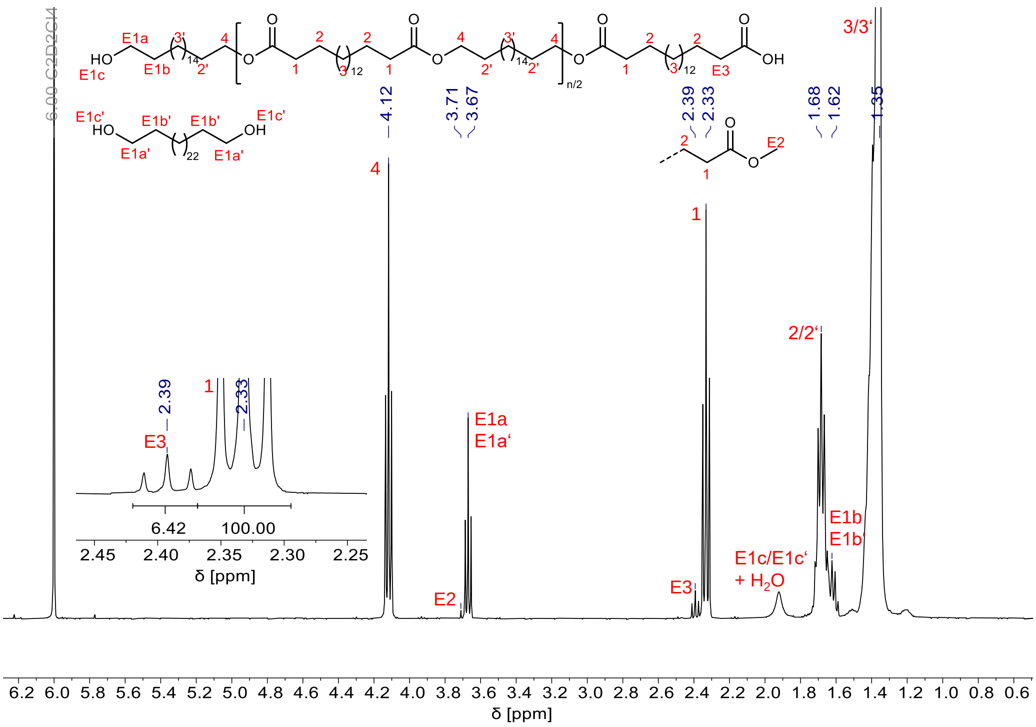

Figure S 67. ^{1}H NMR spectrum (400 MHz, $C_2D_2Cl_4$, 383 K) of PE-18.18 blended with 20 wt% PP-26 after 12 months in buffer (pH = 7). The enlarged excerpt shows the carboxylic acid end group resonance E3 (δ = 2.39 ppm) resulting from the hydrolysis of the PE-18.18 blend component. The chemical shift related to the exchange resonance of the hydroxy resonance E1c and water is variable.

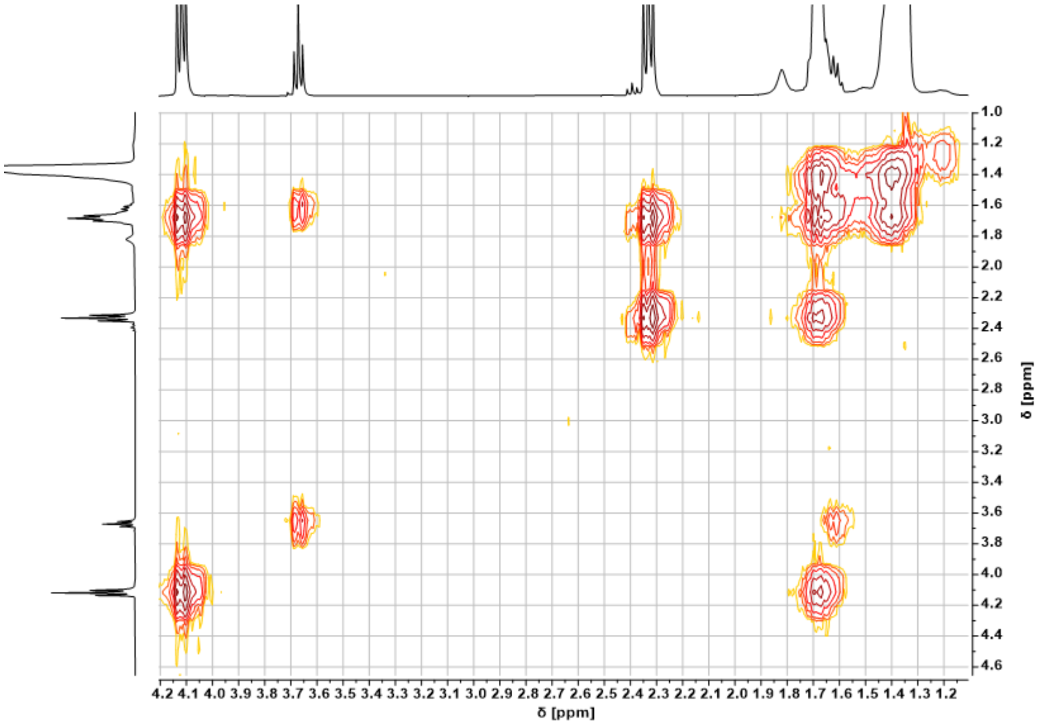

Figure S 68. ^{1}H-^{1}H COSY NMR spectrum (400 MHz/400 MHz, $C_2D_2Cl_4$, 383 K) of PE-18.18 blended with 20 wt% PP-26 after 12 months in buffer (pH = 7).

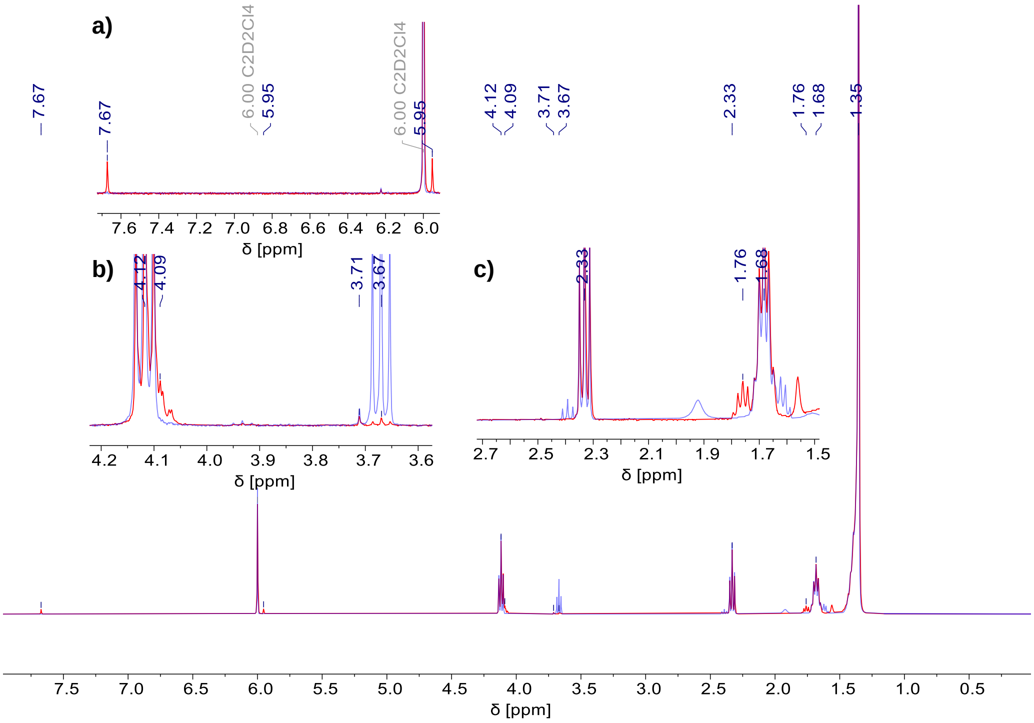

Figure S 69. Superimposed ^{1}H NMR spectra (400 MHz, $C_2D_2Cl_4$, 383 K) of the virgin PE-18.18/PP-26 (20 wt%) blend (red) and the blend after 12 months in buffer (pH = 7) (blue). The hydrolysis of the PP-26 component results in the disappearance of the related resonances and the appearance or intensification of the resonances belonging to the hydrolysis products (shown in the enlarged excerpts).

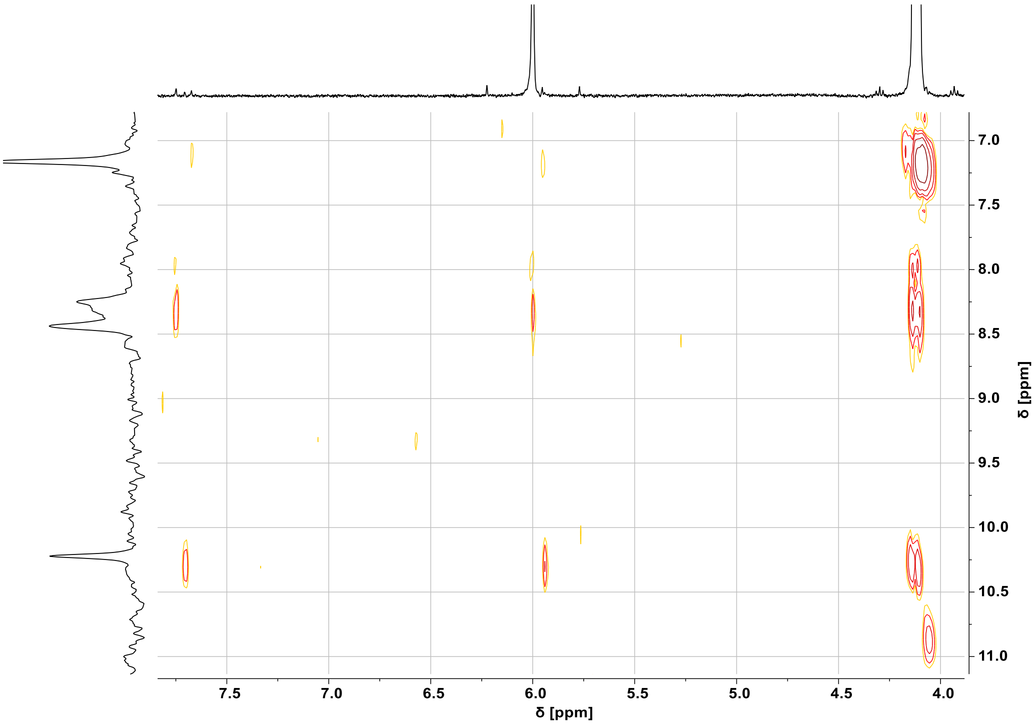

Figure S 70. ^{1}H-^{31}P HMBC NMR spectrum (400 MHz/162 MHz, $C_2D_2Cl_4$, 383 K) of the PE-18.18/PP-26 (20 wt%) blend after 4 months in MilliQ water. The correlations show that the residual ^{31}P species present in the blends after hydrolysis are H-phosphonate esters. The correlation between the ^{31}P resonance with σ = 7.17 ppm and the P-H proton is additionally shown in the ^{31}P NMR spectrum of **Figure S 71**.

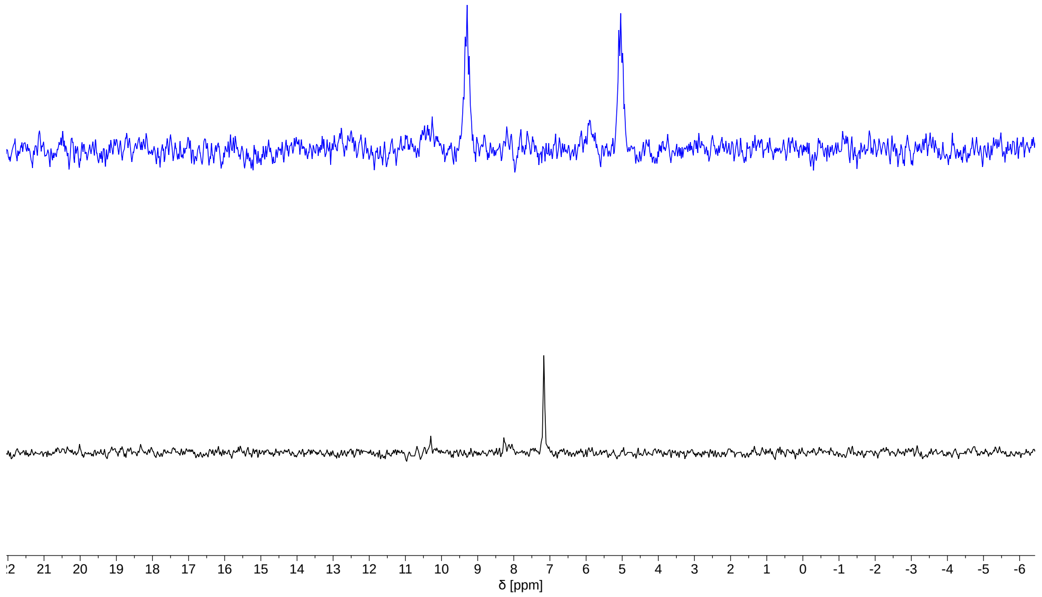

Figure S 71. Stacked $^{31}P\{^1H\}$ NMR (bottom) and ^{31}P NMR (top) spectra (162 MHz, $C_2D_2Cl_4$, 383 K) of the PE-18.18/PP-26 (20 wt%) blend after 4 months in MilliQ water. The $^1J_{31P,1H}$ coupling constant of the ^{31}P resonance with σ = 7.17 ppm is 689 Hz.

NMR analysis of the PE-18.18/PP-26 blends exposed to hydrolysis media

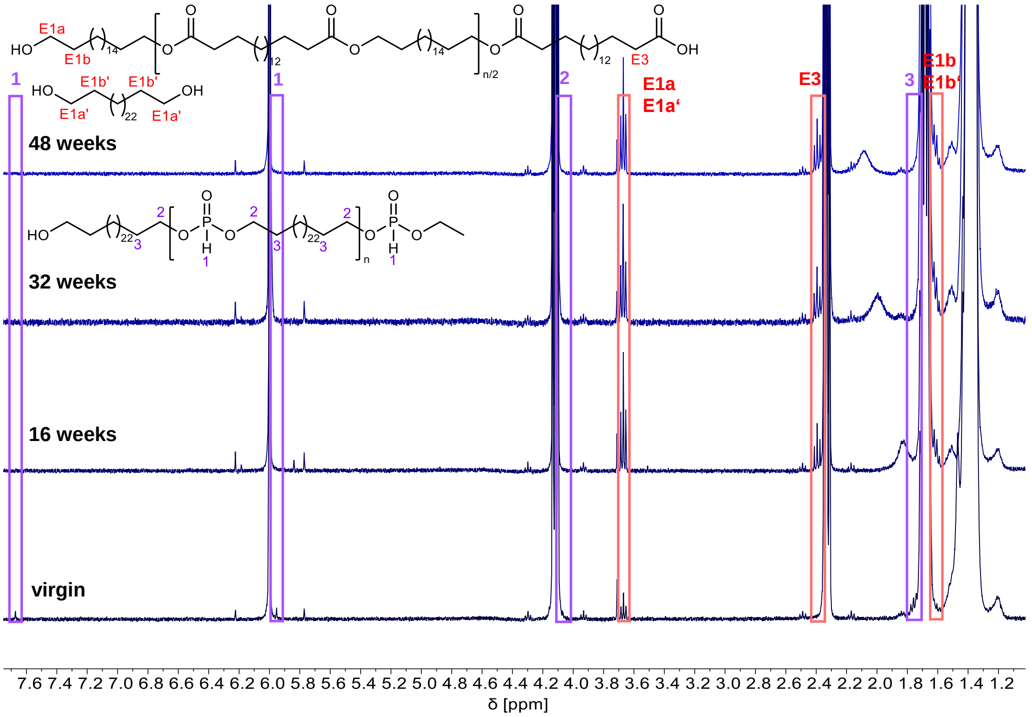

Figure S 72. Stacked ^1H NMR spectra (400 MHz, $C_2D_2Cl_4$, 383 K) of the virgin PE-18.18/PP-26 (2 wt%) blend and the blend after 4, 8 and 12 months in buffer (pH = 7). The purple markers highlight the PP-26 resonances disappearing upon hydrolysis while the red markers highlight the appearing or intensifying resonances stemming from the hydrolysis products.

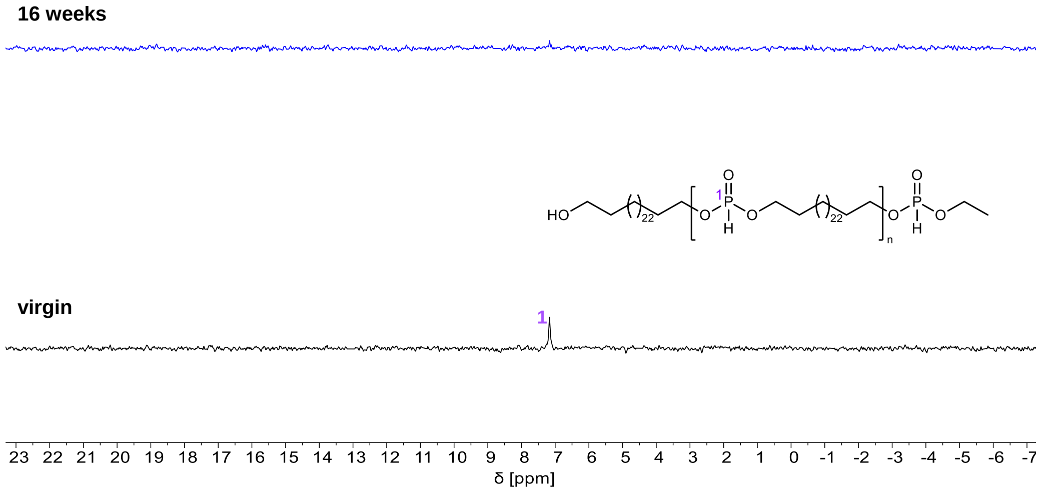

Figure S 73. Stacked $^{31}P\{^{1}H\}$ NMR spectra (162 MHz, $C_2D_2Cl_4$, 383 K) of the virgin PE-18.18/PP-26 (2 wt%) blend and the blend after 4 months in buffer (pH = 7).

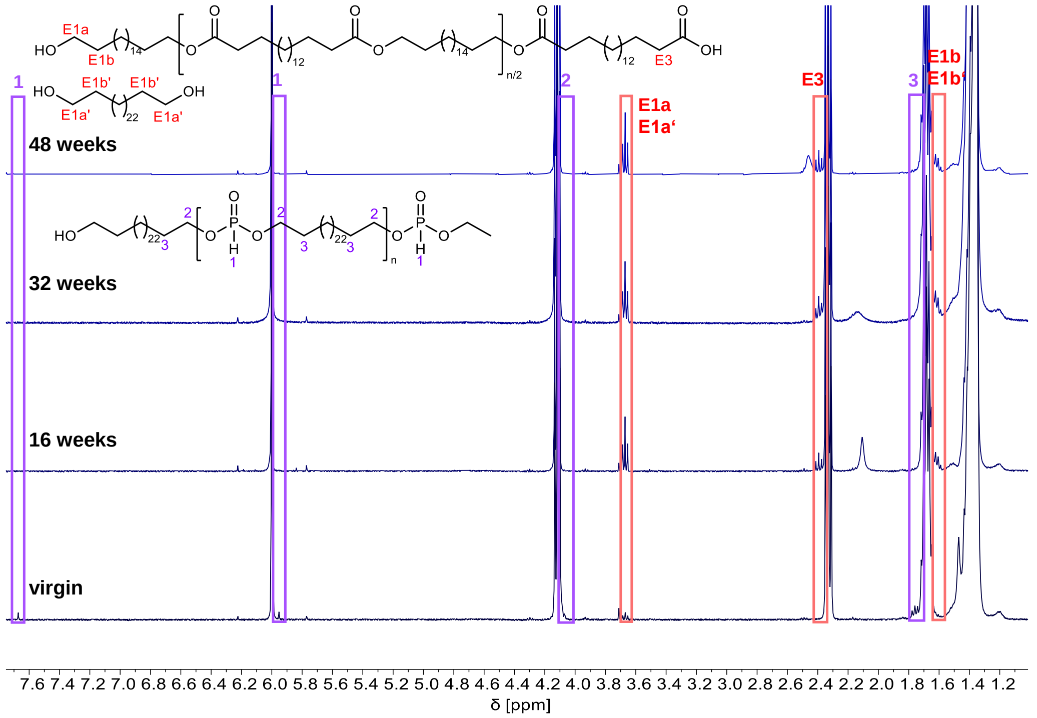

Figure S 74. Stacked ^{1}H NMR spectra (400 MHz, $C_2D_2Cl_4$, 383 K) of the virgin PE-18.18/PP-26 (5 wt%) blend and the blend after 4, 8 and 12 months in buffer (pH = 7). The purple markers highlight the PP-26 resonances disappearing upon hydrolysis while the red markers highlight the appearing or intensifying resonances stemming from the hydrolysis products.

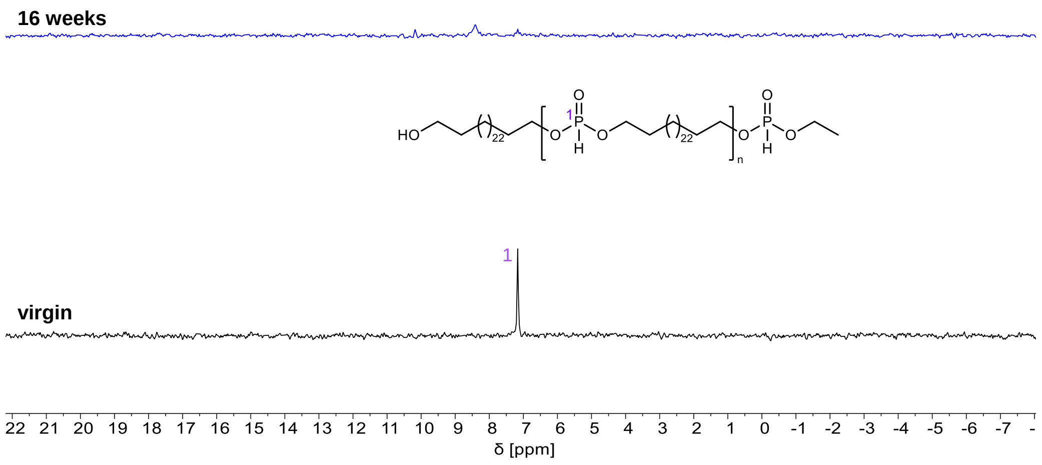

Figure S 75. Stacked $^{31}P\{^{1}H\}$ NMR spectra (162 MHz, $C_2D_2Cl_4$, 383 K) of the virgin PE-18.18/PP-26 (5 wt%) blend and the blend after 4 months in buffer (pH = 7).

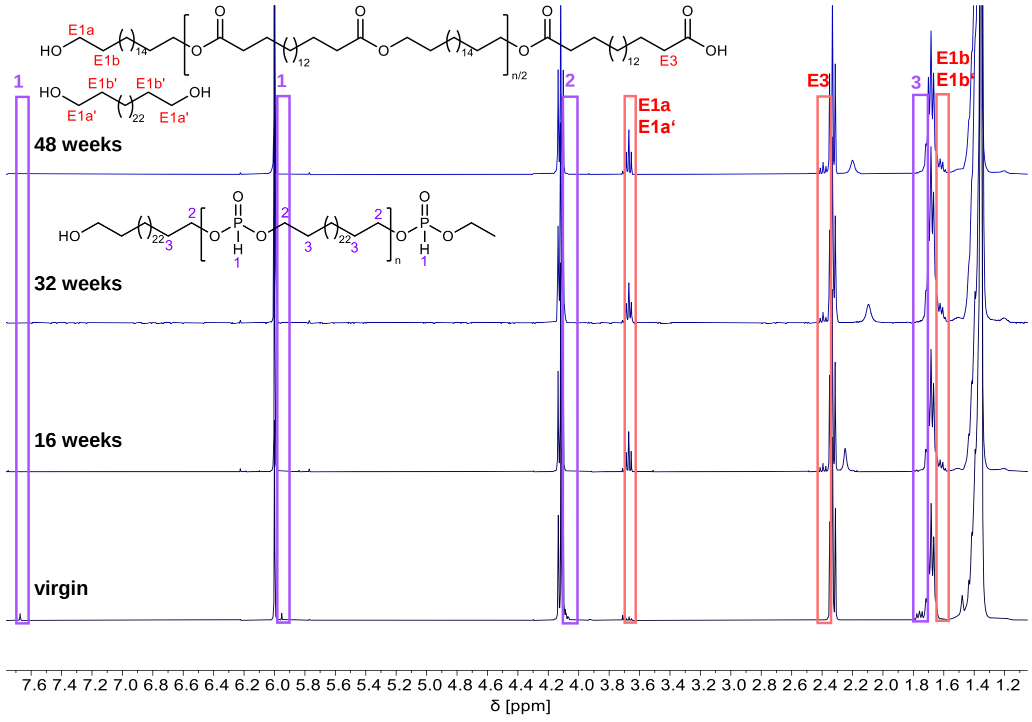

Figure S 76. Stacked ^{1}H NMR spectra (400 MHz, $C_2D_2Cl_4$, 383 K) of the virgin PE-18.18/PP-26 (10 wt%) blend and the blend after 4, 8 and 12 months in buffer (pH = 7). The purple markers highlight the PP-26 resonances disappearing upon hydrolysis while the red markers highlight the appearing or intensifying resonances stemming from the hydrolysis products.

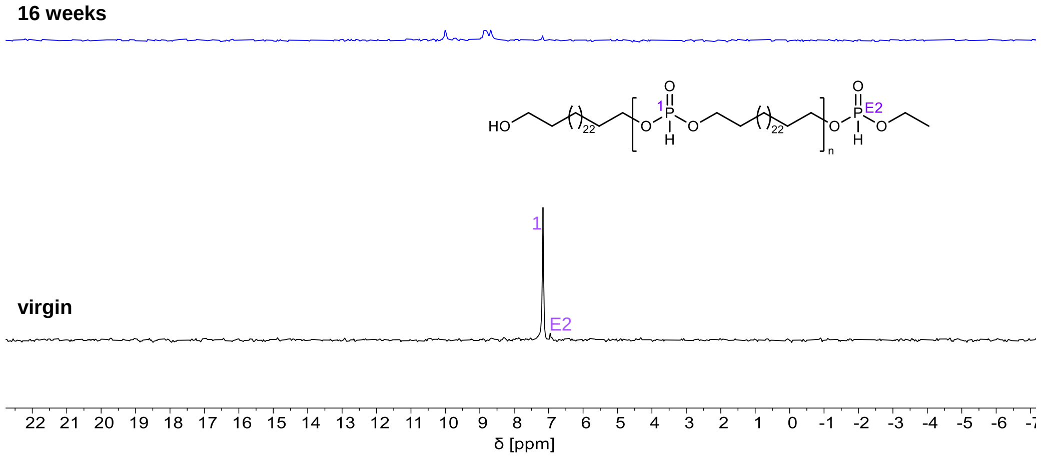

Figure S 77. Stacked $^{31}P\{^{1}H\}$ NMR spectra (162 MHz, $C_2D_2Cl_4$, 383 K) of the virgin PE-18.18/PP-26 (10 wt%) blend and the blend after 4 months in buffer (pH = 7).

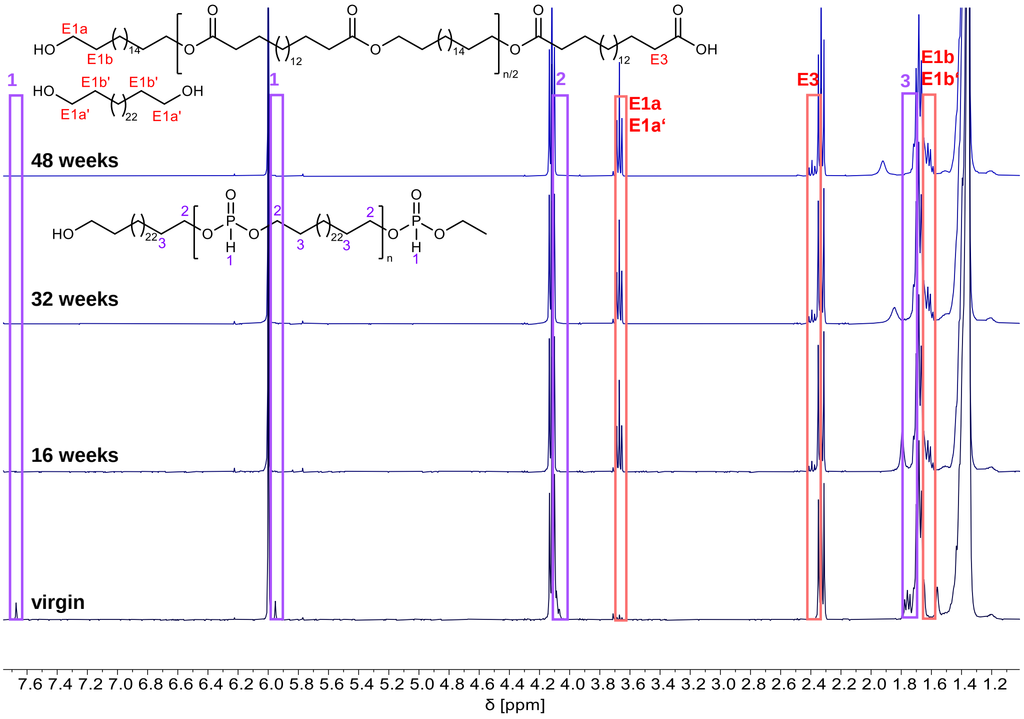

Figure S 78. Stacked ^{1}H NMR spectra (400 MHz, $C_2D_2Cl_4$, 383 K) of the virgin PE-18.18/PP-26 (20 wt%) blend and the blend after 4, 8 and 12 months in buffer (pH = 7). The purple markers highlight the PP-26 resonances disappearing upon hydrolysis while the red markers highlight the appearing or intensifying resonances stemming from the hydrolysis products.

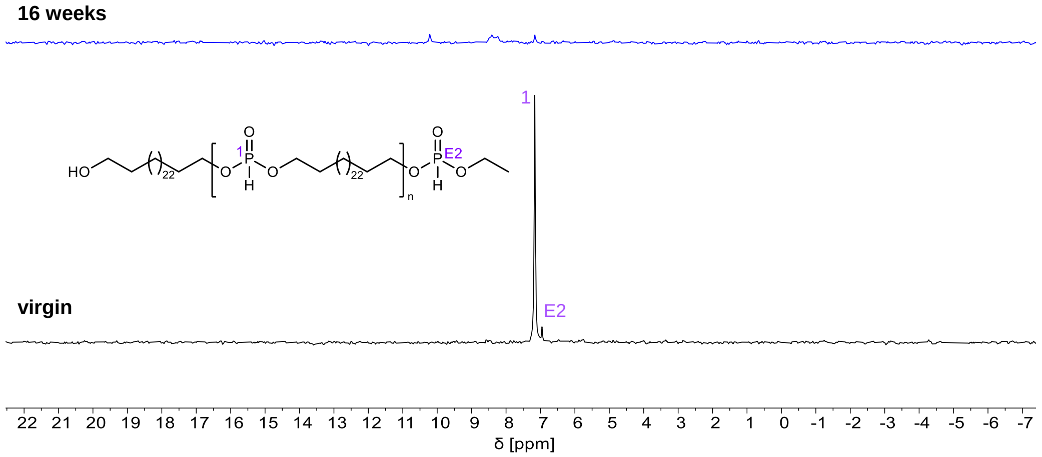

Figure S 79. Stacked $^{31}P\{^{1}H\}$ NMR spectra (162 MHz, $C_2D_2Cl_4$, 383 K) of the virgin PE-18.18/PP-26 (20 wt%) blend and the blend after 4 months in buffer (pH = 7).

NMR analysis of the PE-18.18/PP-18 blends exposed to hydrolysis media

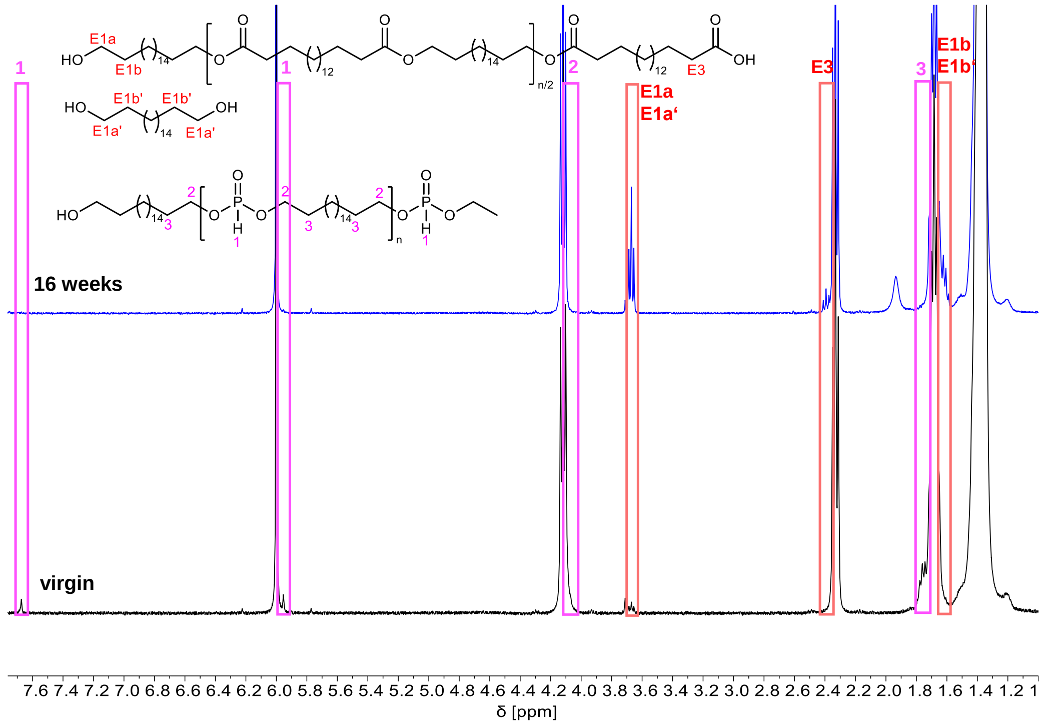

Figure S 80. Stacked ^{1}H NMR spectra (400 MHz, $C_2D_2Cl_4$, 383 K) of the virgin PE-18.18/PP-18 (10 wt%) blend and the blend after 4 months in buffer (pH = 8). The pink markers highlight the PP-18 resonances disappearing upon hydrolysis while the red markers highlight the appearing or intensifying resonances stemming from the hydrolysis products.

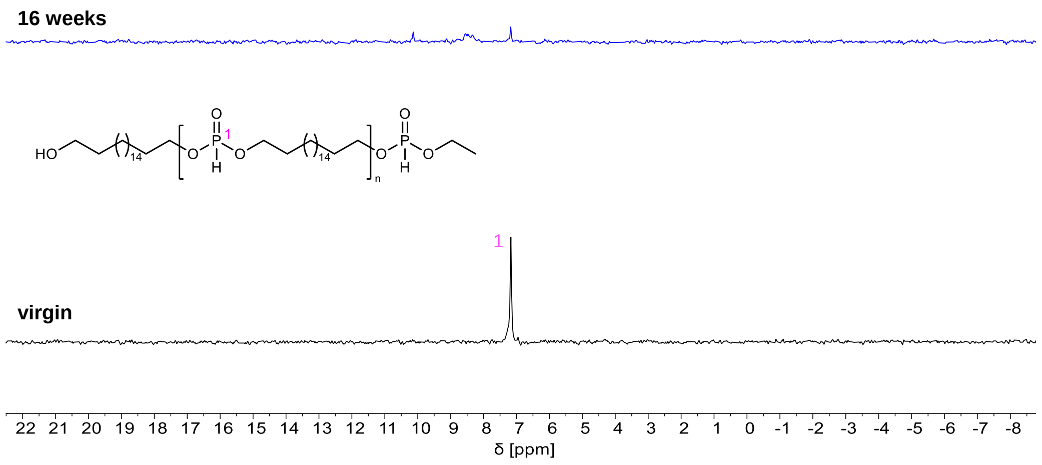

Figure S 81. Stacked $^{31}P\{^{1}H\}$ NMR spectra (162 MHz, $C_2D_2Cl_4$, 383 K) of the virgin PE-18.18/PP-18 (10 wt%) blend and the blend after 4 months in buffer (pH = 8).

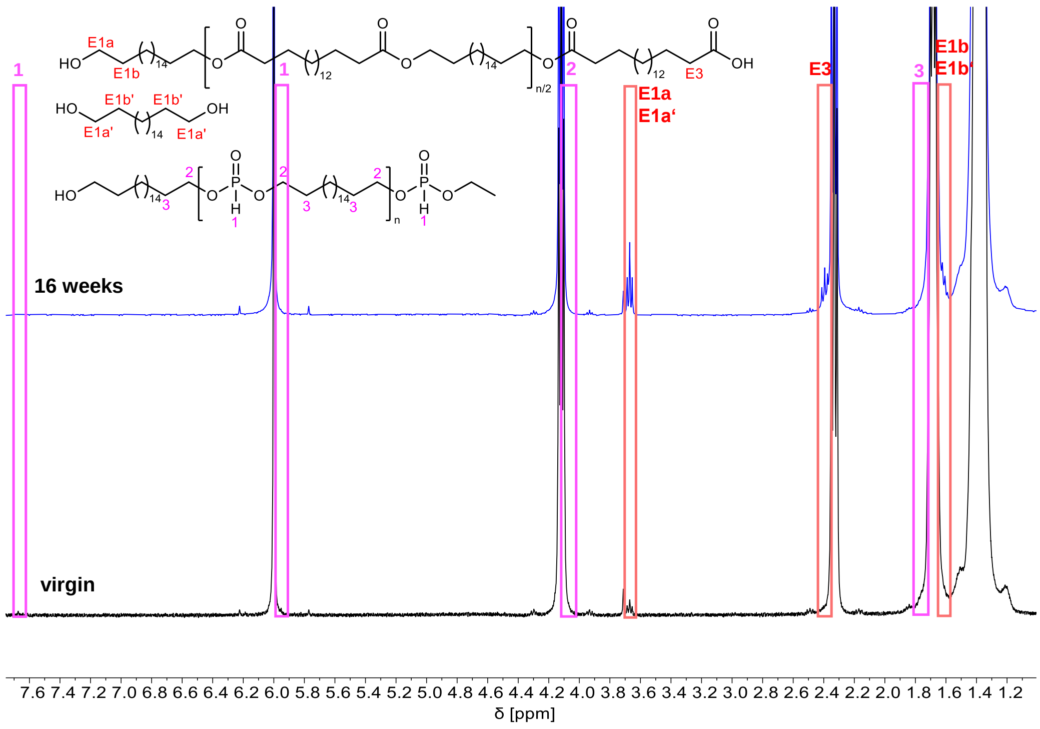

Figure S 82. Stacked ^{1}H NMR spectra (400 MHz, $C_2D_2Cl_4$, 383 K) of the virgin PE-18.18/PP-18 (2 wt%) blend and the blend after 4 months in buffer (pH = 8). The pink markers highlight the PP-18 resonances disappearing upon hydrolysis while the red markers highlight the appearing or intensifying resonances stemming from the hydrolysis products.

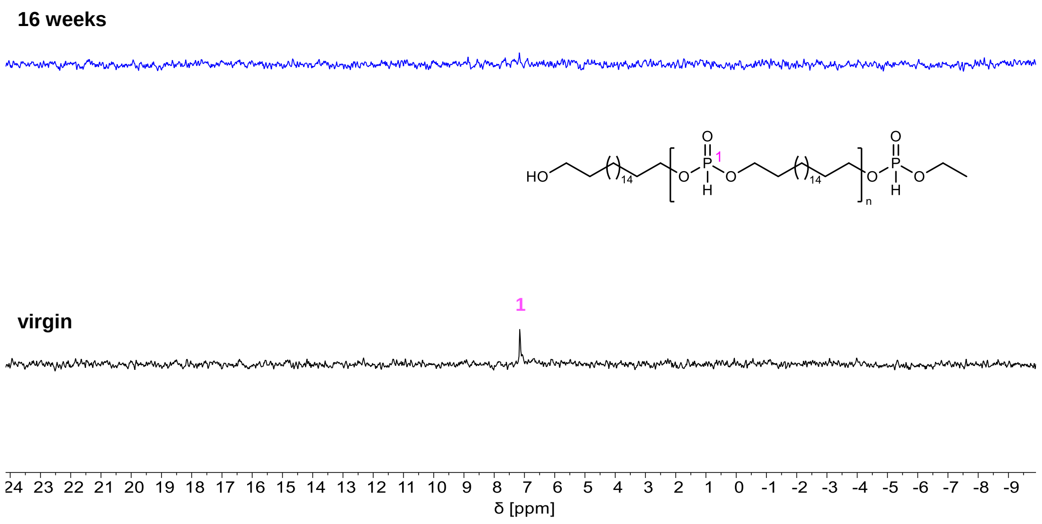

Figure S 83. Stacked $^{31}P\{^{1}H\}$ NMR spectra (162 MHz, $C_2D_2Cl_4$, 383 K) of the virgin PE-18.18/PP-18 (2 wt%) blend and the blend after 4 months in buffer (pH = 8).

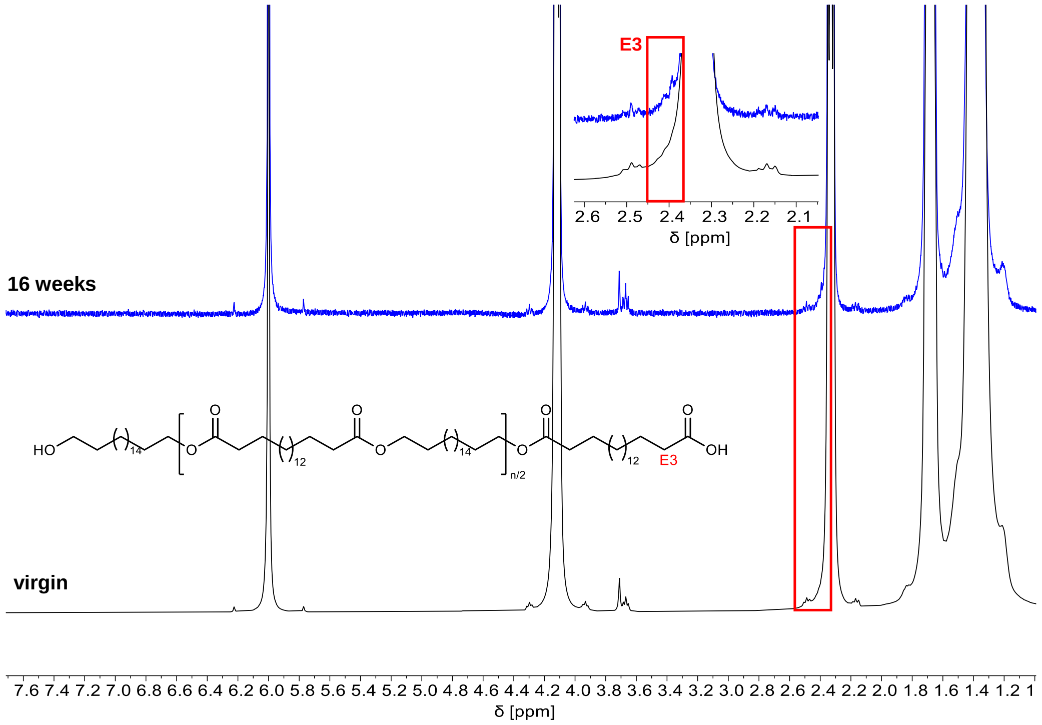

Figure S 84. Stacked ^{1}H NMR spectra (400 MHz, $C_2D_2Cl_4$, 383 K) of the virgin PE-18.18/PP-18 (0.5 wt%) blend and the blend after 4 months in buffer (pH = 8). The enlarged excerpt shows the carboxylic acid end group resonance E3 (δ = 2.39 ppm) resulting from the hydrolysis of the PE-18.18 blend component.

Estimation of the molecular weights of the PE-18.18 component of PE-18.18/PP-18/26 blends exposed to hydrolysis media by ^{1}H NMR

The degree of polymerization DP_n of hydrolyzed pure PE-18.18 which in addition to the hydroxy and methylester end groups, E1 and E2, also exhibits carboxylic acid end groups E3 can be calculated by the following equation:

Equation S 5.

$$DP_n = \frac{\int B1}{\frac{\int E2}{3}+\frac{\int E1}{2}+\frac{\int E3}{2}} + 1$$

Blended with a long-chain poly(H-phosphonate), **Equation S 5** cannot be used to determine the DP_n of the hydrolyzed PE-18.18 blend component. The PE-18.18/PP-18/26 blends contain additional 1,18-octadecanediol or 1,26-hexacosanediol with the end group E1' stemming from the hydrolyzed poly(H-phosphonate) component (cf. **Figure S 85**). Therefore, using **Equation S 5** would result in an underestimation of the degree of polymerization DP_n due to an overestimation of the integral of the hydroxy end groups E1.

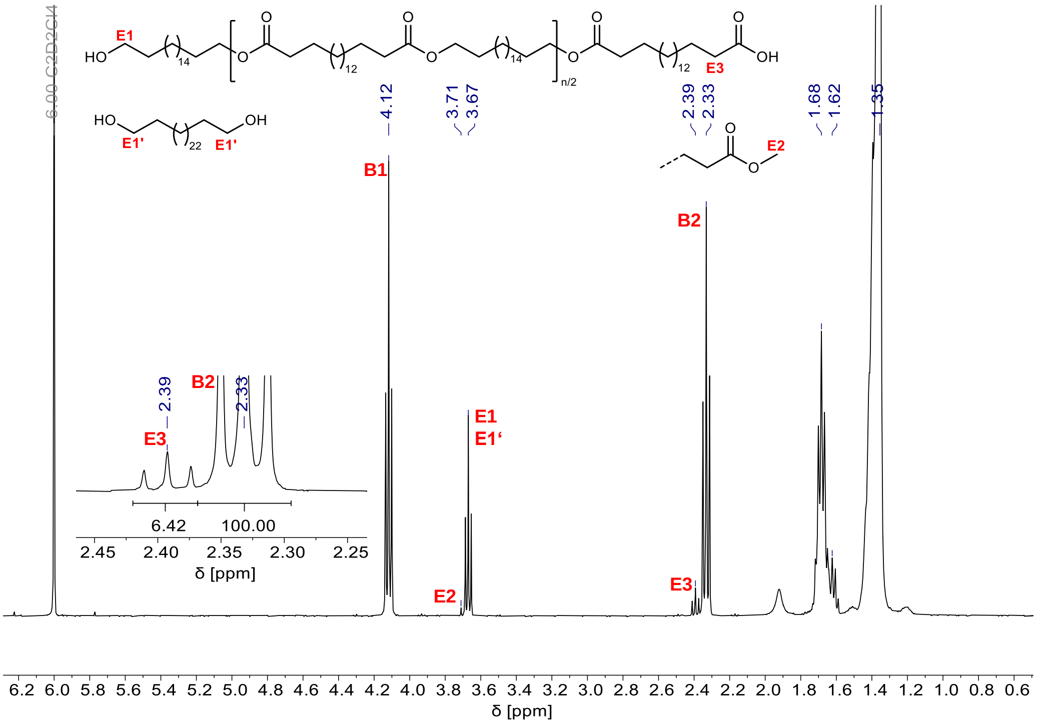

Figure S 85. ^{1}H NMR spectrum (400 MHz, $C_2D_2Cl_4$, 383 K) of PE-18.18 blended with 20 wt% PP-26 after 12 months in buffer (pH = 7). The enlarged excerpt shows the carboxylic acid end group resonance E3 (δ = 2.39 ppm) and the backbone resonance B2 (δ = 2.33 ppm).

The application of the following approximations yields **Equation S 6** which is independent of the integral of the hydroxy end groups E1: a) $\int E2 \approx 0$; b) $\int E1 \approx \int E3$; c) $\int B1 = \int B2$.

Equation S 6.

$$DP_n \approx \frac{\int B2}{\int E3} + 1$$

Equation S 6 facilitates the estimation of the DP_n of the PE-18.18 blend component of hydrolyzed PE-18.18/poly(H-phosphonate) blends by integration of the backbone resonance B2 and the carboxylic acid end group resonance E3 as shown in the enlarged excerpt in **Figure S 85**.

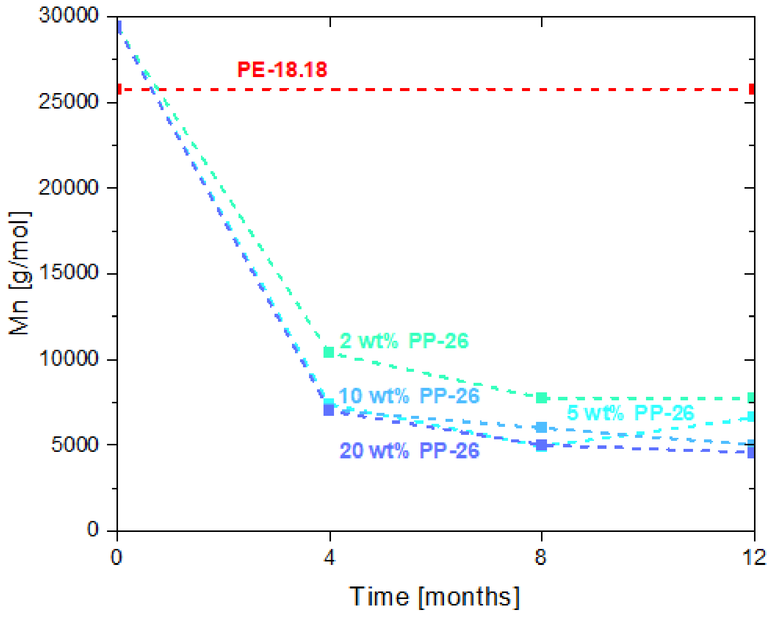

Figure S 86. Decrease of the number average molecular weight M_n of the PE-18.18 component of different PE-18.18/Poly(H-phosphonate)-26 blends over time. The blend samples were stored in buffer solution (pH = 7) at 25 °C. M_n was determined by ^{1}H NMR end group analysis employing **Equation S 6** and **Equation S 8**. The resonances B2 and E3 were deconvoluted to improve integration. Pure PE-18.18 stored under the same conditions is shown for comparison. Connecting lines are a guide to the eye.

Determination of the molecular weights of PE-18.18/PP-26 blends exposed to hydrolysis media by GPC

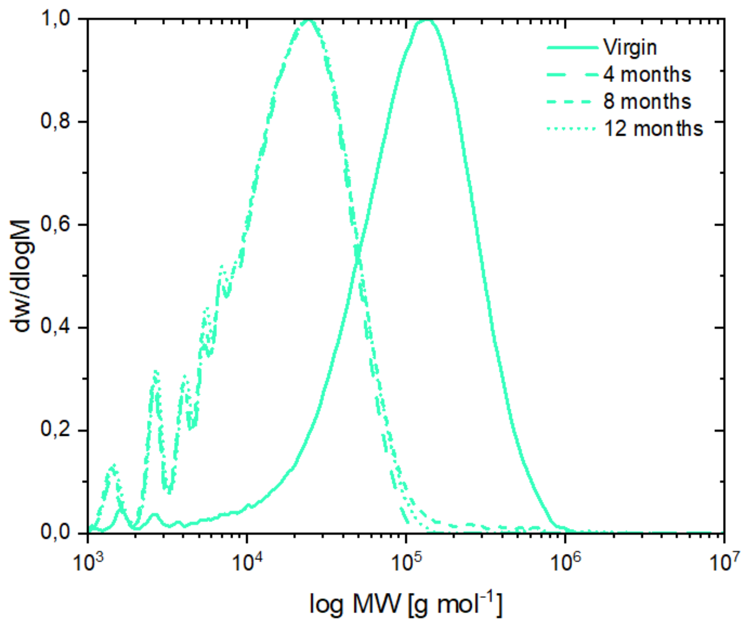

Figure S 87. GPC traces of injection molded PE-18.18/PP-26 (2 wt%) blend samples stored in buffer solution (pH = 7) at 25 °C for 4, 8 and 12 months. The GPC trace of the virgin blend is shown for comparison.

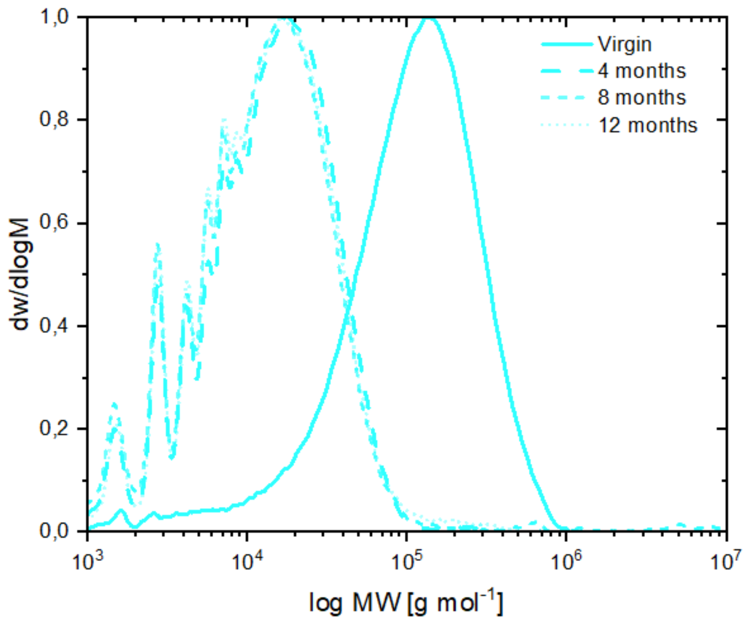

Figure S 88. GPC traces of injection molded PE-18.18/PP-26 (5 wt%) blend samples stored in buffer solution (pH = 7) at 25 °C for 4, 8 and 12 months. The GPC trace of the virgin blend is shown for comparison.

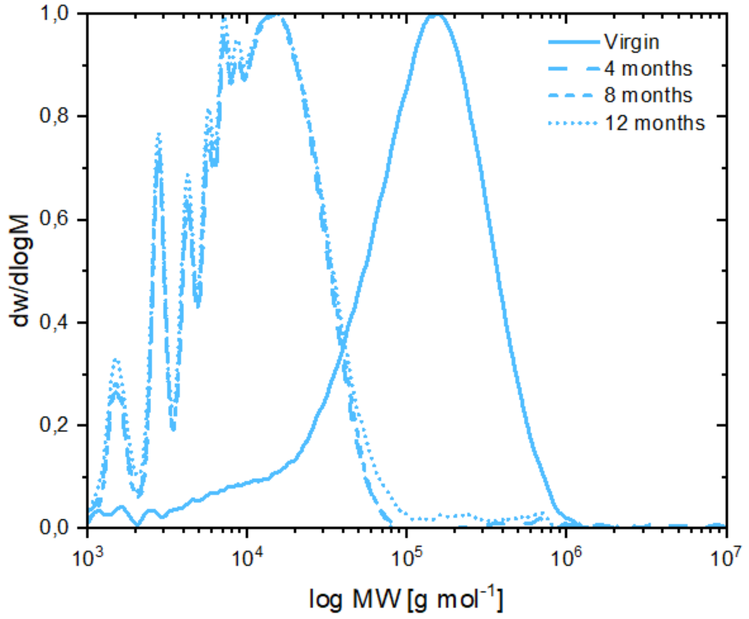

Figure S 89. GPC traces of injection molded PE-18.18/PP-26 (10 wt%) blend samples stored in buffer solution (pH = 7) at 25 °C for 4, 8 and 12 months. The GPC trace of the virgin blend is shown for comparison.

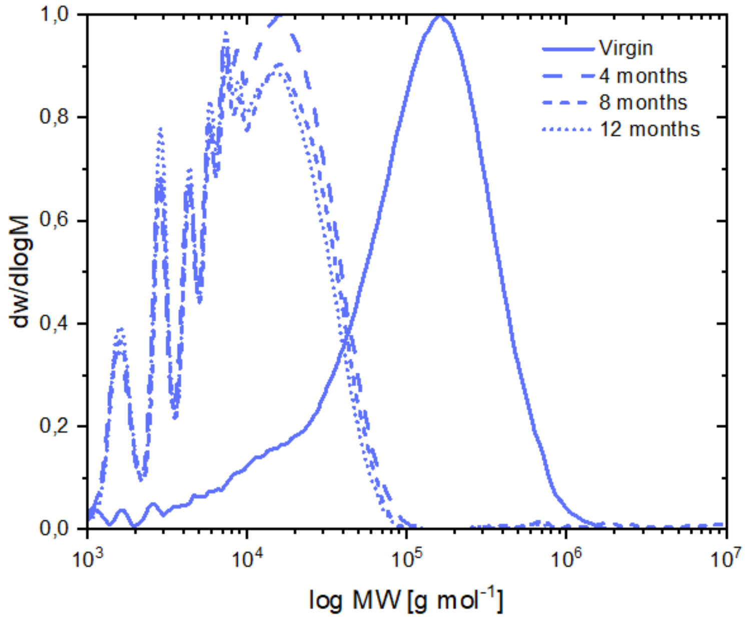

Figure S 90. GPC traces of injection molded PE-18.18/PP-26 (20 wt%) blend samples stored in buffer solution (pH = 7) at 25 °C for 4, 8 and 12 months. The GPC trace of the virgin blend is shown for comparison.

Determination of the molecular weights of PE-18.18/PP-18 blends exposed to hydrolysis media by GPC

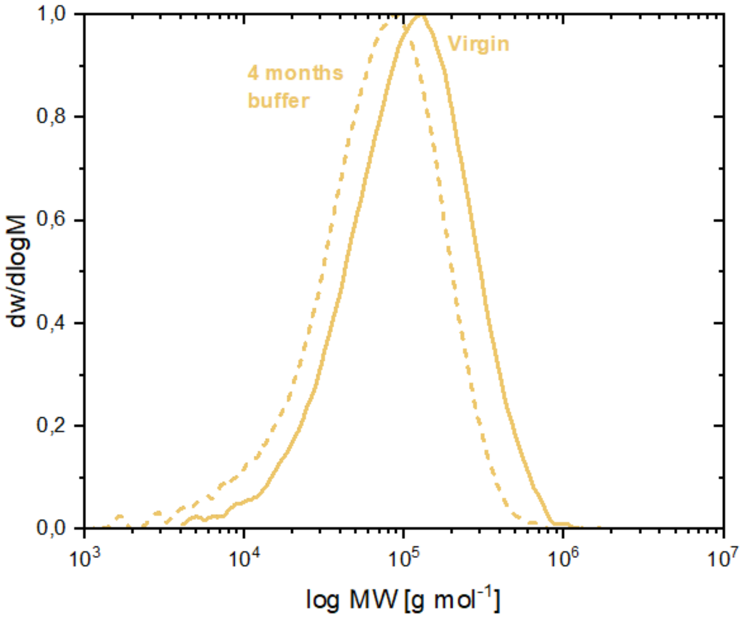

Figure S 91. GPC traces of injection molded PE-18.18/PP-18 (0.5 wt%) blend samples stored in buffer solution (pH = 8) at 25 °C for 4 months. The GPC trace of the virgin blend is shown for comparison.

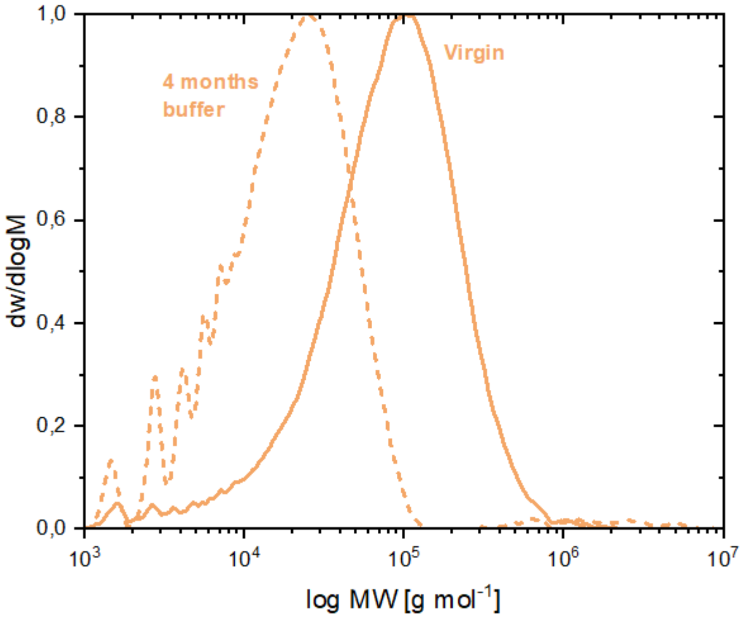

Figure S 92. GPC traces of injection molded PE-18.18/PP-18 (2 wt%) blend samples stored in buffer solution (pH = 8) at 25 °C for 4 months. The GPC trace of the virgin blend is shown for comparison.

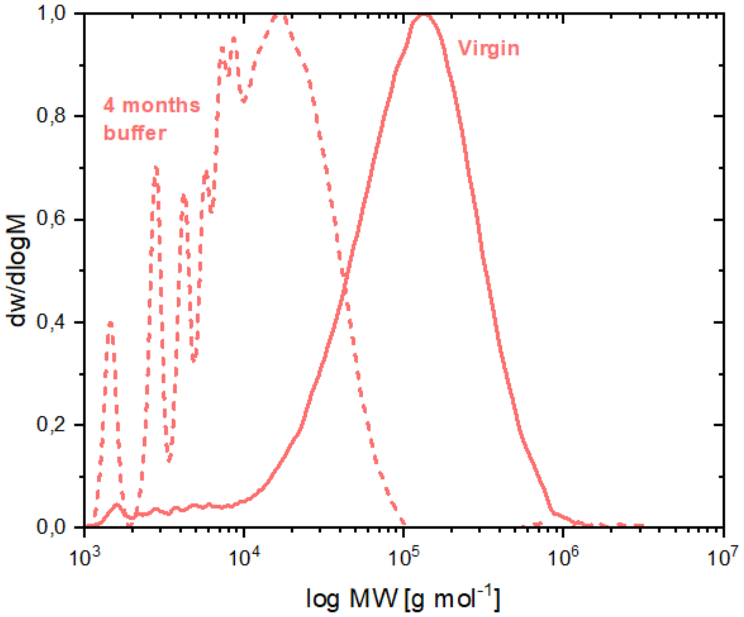

Figure S 93. GPC traces of injection molded PE-18.18/PP-18 (10 wt%) blend samples stored in buffer solution (pH = 8) at 25 °C for 4 months. The GPC trace of the virgin blend is shown for comparison.

WAXS analysis of the PE-18.18/PP-26 blend specimens exposed to hydrolysis media

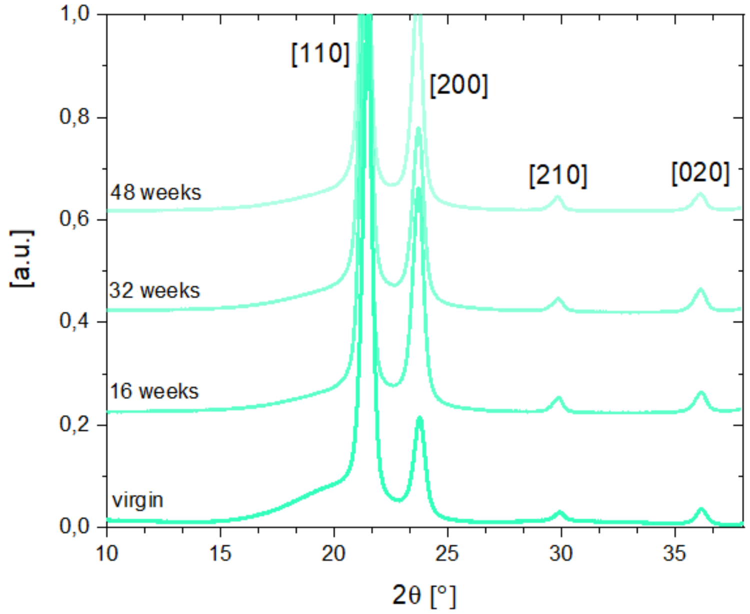

Figure S 94. WAXS diffractograms of injection molded PE-18.18/PP-26 (2 wt%) blend samples stored in buffer solution (pH = 7) at 25 °C for 4, 8 and 12 months. The WAXS diffractogram of the virgin blend is shown for comparison.

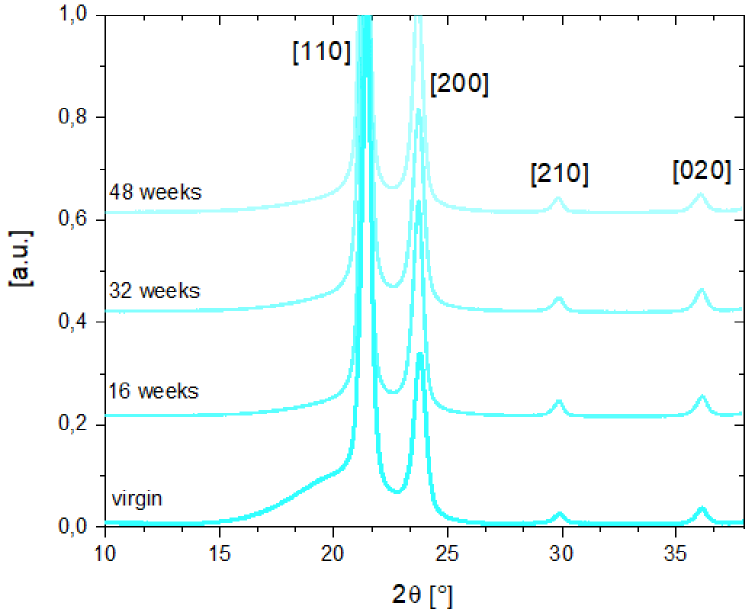

Figure S 95. WAXS diffractograms of injection molded PE-18.18/PP-26 (5 wt%) blend samples stored in buffer solution (pH = 7) at 25 °C for 4, 8 and 12 months. The WAXS diffractogram of the virgin blend is shown for comparison.

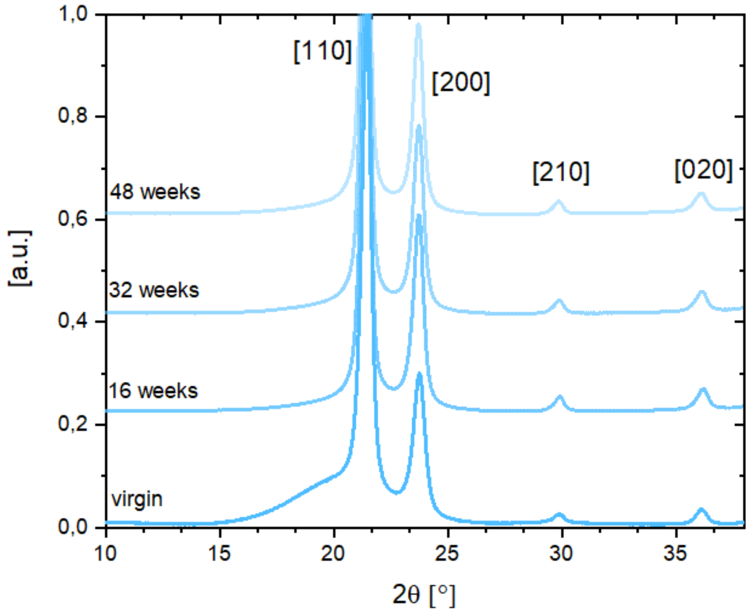

Figure S 96. WAXS diffractograms of injection molded PE-18.18/PP-26 (10 wt%) blend samples stored in buffer solution (pH = 7) at 25 °C for 4, 8 and 12 months. The WAXS diffractogram of the virgin blend is shown for comparison.

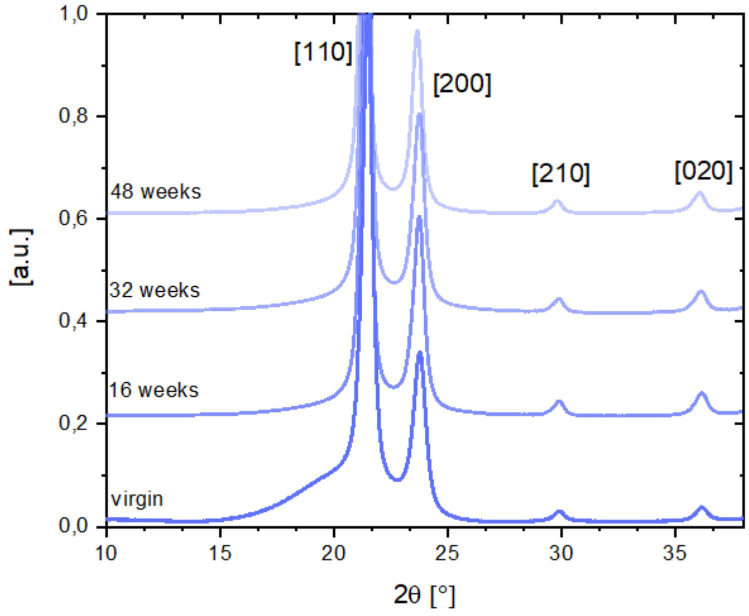

Figure S 97. WAXS diffractograms of injection molded PE-18.18/PP-26 (20 wt%) blend samples stored in buffer solution (pH = 7) at 25 °C for 4, 8 and 12 months. The WAXS diffractogram of the virgin blend is shown for comparison.

WAXS analysis of the PE-18.18/PP-18 blend specimens exposed to hydrolysis media

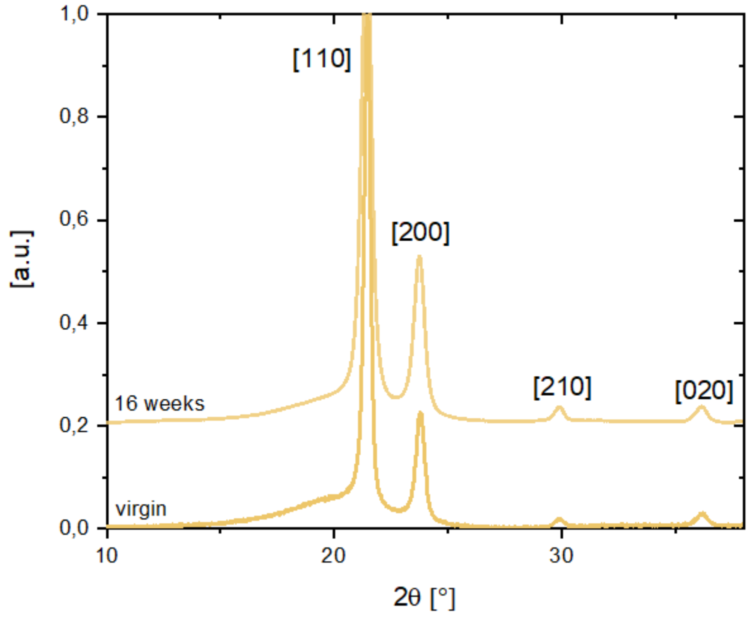

Figure S 98. WAXS diffractogram of an injection molded PE-18.18/PP-18 (0.5 wt%) blend sample stored in buffer solution (pH = 8) at 25 °C for 4 months. The WAXS diffractogram of the virgin blend is shown for comparison.

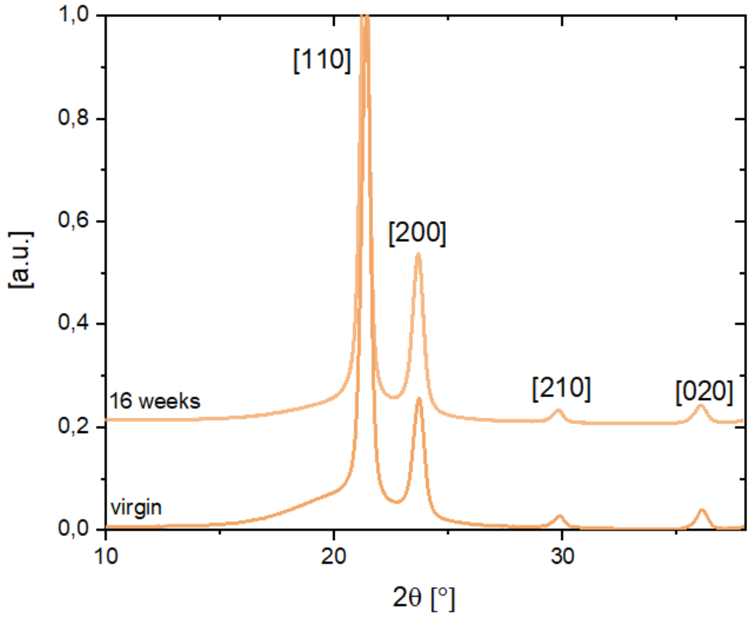

Figure S 99. WAXS diffractogram of an injection molded PE-18.18/PP-18 (2 wt%) blend sample stored in buffer solution (pH = 8) at 25 °C for 4 months. The WAXS diffractogram of the virgin blend is shown for comparison.

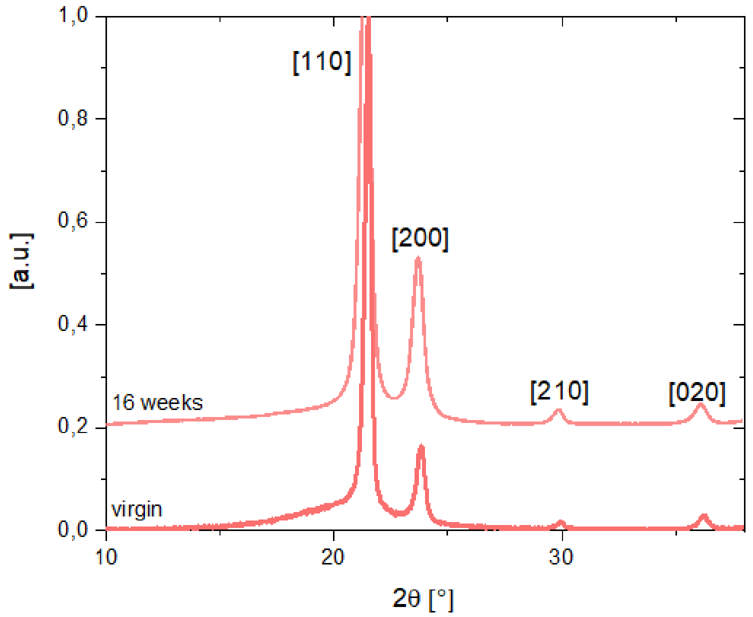

Figure S 100. WAXS diffractogram of an injection molded PE-18.18/PP-18 (10 wt%) blend sample stored in buffer solution (pH = 8) at 25 °C for 4 months. The WAXS diffractogram of the virgin blend is shown for comparison.

Analysis of injection molded PE-18.18 specimens exposed to hydrolysis media

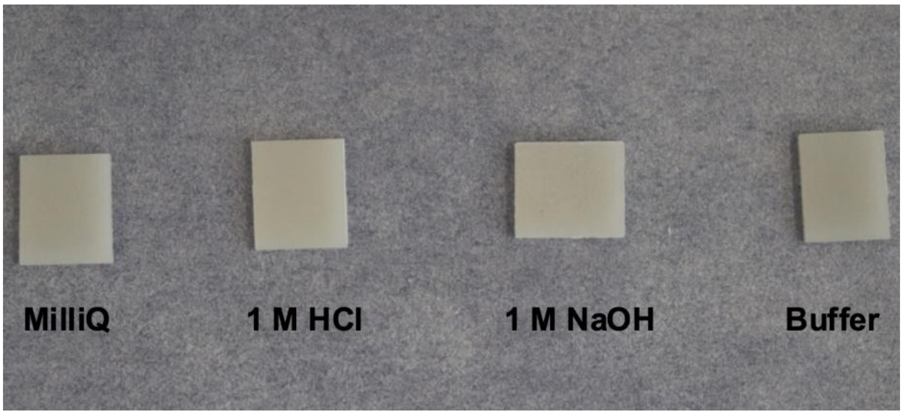

Figure S 101. Injection molded PE-18.18 specimens after 12 months in different media at 25 °C. The weight change of the samples amounted to 0.04 %, 0.07 %, 0.18 % and 0.01 % for the MilliQ water, the 1 M HCl, the 1 M NaOH and the buffer (pH = 7), respectively.

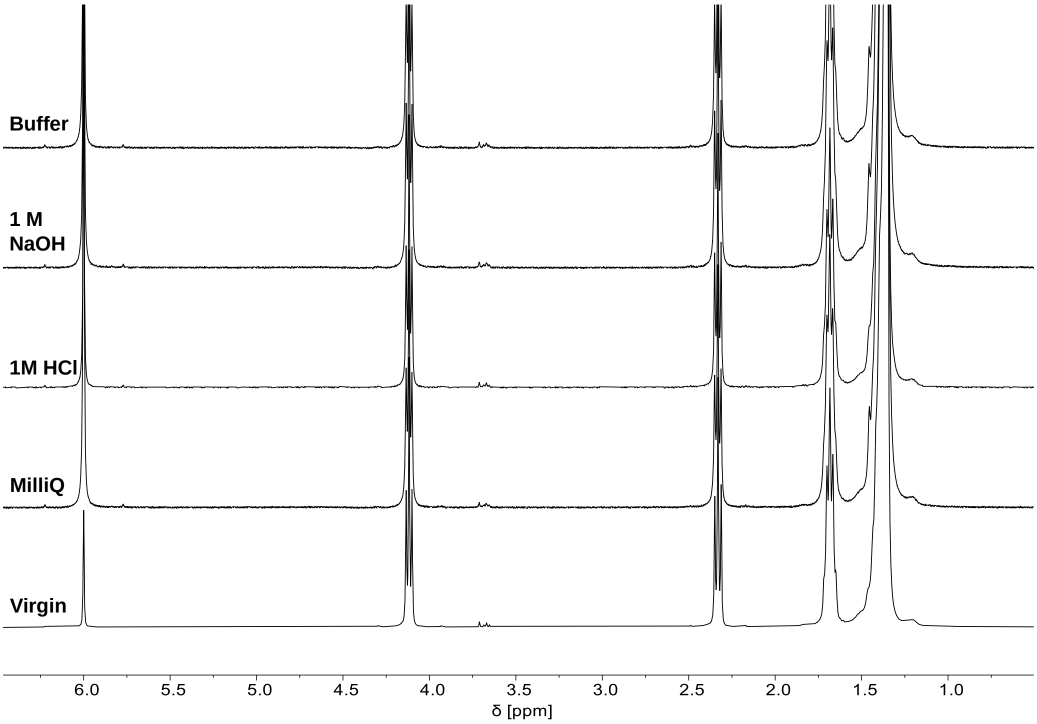

Figure S 102. Stacked ^1H NMR spectra (400 MHz, $C_2D_2Cl_4$, 383 K) of virgin PE-18.18 and injection molded specimens after 12 months in different media.[1]

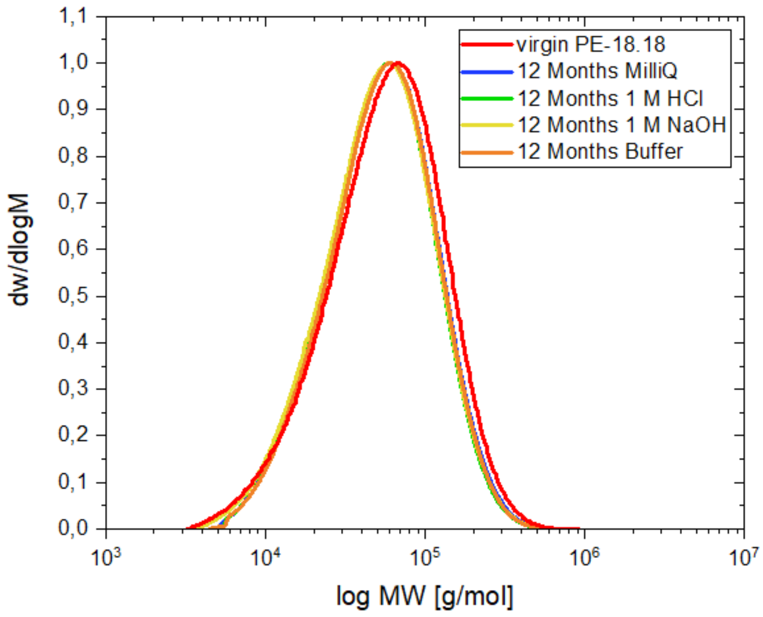

Figure S 103. HT-GPC traces of virgin PE-18.18 and injection molded specimens after 12 months in different media (cf. **Figure S 121** for details on measurement parameters).[1]

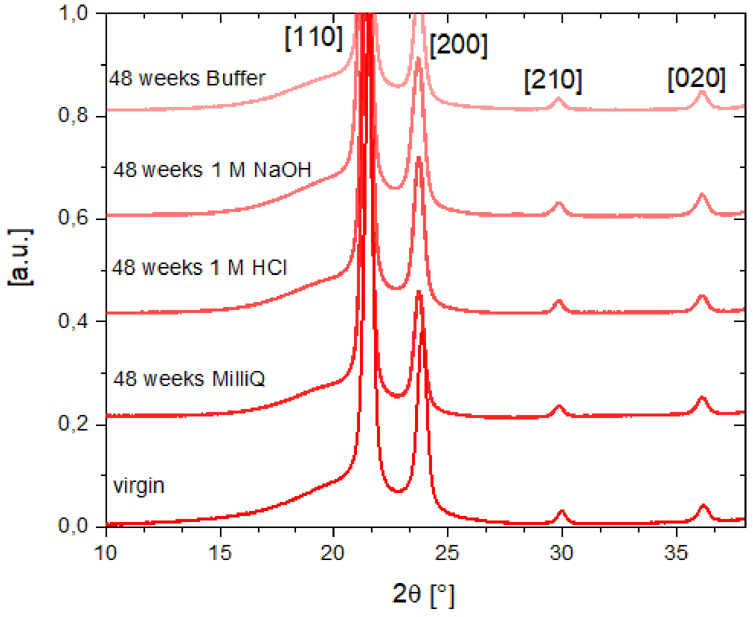

Figure S 104. WAXS diffractograms of virgin PE-18.18 and injection molded specimens after 12 months in different media. The crystallinity amounts to 64 %, 73 %, 71 %, 72 %, and 70 % (from bottom to top).

Additional data for aqueous exposure of test bar specimens

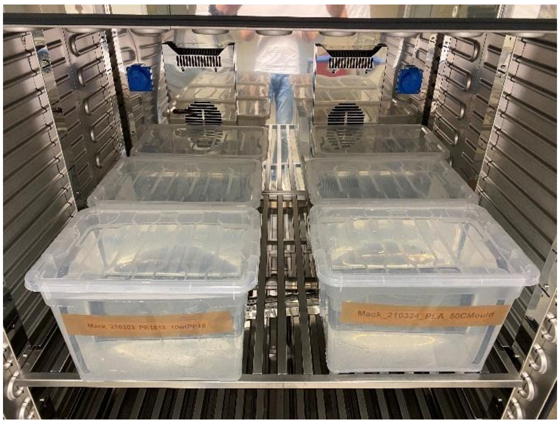

Figure S 105. Experimental setup of the aqueous exposure of test bar specimens experiments for PE-18.18, its PP-18 blends and commercial HDPE. The sealed boxes containing approx. 15 L of deionized water and 2 tensile test specimens are stored in a Peltier temperature-controlled cabinet.

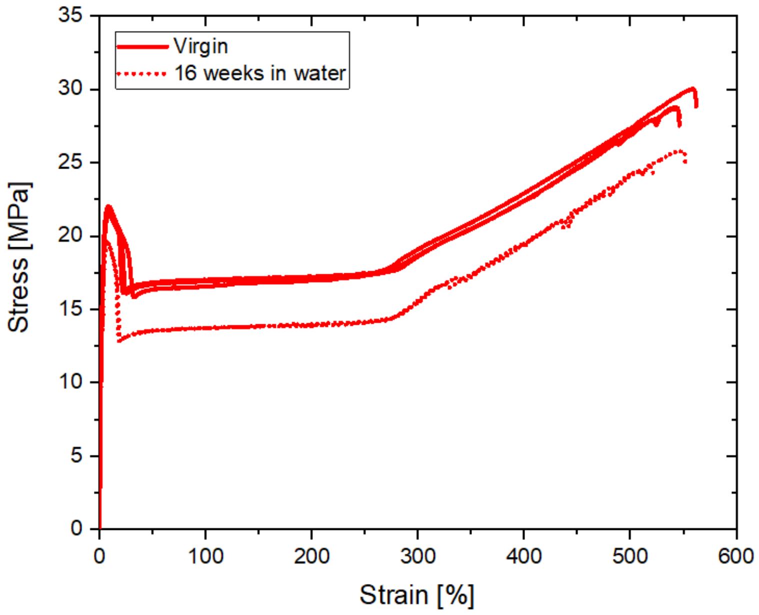

Figure S 106. Stress-strain curves of virgin PE-18.18 tensile test specimens and of two PE-18.18 tensile test specimens immersed in water for 4 months.

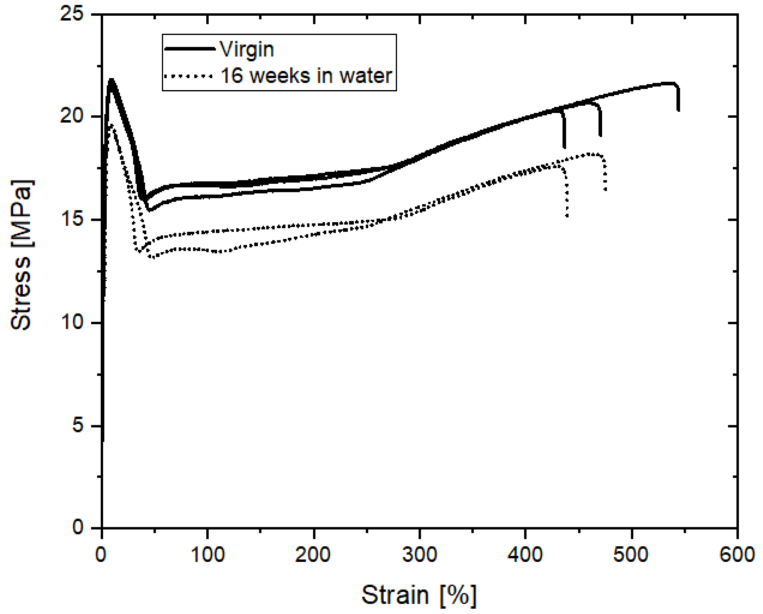

Figure S 107. Stress-strain curves of virgin HDPE tensile test specimens and of two HDPE tensile test specimens immersed in water for 4 months.

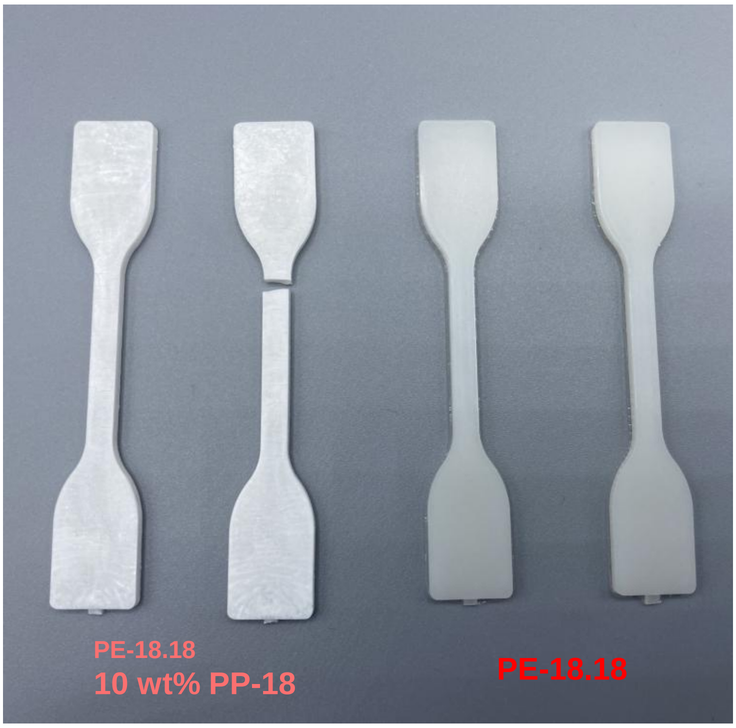

Figure S 108. Tensile test specimens of the PE-18.18/PP-18 (10 wt%) blend and of PE-18.18 immersed in water for 4 months at 25 °C. Upon storage in water, the PE-18.18/PP-18 (10 wt%) and the PE-18.18/PP-18 (2 wt%) blend specimens embrittled to an extent that tensile testing was not possible.

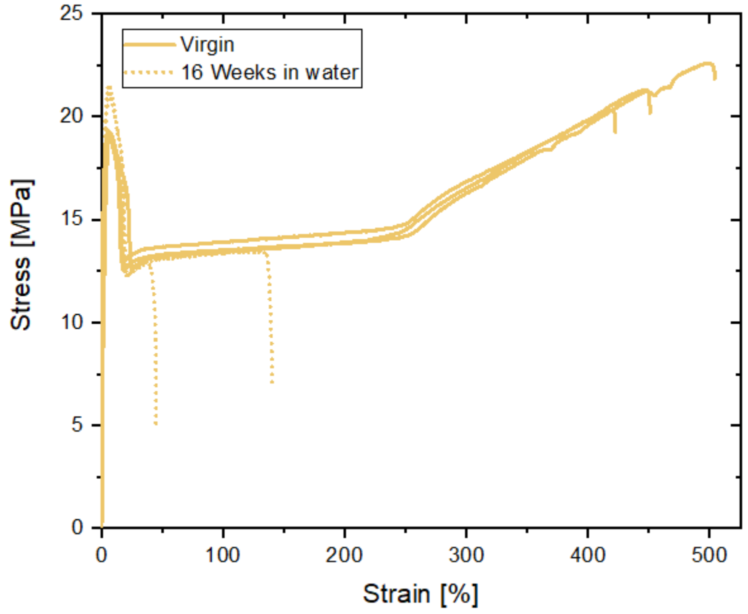

Figure S 109. Stress-strain curves of virgin PE-18.18/PP-18 (0.5 wt%) blend tensile test specimens and of two PE-18.18/PP-18 (0.5 wt%) tensile test specimens immersed in water for 4 months.

Additional data for stability of test bar specimens in air under ambient conditions

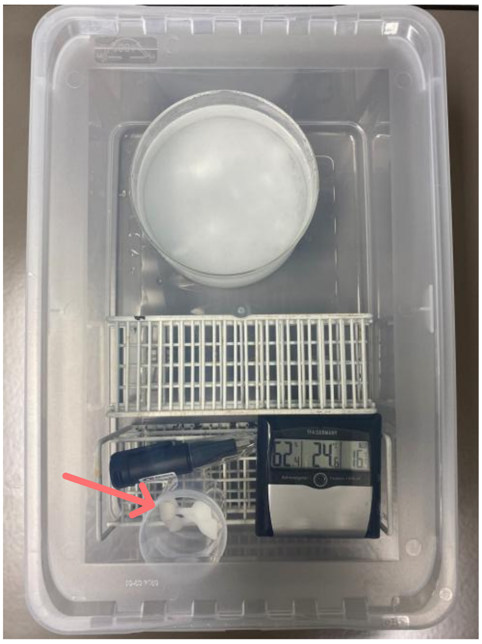

Figure S 110. Experimental setup of the stability of test bar specimens in air under ambient conditions experiments for PE-18.18, its PP-18 blends and commercial HDPE. The sealed chamber containing a supersaturated aqueous solution of NaBr, three tensile test specimens per material (marked with the red arrow) and a humidity sensor is stored in a Peltier temperature-controlled cabinet.

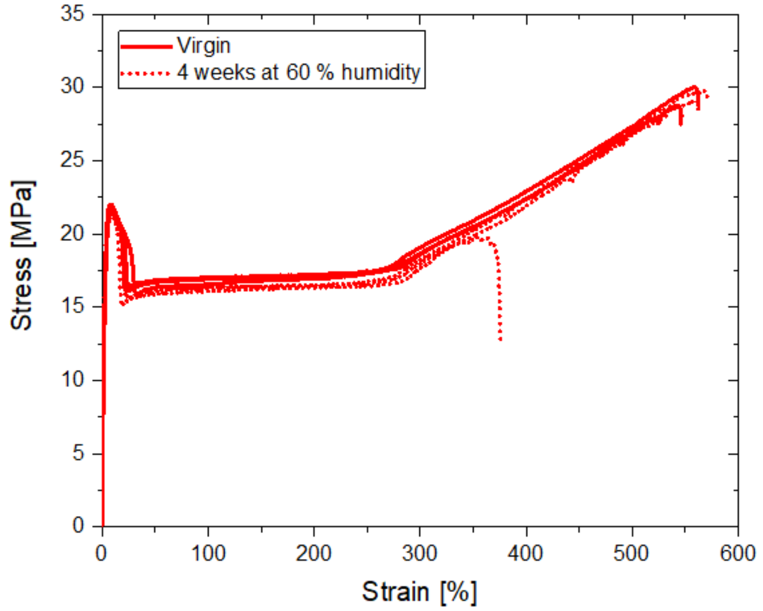

Figure S 111. Stress-strain curves of virgin PE-18.18 tensile test specimens and of three PE-18.18 tensile test specimens stored at 60 % humidity and 25 °C for 1 month.

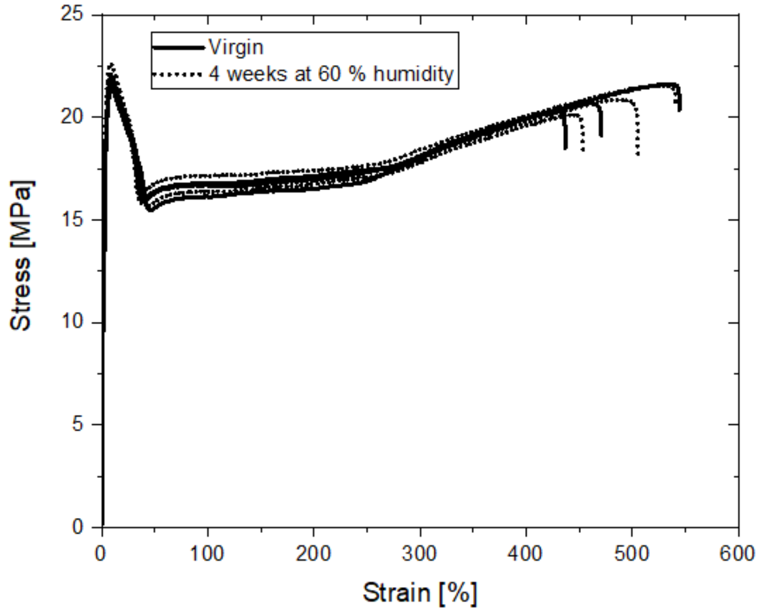

Figure S 112. Stress-strain curves of virgin HDPE tensile test specimens and of three HDPE tensile test specimens stored at 60 % humidity and 25 °C for 1 month.

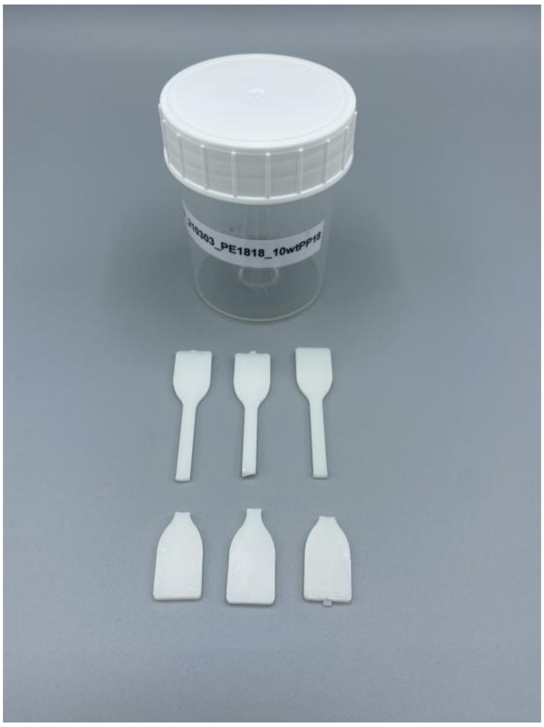

Figure S 113. Tensile test specimens of the PE-18.18/PP-18 (10 wt%) blend stored at 60 % humidity and 25 °C for 1 month. The blend specimens embrittled to an extent that tensile testing was not possible.

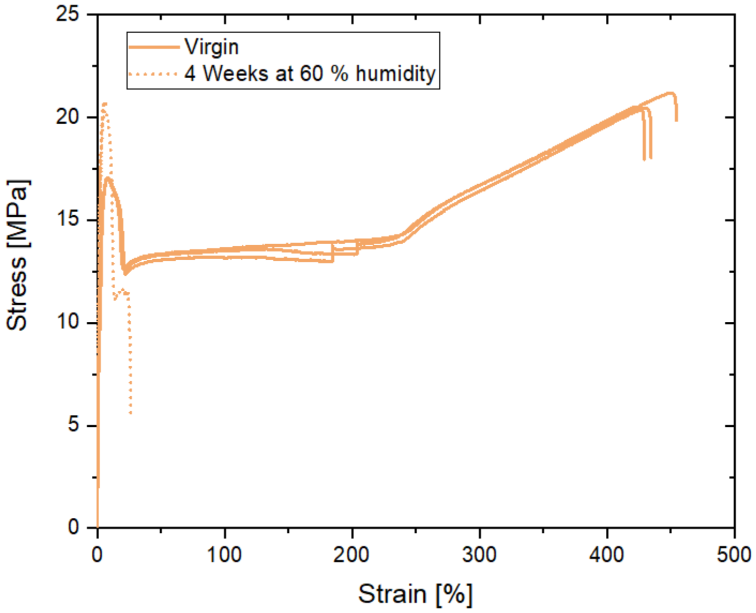

Figure S 114. Stress-strain curves of virgin PE-18.18/PP-18 (2 wt%) tensile test specimens and of three PE-18.18/PP-18 (2 wt%) tensile test specimens stored at 60 % humidity and 25 °C for 1 month.

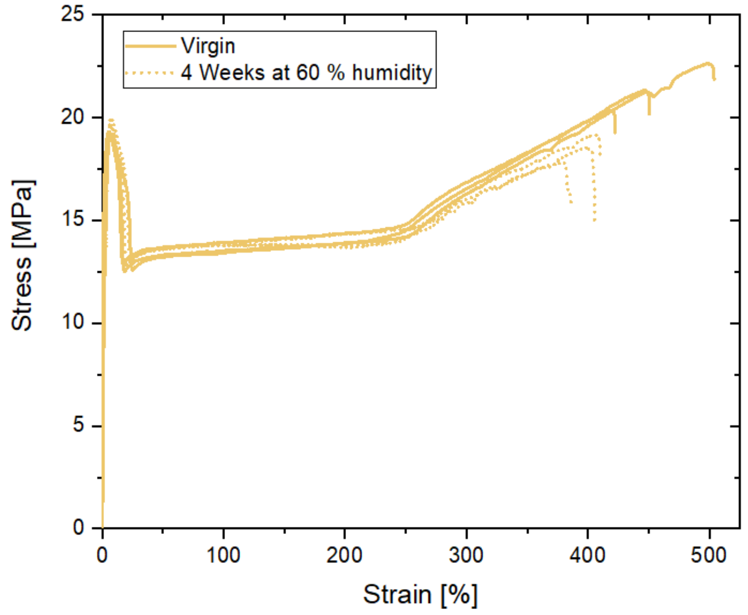

Figure S 115. Stress-strain curves of virgin PE-18.18/PP-18 (0.5 wt%) tensile test specimens and of three PE-18.18/PP-18 (0.5 wt%) tensile test specimens stored at 60 % humidity and 25 °C for 1 month.

Additional characterization data for 1,18-octadecanediol

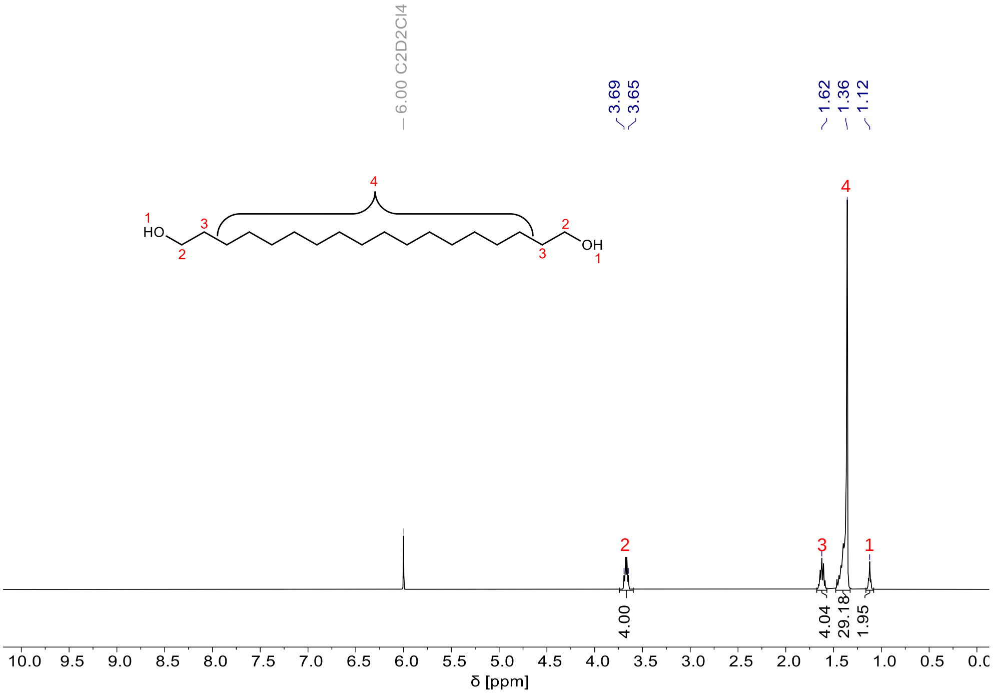

Figure S 116. ^{1}H NMR spectrum (400 MHz, $C_2D_2Cl_4$, 383 K) of 1,18-octadecanediol.

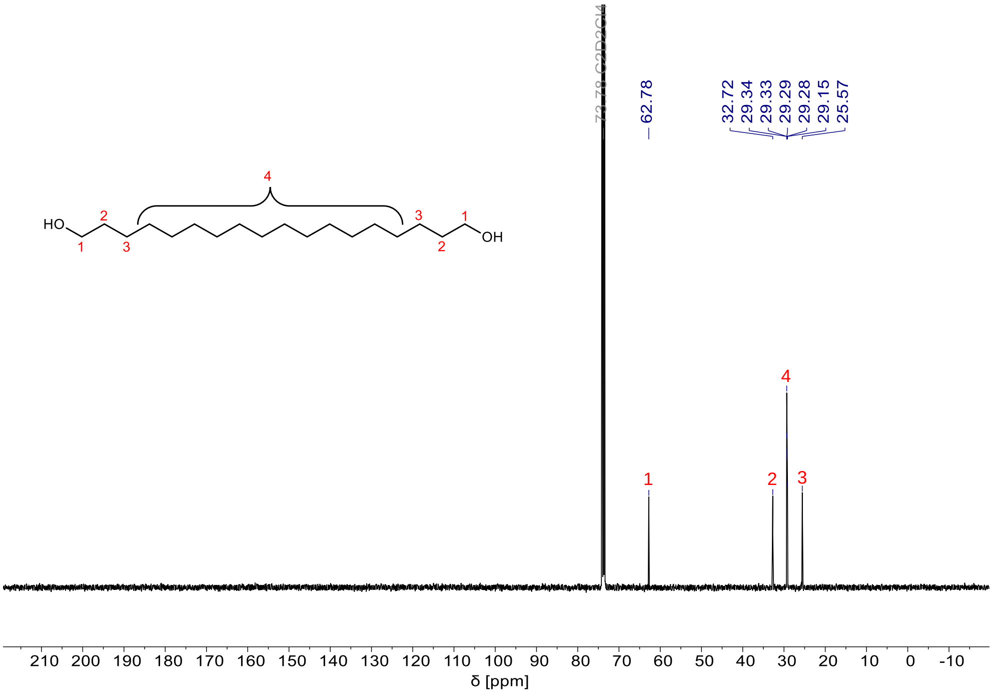

Figure S 117. ^{13}C{^{1}H} NMR spectrum (101 MHz, $C_2D_2Cl_4$, 383 K) of 1,18-octadecanediol.

Additional characterization data for 1,26-hexacosanediol

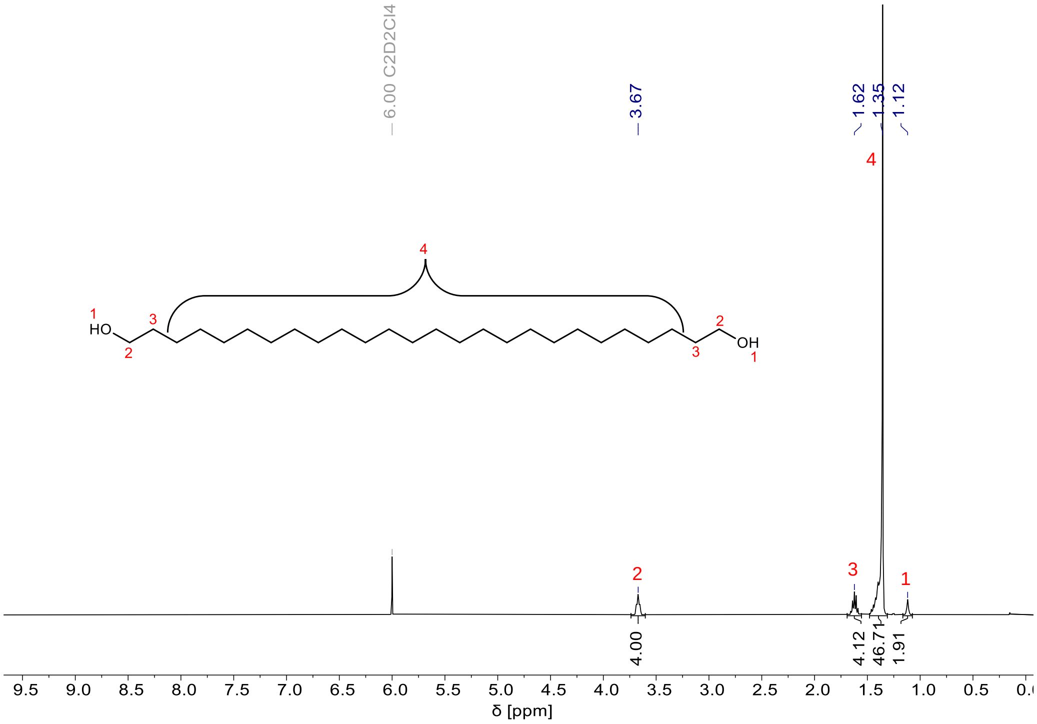

Figure S 118. ^{1}H NMR spectrum (400 MHz, $C_2D_2Cl_4$, 383 K) of 1,26-hexacosanediol.

Additional characterization data for PE-18.18

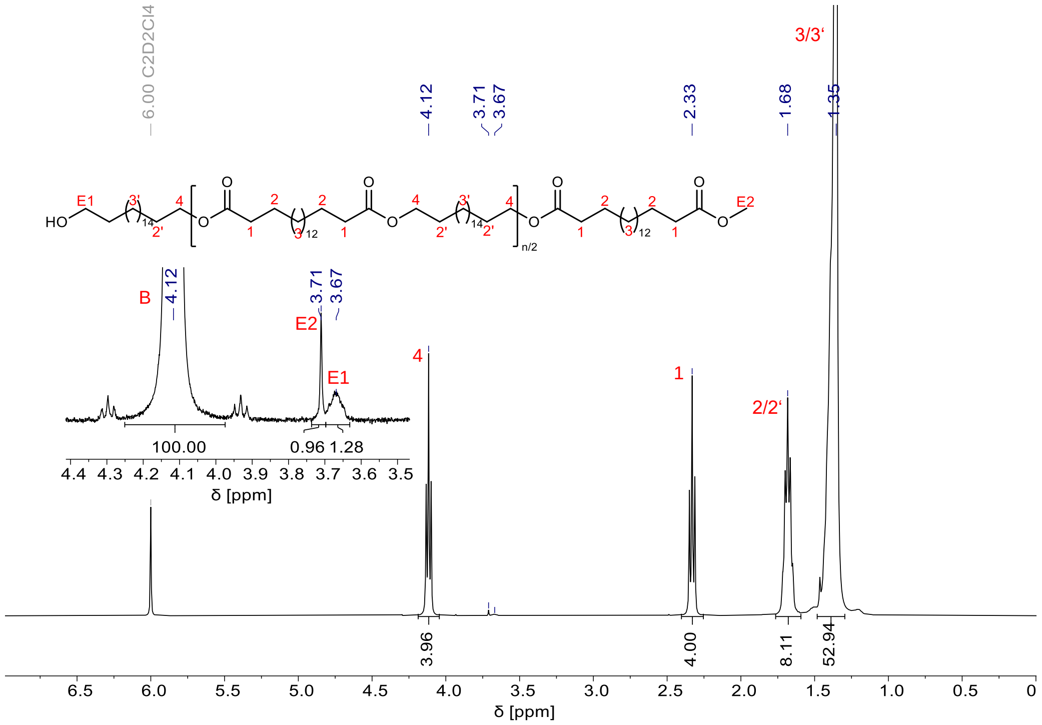

Figure S 119. ^{1}H NMR spectrum (400 MHz, $C_2D_2Cl_4$, 383 K) of PE-18.18 used for blending with PP-26. The enlarged excerpt shows the integrals of the backbone B and the hydroxy E1 and methyl ester E2 end group resonances used for the determination of the number average molecular weight (M_n = 30 kg/mol) by **Equations S 7** and **S 8**.

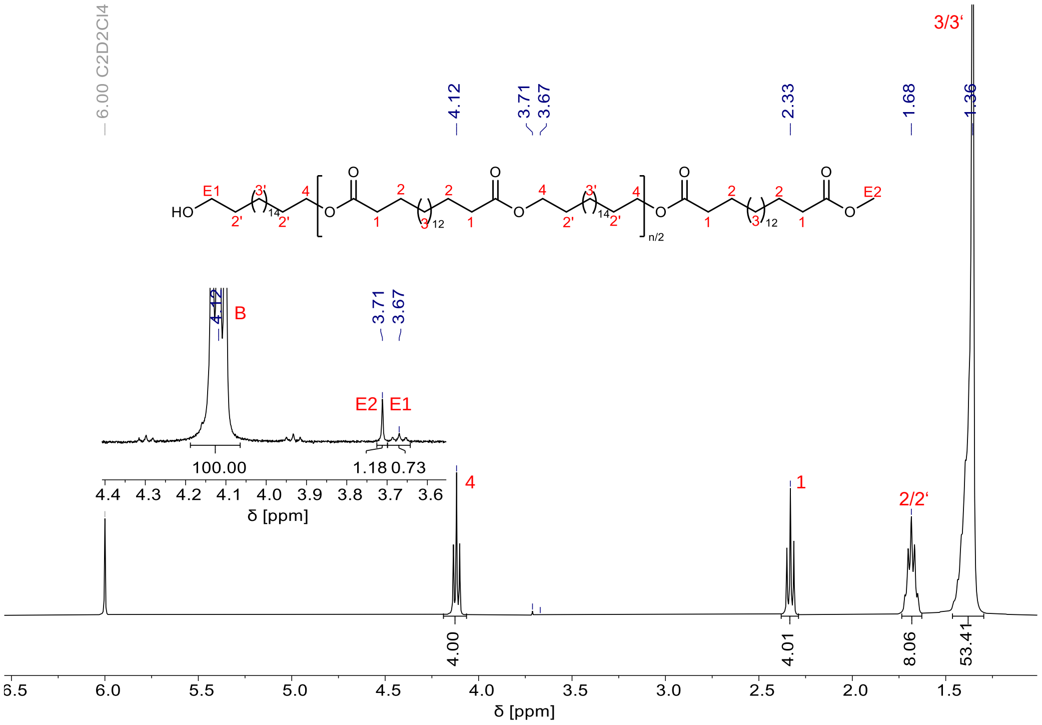

Figure S 120. ^{1}H-NMR spectrum (400 MHz, $C_2D_2Cl_4$, 383 K) of PE-18.18 used for blending with PP-18. The enlarged detail shows the integrals of the backbone B and the hydroxy E1 and methyl ester E2 end group resonances used for the determination of the number average molecular weight (M_n = 37 kg/mol) according to **Equations S 7** and **S 8**.

Equation S 7. $$DP_n = \frac{\int B}{\frac{\int E2}{3}+\frac{\int E1}{2}} + 1$$

Equation S 8. $$M_n = DP_n * 282\, g/mol$$

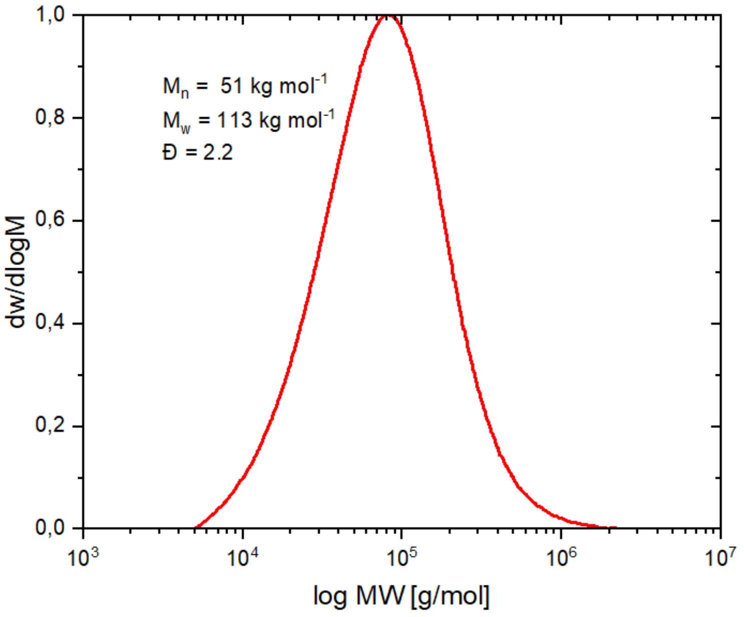

Figure S 121. HT-GPC trace of PE-18.18 used for blending with PP-26.

Molecular weight of PE-18.18 used for blending with PP-26 was determined by high temperature gel permeation chromatography (GPC) in 1,2-dichlorobenzene at 160 °C on a Polymer Char GPC-IR instrument, equipped with Agilent PLgel Olexis columns (3 x 30 cm, additional guard column), an infrared detector (IR5 MCT, concentration signal) and a viscosity detector. A standard flow rate of 1 mL min^{-1} was used. Molecular weights were determined via universal calibration versus low dispersity polystyrene standards from PSS Polymer Standards (software: PSS WinGPC, version 8.32).

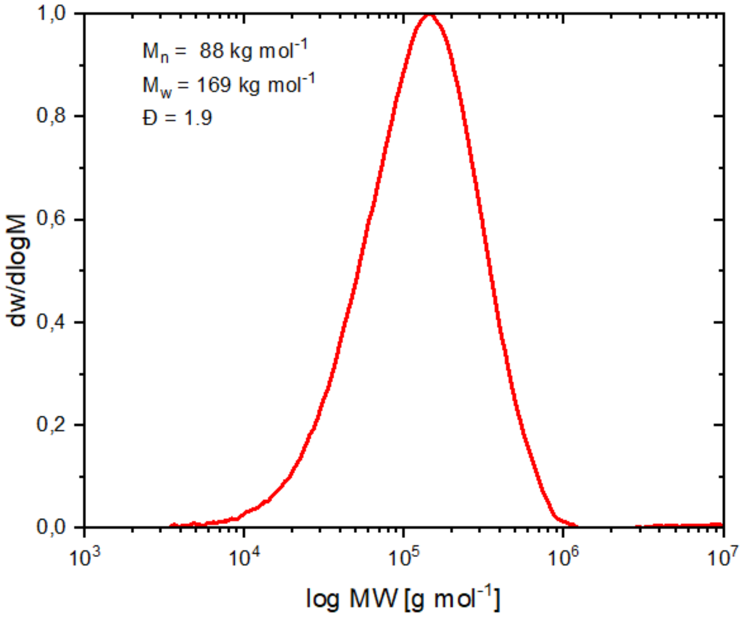

Figure S 122. GPC trace of PE-18.18 used for blending with PP-18.

S2. Supplementary Tables

Tensile properties of blends and of reference materials

Table S 2. Tensile properties of PE-18.18.

	Number of specimens	Youngs Modulus E [MPa]	Stress at yield σ_y [MPa]	Elongation at break ε_{tb} [%]
**virgin**	3	930 ± 10	21.9 ± 0.1	540 ± 20
**1 month at 60 % humidity**	3	970 ± 40	21.8 ± 0.3	500 ± 110
**4 months in water**	2	980 ± 70	19.6 ± 0.1	540 ± 20

Table S 3. Tensile properties of commercial HDPE.

	Number of specimens	Youngs Modulus E [MPa]	Stress at yield σ_y [MPa]	Elongation at break ε_{tb} [%]
**virgin**	3	1130 ± 10	20.4 ± 2.7	460 ± 20
**1 month at 60 % humidity**	3	1140 ± 20	18.4 ± 3.7	500 ± 50
**4 months in water**	2	1050 ± 0	17.9 ± 2.4	460 ± 30

Table S 4. Tensile properties of PE-18.18 blended with 2 wt% PP-26.

	Number of specimens	Youngs Modulus E [MPa]	Stress at yield σ_y [MPa]	Elongation at break ε_{tb} [%]
**virgin**	2	920 ± 160	21.5 ± 0.6	380 ± 0

Table S 5. Tensile properties of PE-18.18 blended with 5 wt% PP-26.

	Number of specimens	Youngs Modulus E [MPa]	Stress at yield σ_y [MPa]	Elongation at break ε_{tb} [%]
**virgin**	2	930 ± 100	19.4 ± 1.8	380 ± 10

Table S 6. Tensile properties of PE-18.18 blended with 10 wt% PP-26.

	Number of specimens	Youngs Modulus E [MPa]	Stress at yield σ_y [MPa]	Elongation at break ε_{tb} [%]
**virgin**	2	940 ± 10	19.7 ± 0.1	350 ± 10

Table S 7. Tensile properties of PE-18.18 blended with 20 wt% PP-26.

	Number of specimens	Youngs Modulus E [MPa]	Stress at yield σ_y [MPa]	Elongation at break ε_{tb} [%]
**virgin**	2	750 ± 10	16.8 ± 1.8	330 ± 10

Table S 8. Tensile properties of PE-18.18 blended with 0.5 wt% PP-18.

	**Number of specimens**	**Youngs Modulus E [MPa]**	**Stress at yield σ_y [MPa]**	**Elongation at break ε_{tb} [%]**
**virgin**	3	760 ± 180	19.2 ± 0.1	460 ± 40
**1 month at 60 % humidity**	3	990 ± 20	19.7 ± 0.2	400 ± 10
**4 months in water**	2	1140 ± 20	21.3 ± 0.3	90 ± 70
**3D printed**	2	600 ± 30	15.3 ± 0.1	290 ± 10

Table S 9. Tensile properties of PE-18.18 blended with 2 wt% PP-18. Tensile testing of the specimens immersed in water was not possible due to embrittlement.

	**Number of specimens**	**Youngs Modulus E [MPa]**	**Stress at yield σ_y [MPa]**	**Elongation at break ε_{tb} [%]**
**virgin**	3	840 ± 30	17.0 ± 0.1	440 ± 10
**1 month at 60 % humidity**	3	1240 ± 20	19.2 ± 2.2	10 ± 10
**4 months in water**	2	-	-	-

Table S 10. Tensile properties of PE-18.18 blended with 10 wt% PP-18. Tensile testing of the specimens immersed in water and stored at 60 % humidity was not possible due to embrittlement.

	**Number of specimens**	**Youngs Modulus E [MPa]**	**Stress at yield σ_y [MPa]**	**Elongation at break ε_{tb} [%]**
**virgin**	3	780 ± 30	20.2 ± 0.4	380 ± 50
**1 month at 60 % humidity**	3	-	-	-
**4 months in water**	2	-	-	-

Molecular weights of PE-18.18/PP-26 blends

Table S 11. Molecular weights of injection molded PE-18.18/PP-26 (2 wt%) blend specimens after 4, 8 and 12 months in buffer solution (pH = 7). The molecular weight of the virgin blend is shown for comparison. **Equation S 6** was employed to estimate the molecular weights of the PE-18.18 component of the blends by ^{1}H NMR end group analysis.

	M_n [kg/mol]	M_w [kg/mol]	Đ	M_n [kg/mol] (NMR)
**Virgin**	70.4	150.9	2.1	-
**4 months buffer**	10.9	23.4	2.1	10.4
**8 months buffer**	10.7	24.1	2.2	7.8
**12 months buffer**	10.8	23.8	2.2	7.7

Table S 12. Molecular weights of injection molded PE-18.18/PP-26 (5 wt%) blend specimens after 4, 8 and 12 months in buffer solution (pH = 7). The molecular weight of the virgin blend is shown for comparison. **Equation S 6** was employed to estimate the molecular weights of the PE-18.18 component of the blends by ^{1}H NMR end group analysis.

	M_n [kg/mol]	M_w [kg/mol]	Đ	M_n [kg/mol] (NMR)
**Virgin**	70.3	155.5	2.2	-
**4 months buffer**	10.4	19.3	1.8	7.4
**8 months buffer**	9.9	17.9	1.8	5.0
**12 months buffer**	8.7	18.1	2.1	6.6

Table S 13. Molecular weights of injection molded PE-18.18/PP-26 (10 wt%) blend specimens after 4, 8 and 12 months in buffer solution (pH = 7). The molecular weight of the virgin blend is shown for comparison. **Equation S 6** was employed to estimate the molecular weights of the PE-18.18 component of the blends by ^{1}H NMR end group analysis.

	M_n [kg/mol]	M_w [kg/mol]	Đ	M_n [kg/mol] (NMR)
**Virgin**	60.4	170.4	2.8	-
**4 months buffer**	7.2	14.4	2.0	7.1
**8 months buffer**	7.1	14.8	2.1	6.0
**12 months buffer**	7.0	15.0	2.1	5.0

Table S 14. Molecular weights of injection molded PE-18.18/PP-26 (20 wt%) blend specimens after 4, 8 and 12 months in buffer solution (pH = 7). The molecular weight of the virgin blend is shown for comparison. **Equation S 6** was employed to estimate the molecular weights of the PE-18.18 component of the blends by ^{1}H NMR end group analysis.

	M_n [kg/mol]	M_w [kg/mol]	Đ	M_n [kg/mol] (NMR)
**Virgin**	61.3	180.7	2.9	-
**4 months buffer**	7.1	15.9	2.2	7.0
**8 months buffer**	7.0	15.6	2.2	5.0
**12 months buffer**	6.6	14.6	2.2	4.6

Molecular weights of PE-18.18/PP-18 blends

Table S 15. Molecular weights of injection molded PE-18.18/PP-18 (0.5, 2 and 10 wt%) blend specimens after 4 months in buffer solution (pH = 8). The molecular weight of the virgin blends is shown for comparison. **Equation S 6** was employed to estimate the molecular weights of the hydrolyzed blends by ^{1}H NMR end group analysis. Integration of the carboxylic acid end group was not possible for the PE-18.18/PP-18 (0.5 wt%) blend due to its low intensity.

	**M_n [kg/mol]**	**M_w [kg/mol]**	**Đ**	**M_n [kg/mol] (NMR)**
**0.5 wt% PP-18 virgin**	65.4	146.1	2.2	-
**0.5 wt% PP-18 4 months buffer**	46.0	93.1	2.0	-
**2 wt% PP-18 virgin**	52.9	120.2	2.3	-
**2 wt% PP-18 4 months buffer**	11.4	24.7	2.2	6.0
**10 wt% PP-18 virgin**	71.9	160.1	2.2	-
**10 wt% PP-18 4 months buffer**	7.7	17.2	2.2	6.6

Crystallinity of blends

Table S 16. Crystallinity of virgin injection molded PE-18.18/PP-26 blend specimens and of the specimens stored in buffer solution (pH = 7) for 4, 8 and 12 months. The coefficients of determination of the fits are given in brackets. Peak deconvolution and fitting was not possible for the PE-18.18/PP-26 (10 wt%) blend stored in buffer for 4 months.

	**Crystallinity χ [%] Virgin**	**Crystallinity χ [%] 4 months buffer**	**Crystallinity χ [%] 8 months buffer**	**Crystallinity χ [%] 12 months buffer**
**PE-18.18/PP-26 (2 wt%)**	69 (0.994)	83 (0.983)	79 (0.986)	84 (0.989)
**PE-18.18/PP-26 (5 wt%)**	69 (0.994)	89 (0.988)	89 (0.986)	86 (0.990)
**PE-18.18/PP-26 (10 wt%)**	72 (0.994)	-	89 (0.987)	85 (0.988)
**PE-18.18/PP-26 (20 wt%)**	70 (0.996)	87 (0.987)	83 (0.990)	88 (0.992)

Table S 17. Crystallinity of virgin injection molded PE-18.18/PP-18 blend specimens and of the specimens stored in buffer solution (pH = 8) for 4 months. The coefficients of determination of the fits are given in brackets.

	**Crystallinity χ [%] Virgin**	**Crystallinity χ [%] 4 months buffer**
**PE-18.18/PP-18 (0.5 wt%)**	69 (0.994)	82 (0.994)
**PE-18.18/PP-18 (2 wt%)**	73 (0.991)	82 (0.991)
**PE-18.18/PP-18 (10 wt%)**	65 (0.994)	87 (0.989)

Weight changes of the blend samples exposed to aqueous media

Table S 18. Weight changes of the PE-18.18/PP-26 blend samples exposed to different aqueous media for 4 months in wt%.

	2 wt% PP-26	5 wt% PP-26	10 wt% PP-26	20 wt% PP-26
**4 months MilliQ**	0.16	0.35	0.35	-1.14
**4 months 0.5 M H_2SO_4**	0.08	0.35	0.49	-0.73
**4 months 1 M NaOH**	0.09	0.25	-3.53	-0.20
**4 months buffer (pH = 7)**	0.19	0.00	0.34	-1.06

Table S 19. Weight changes of the PE-18.18/PP-26 blend samples exposed to different hydrolysis media for 8 months in wt%.

	2 wt% PP-26	5 wt% PP-26	10 wt% PP-26	20 wt% PP-26
**8 months MilliQ**	0.36	0.54	0.28	-1.09
**8 months 0.5 M H_2SO_4**	0.34	0.55	0.64	-0.89
**8 months 1 M NaOH**	0.23	-0.28	-2.50	-1.78
**8 months buffer (pH = 7)**	0.35	0.61	0.22	-1.02

Table S 20. Weight changes of the PE-18.18/PP-26 blend samples exposed to different hydrolysis media for 12 months in wt%.

	2 wt% PP-26	5 wt% PP-26	10 wt% PP-26	20 wt% PP-26
**12 months MilliQ**	0.26	0.49	-0.23	-1.45
**12 months 0.5 M H_2SO_4**	0.23	0.17	0.16	-1.23
**12 months 1 M NaOH**	0.41	-26.93	-26.81	-29.20
**12 months buffer (pH = 7)**	0.22	0.43	-0.18	-1.30

Table S 21. Weight changes of the PE-18.18/PP-18 blend samples exposed to different hydrolysis media for 4 months in wt%.

	**0.5 wt% PP-18**	**2 wt% PP-18**	**10 wt% PP-18**
**4 months MilliQ**	0.06	0.34	0.08
**4 months 0.5 M H_2SO_4**	0.21	0.31	0.47
**4 months 1 M NaOH**	0.10	0.33	-1.75
**4 months buffer (pH = 8)**	0.09	0.35	0.07

References

(1) Häußler, M.; Eck, M.; Rothauer, D.; Mecking, S. Closed-loop recycling of polyethylene-like materials. *Nature* **2021**, *590* (7846), 423–427. DOI: 10.1038/s41586-020-03149-9.
